# Comparison of Extraction, Isolation, Purification, Structural Characterization and Immunomodulatory Activity of Polysaccharides from Two Species of *Cistanche*

**DOI:** 10.3390/molecules30244754

**Published:** 2025-12-12

**Authors:** Jingya Ruan, Juan Zhang, Lequan Yu, Ping Zhang, Anxin Chen, Dongmei Wang, Yi Zhang, Tao Wang

**Affiliations:** 1 Tianjin Key Laboratory of TCM Chemistry and Analysis, Tianjin University of Traditional Chinese Medicine, 10 Poyanghu Road, West Area, Tuanbo New Town, Jinghai District, Tianjin 301617, China; ruanjingya@tjutcm.edu.cn (J.R.); 18822575726@163.com (L.Y.); zp10259611@163.com (P.Z.); 2 Xinjiang Institute of Materia Medica, 18 Zhengyang Road, High-Tech Industrial Development Zone (Xinshi District), Urumqi 830017, China; ezhangjuane76@sina.com; 3 Xinjiang LifeCore High-Tech Co., Ltd., 55 Dongrong Road, Urumqi High-Tech Industrial Development Zone (Xinshi District), Urumqi 830017, China; cnguorongchen@sina.com (A.C.); smhl57624231@sina.com (D.W.)

**Keywords:** *Cistanche*, polysaccharide, structural characterization, structural–activity relationship, nitric oxide

## Abstract

This study focuses on polysaccharides from *Cistanche deserticola* and *Cistanche tubulosa*, medicinal plants renowned for their health benefits. The “water extraction and alcohol precipitation” method was used to obtain the crude polysaccharides of the wine-making residues of *C. deserticola* (CDP) and *C. tubulosa* (CTP), respectively. Then, ultrafiltration membrane (UFM), DEAE-52, and Sephadex-G75 or Smartdex-G100 gel chromatography were used to separate and purify the crude polysaccharides, yielding the homogeneous fractions **CDP1-5-1**, **CDP2-2-2**, **CDP2-3-2**, **CTP1-5-1**, and **CTP1-5-3**. Structural analysis was conducted by using Fourier-transform infrared spectroscopy (FT-IR), high-performance anion-exchange chromatography coupled with multi-angle laser light scattering and refractive index detection (HPAEC-MALLS-RID), gas chromatography–mass spectrometry (GC-MS), nuclear magnetic resonance (NMR), congo red, and scanning electron microscopy (SEM). **CDP1-5-1** was found to be an arabinan, while **CDP2-2-2** and **CDP2-3-2** were agavin-like fructans with different molecular weights. **CTP1-5-1** and **CTP1-5-3** were identified as a heteropolysaccharide and a galacturonan, respectively. Immunological evaluation using RAW264.7 macrophages showed that they all significantly enhanced nitric oxide (NO) production, with **CDP1-5-1** exhibiting the most potent activity. The structural–activity relationship is summarized as follows: the arabinose was a key active unit with NO stimulatory effects. This research provides foundational data on the structure and immune-enhancing potential of *Cistanche* polysaccharides, supporting their further development and application.

## 1. Introduction

Plant-based Traditional Chinese Medicine (TCM) represents a rich source of bioactive compounds, with polysaccharides being one of the primary components responsible for their pharmacological efficacy [1,2]. These natural polymers, composed of various monosaccharides linked by glycosidic bonds, exhibit diverse biological activities, including significant immunomodulatory effects, while maintaining a high safety profile with low toxicity [3,4,5]. Consequently, the extraction, structural characterization, and biological evaluation of polysaccharides from medicinal plants remain a key research focus.

The genus *Cistanche* (Orobanchaceae) includes medically valuable species, with the dried fleshy stems of *Cistanche deserticola* Y.C. Ma and *C. tubulosa* (Schenk) R. Wight recorded as genuine medicinal materials in the *Chinese Pharmacopoeia* [6]. As a traditional precious Chinese medicinal herb, *C. deserticola* is known for effects like tonifying kidney *yang* and replenishing essence and blood [7]. In recent years, the rising popularity of *Cistanche*-based health products, such as tea and wine (mostly prepared by soaking *Cistanche* in 52–60% ethanol in folk practice), has driven market demand, posing a risk of depletion for wild *C. deserticola* populations [7]. To alleviate resource pressure, two strategies are critical: expanding artificial cultivation and realizing sustainable utilization of post-processing residues (e.g., wine-making byproducts) by extracting their active components. However, research on valorizing these residues remains limited, creating a gap between industrial waste and resource reuse.

Polysaccharides are key bioactive components in plant-based TCM, contributing significantly to pharmacological efficacy through their unique structural characteristics [6]. For *Cistanche*, polysaccharide content reaches up to 13% in *C. deserticola* and 8.44–10.36% in *C. tubulosa* [8]. Existing studies confirm that crude polysaccharides from *C. deserticola* enhance immune cell phagocytosis, promote cytokine release, and activate macrophages [9,10]. However, three critical research gaps persist: 1. Most studies focus on crude polysaccharides of *C. deserticola*, with limited systematic characterization of purified polysaccharides from *C. tubulosa*, another authentic species in the *Chinese Pharmacopoeia*, let alone their wine-making residues; 2. The structure-activity relationship (SAR) of *Cistanche* polysaccharides (e.g., how monosaccharide composition or glycosidic linkage affects immunomodulation) remains unclear, restricting understanding of their mechanisms; 3. The immunomodulatory potential of polysaccharides from *Cistanche* residues has not been explored, leaving industrial waste underutilized.

Innate immunity serves as the first line of defense against pathogens, and its functional integrity is crucial for maintaining immune homeostasis. However, innate immunity can be compromised by genetic, environmental, metabolic, or pathological factors, leading to diminished immune surveillance and dysregulated defense responses. Such immunodeficient states are closely associated with various pathological conditions, including infectious diseases, tumorigenesis, chronic inflammation, and oxidative stress-related disorders [11]. Currently, there remains a lack of direct and effective clinical strategies to enhance innate immune function. Therefore, identifying novel active substances capable of specifically activating, training, or modulating the innate immune system holds significant clinical value and application potential.

Macrophages are central to innate immunity, acting as a first line of defense by releasing immune mediators such as nitric oxide (NO), a well-recognized indicator of macrophage activation [12,13,14]. Since polysaccharide bioactivity is closely tied to structural features (monosaccharide composition, glycosidic linkage, molecular weight) [15,16], elucidating the SAR of purified *Cistanche* polysaccharides via NO production assays is essential to clarify their immunomodulatory mechanisms. Additionally, as a medicinal and edible plant, *Cistanche* has significant potential for development into functional foods and pharmaceuticals [17], making residue-based polysaccharide research both scientifically meaningful and industrially valuable.

To address the above gaps, this study targets the wine-making residues of *C. deserticola* and *C. tubulosa*. The specific aims are: (1) Extract crude polysaccharides from the residues using the “water extraction and alcohol precipitation” method; (2) Purify the polysaccharides via ultrafiltration membrane (UFM), DEAE-52 anion-exchange chromatography, and Sephadex G-100 gel filtration; (3) Characterize the structures of purified fractions using Fourier transform infrared spectroscopy (FT-IR), high performance anion-exchange chromatography coupled with multi-angle laser light scattering and refractive index detection (HPAEC-MALLS-RID), gas chromatography-mass spectrometry (GC-MS), nuclear magnetic resonance (NMR), congo red, and scanning electron microscope (SEM), and chemical methods; (4) Evaluate their immunomodulatory activity by measuring NO release in RAW264.7 macrophages; (5) Establish preliminary SAR by comparing structural features and bioactivities of polysaccharides from the two species.

This research aims to provide a scientific basis for applying *Cistanche* residues in pharmaceuticals and functional foods, supporting the sustainable exploitation of this valuable medicinal resource.

## 2. Results

### 2.1. Preparation of Glycans from CD and CT

**Figure 1:**
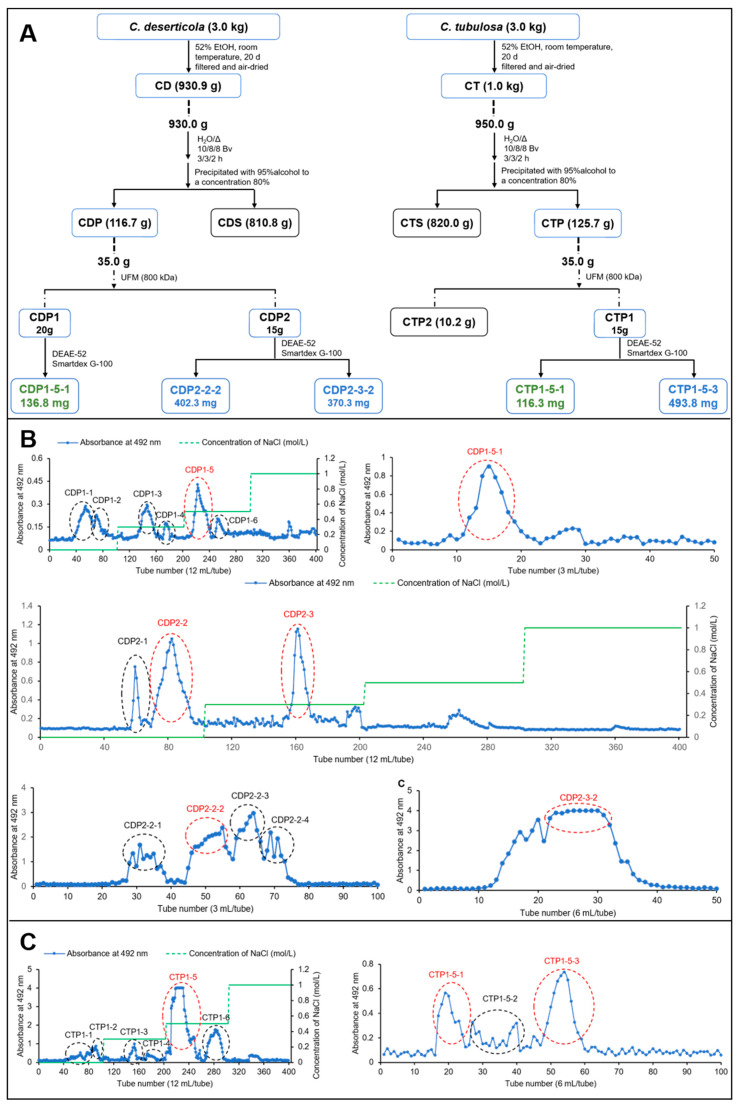
Separation diagram of CDP and CTP. (**A**) Extraction, isolation and purification process of CD and CT; (**B**) DEAE-52 and Sephadex G-75 separation diagram of CDP; (**C**) DEAE-52 and Sephadex G-75 separation diagram of CTP.

The crude polysaccharides of *C. deserticola* (CDP) and *C. tubulosa* (CTP) were fractionated using UFM technique, yielding CDP1 (>800 kDa, 20.3 g) and CDP2 (<800 kDa, 15.0 g), as well as CTP1 (>800 kDa, 15.8 g) and CTP2 (<800 kDa, 10.2 g). The elution profile from DEAE-cellulose column chromatography is shown in Figure 1. Based on the chromatographic results, the fraction eluted with 0.5 M NaCl was identified as the major component in CDP1, CDP2, and CTP1, and was therefore collected for further purification. Ultimately, the water-soluble glycans **CDP1-5-1** (136.8 mg), **CDP2-2-2** (402.3 mg), **CDP2-3-2** (370.3 mg), **CTP1-5-1** (116.3 mg), and **CTP1-5-3** (493.8 mg) were obtained.

### 2.2. Structure Characterization

#### 2.2.1. Structure Characterization of **CDP1-5-1**

**Figure 2:**
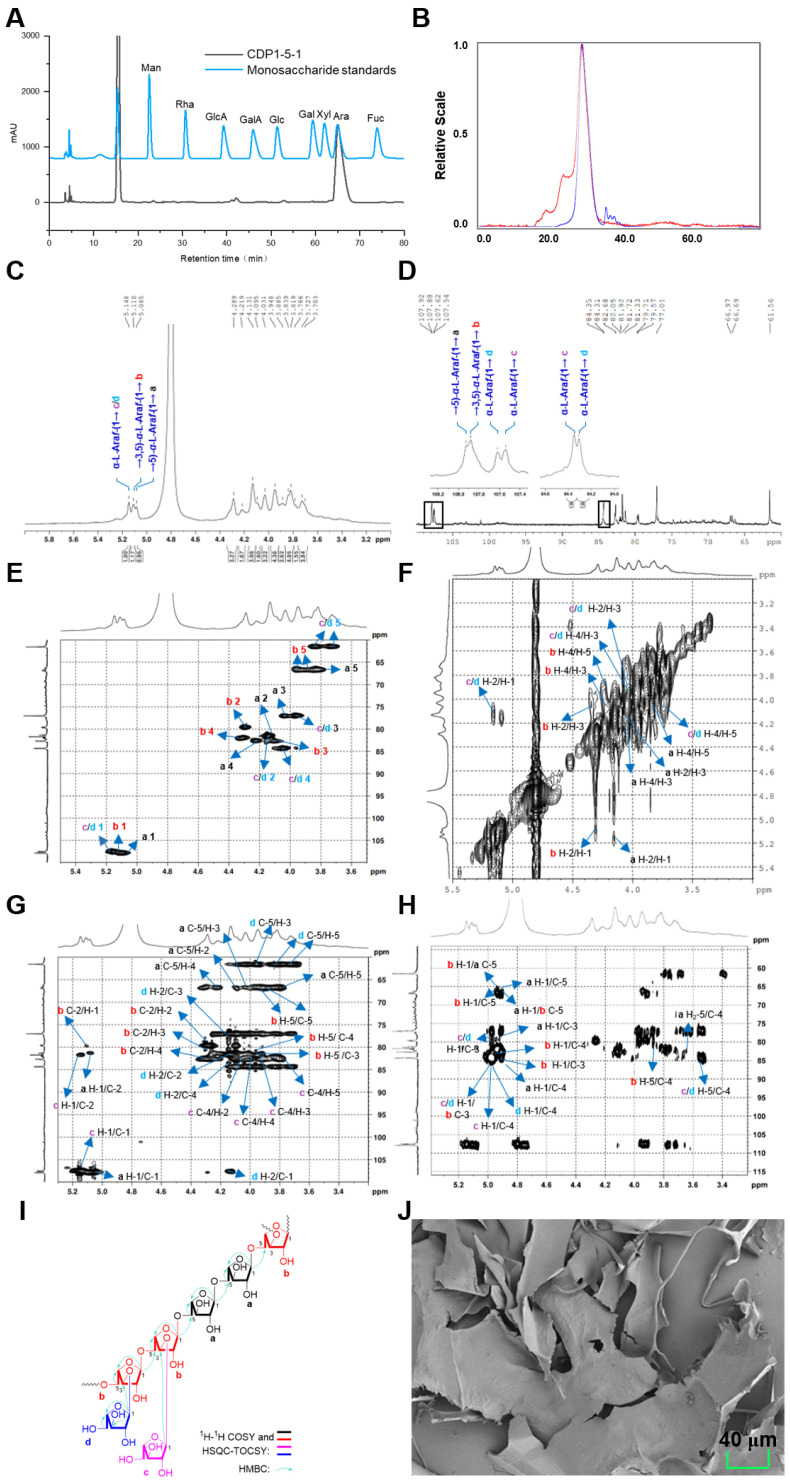
Structural characterization of **CDP1-5-1**. (**A**) Monosaccharide composition analysis; (**B**) HPGPC-MALLS-RID spectrum (Red line: signal detected by LS detector; Blue line: signal detected by RID detector); (**C**) ^1^H NMR spectrum; (**D**) ^13^C NMR spectrum; (**E**) HSQC spectrum; (**F**) ^1^H-^1^H COSY spectrum; (**G**) HSQC-TOCSY spectrum; (**H**) HMBC spectrum; (**I**) main 2D NMR correlations; (**J**) SEM images (500-times magnification).

Monosaccharide composition was analyzed by high-performance liquid chromatography (HPLC) with pre-column derivatization using 1-phenyl-3-methyl-5-pyrazolone (PMP). The analysis revealed that **CDP1-5-1** was predominantly composed of arabinose (Ara), as shown in Figure 2A.

The homogeneity and molecular weight of **CDP1-5-1** were assessed by HPSEC-RID-MALLS. As depicted in Figure 2B, the chromatogram displayed a single symmetrical peak, indicating a homogeneous sample. The weight-average molecular weight (Mw) was determined to be 852.9 kDa, and the number-average molecular weight (Mn) was recorded as 766.2 kDa (Table 1). Furthermore, a polydispersity index (Mw/Mn) of 1.1 was calculated, confirming a narrow molecular weight distribution.

The FT-IR spectrum of **CDP1-5-1** (Figure S1) exhibited characteristic absorption bands typical of polysaccharide structures. Key features included hydroxyl stretching vibration at 3399 cm^−1^, C-H stretching vibration at 2946 cm^−1^, O-H bending vibration at 1602 cm^−1^, C-O-C bending vibration at 1145 and 1019 cm^−1^.

The linkage pattern of **CDP1-5-1** was elucidated through methylation analysis followed by GC-MS (Figure S4). Interpretation of the mass spectra in conjunction with monosaccharide composition data identified three primary arabinofuranosyl (Ara*f*) residues: terminal-L-Ara*f* (t-L-Ara*f*), → 5)-L-Ara*f*-(1 →, and → 3,5)-L-Ara*f*-(1 →, eluting at 25.8, 29.3, and 31.1 min, respectively. Their molar ratio was determined to be 1.0:1.2:1.3 (Table 2).

The structure of **CDP1-5-1** was further elucidated through comprehensive NMR spectroscopic analysis. Examination of the ^1^H, ^13^C NMR, and HSQC spectra (Figure 2C–H, Table 3), in conjunction with GC-MS data and literature reports [18,19], enabled the assignment of key cross-peaks. Specifically, the signals at δ_H/C_ 5.09/107.9 and 5.11/107.9 were attributed to → 5)-α-L-Ara*f*-(1 → (**a**) and → 3,5)-α-L-Ara*f*-(1 → (**b**), respectively, while δ_H/C_ 5.15/107.5/107.6 was identified as two slightly different environments of α-L-Ara*f*-(1 → (**c** and **d**). In the ^1^H-^1^H COSY spectrum (Figure 2F), the following correlation signals were observed: δ_H/H_ 4.06/3.97 (**a** H-2/**a** H-3), 4.06/5.02 (**a** H-2/**a** H-1), 4.15/3.76 (**a** H-4/**a** H_2_-5), 4.15/3.97 (**a** H-4/**a** H-3), 4.23/4.04 (**b** H-2/**b** H-), 4.23/5.05 (**b** H-2/b H-1), 4.24/3.82, 3.89 (**b** H-4/**b** H_2_-5), 4.24/4.04 (**b** H-4/**b** H-3), 4.07/3.89 (**c** H-2/**c** H-3), 4.07/5.09 (**c** H-2/**c** H-1), 3.97/3.66, 3.78 (**c** H-4/**c** H_2_-5), 3.97/3.89 (**c** H-4/**c** H-3), 4.07/3.89 (**d** H-2/**d** H-3), 4.07/5.09 (**d** H-2/**d** H-1), 3.66, 3.78/3.97 (**d** H_2_-5/**d** H-4). In the HSQC-TOCSY spectrum, the following correlation signals were observed: δ_H/C_ 5.02/81.8/108.5 (**a** H-1/**a** C-2/**a** C-1); δ_C/H_ 67.2/3.76/3.97/4.06/4.15 (**a** C-5/**a** H_2_-5/**a** H-3/**a** H-2/**a** H-4); δ_C/H_ 80.1, 80.2/4.04/4.23/4.24/5.05 (**b** C-2/**b** H-3/**b** H-2/**b** H-4/**b** H-1); δ_H/C_ 3.82, 3.89/66.8, 67.5 (**b** H_2_-5/**b** C-5); δ_H/C_ 3.82, 3.89/82.6/83.2 (**b** H_2_-5/**b** C-4/**b** C-3); δ_H/C_ 5.09/82.2/108.1 (**c** H-1/**c C**-2/**c C**-1); δ_C/H_ 84.9/3.66, 3.78/3.89/3.97/4.07 (**c C**-4/**c** H_2_-5/**c** H-3/**c** H-4/**c** H-2); δ_H/C_ 4.07/77.5/82.2/84.8/108.1 (**d** H-2/**d** C-3/**d** C-2/**d** C-4/**d** C-1); δ_C/H_ 62.1/3.66, 3.78/3.89 (**d** C-5/**d** H_2_-5/**d** H-3) (Figure 2G). These spectra provided additional connectivity information for the sugar residues (Table 3). Key inter-residue correlations were observed in the HMBC spectrum: δ_H/C_ 5.09/77.0 (**a** H-1/C-3), 5.09/82.8 (**a** H-1/C-4), 3.82/82.8 (**a** H_2_-5/C-4), 5.11/82.1 (**b** H-1/C-4), 5.11/82.7 (**b** H-1/C-3), 3.89/82.7 (**b** H-5/C-3), 5.15/77.0 (**c** H-1/C-3), 5.15/84.4 (**c** H-1/C-4), 3.73/84.4 (**c** H-5/C-4), 5.15/77.0 (**d** H-1/C-3), 5.15/84.3 (**d** H-1/C-4), 3.73/84.3 (**d** H-5/C-4) (Figure 2I), yielding the structural fragments **a**, **b**, **c**, **d**. Crucially, HMBC correlations observed δ_H/C_ 5.09/66.7 (**a** H-1/**a** C-5), 5.09/66.3/67.0 (**a** H-1/**b** C-5), 5.11/66.3/67.0 (**b** H-1/**b** C-5), 5.11/66.7 (**b** H-1/**a** C-5), 5.15/82.7 (**c/d** H-1/**b** C-3) established that **CDP1-5-1** is an arabinan with a backbone of (1 → 5)-linked α-L-Ara*f*.

**Figure 3:**
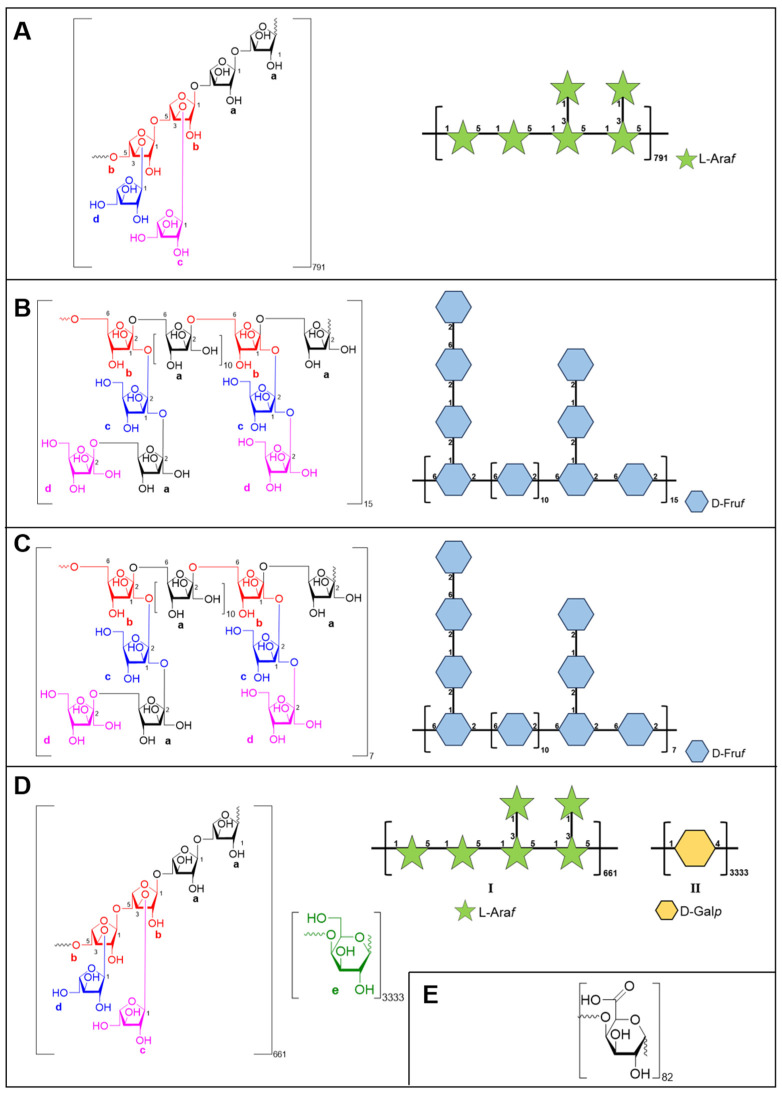
Structures of **CDP1-5-1** (**A**), **CDP2-2-2** (**B**), **CDP2-3-2** (**C**), **CTP1-5-1** (**D**), and **CTP1-5-3** (**E**).

Based on the integral ratio of anomeric protons in the ^1^H NMR spectrum (δ_H_ 5.09:5.11:5.15 = 0.9:0.9:1.0) and carbons in the ^13^C NMR spectrum (δ_C_ 84.3:84.4 = 1.0:1.1), the molar ratio of residues **a**:**b**:**c**:**d** was determined to be 2.0:2.0:1.0:1.0. Consequently, the structural repeating unit of **CDP1-5-1** was proposed. Considering the determined Mn of 766.2 kDa, **CDP1-5-1** is suggested to be a polymer comprising approximately 791 repeats of this structural unit (Figure 3A).

Congo red analysis result are shown in Figure S12. After mixing **CDP1-5-1** with Congo red solution, the maximum absorption wavelength (λ_max_) decreased gradually upon addition of NaOH solutions ranging from 0.1 to 0.6 M, accompanied by a slight red shift compared to the blank control. These results indicated that the **CDP1-5-1** formed a complex with Congo red but lacked a triple-helix structure.

The scanning electron microscope (SEM) images presented in Figure 2J indicated that **CDP1-5-1** exhibited an irregular flake-like morphology at 500× magnification. At a higher magnification of 10,000×, irregular particles attached to the surface were observed.

#### 2.2.2. Structure Characterization of **CDP2-2-2** and **CDP2-3-2**

**Figure 4:**
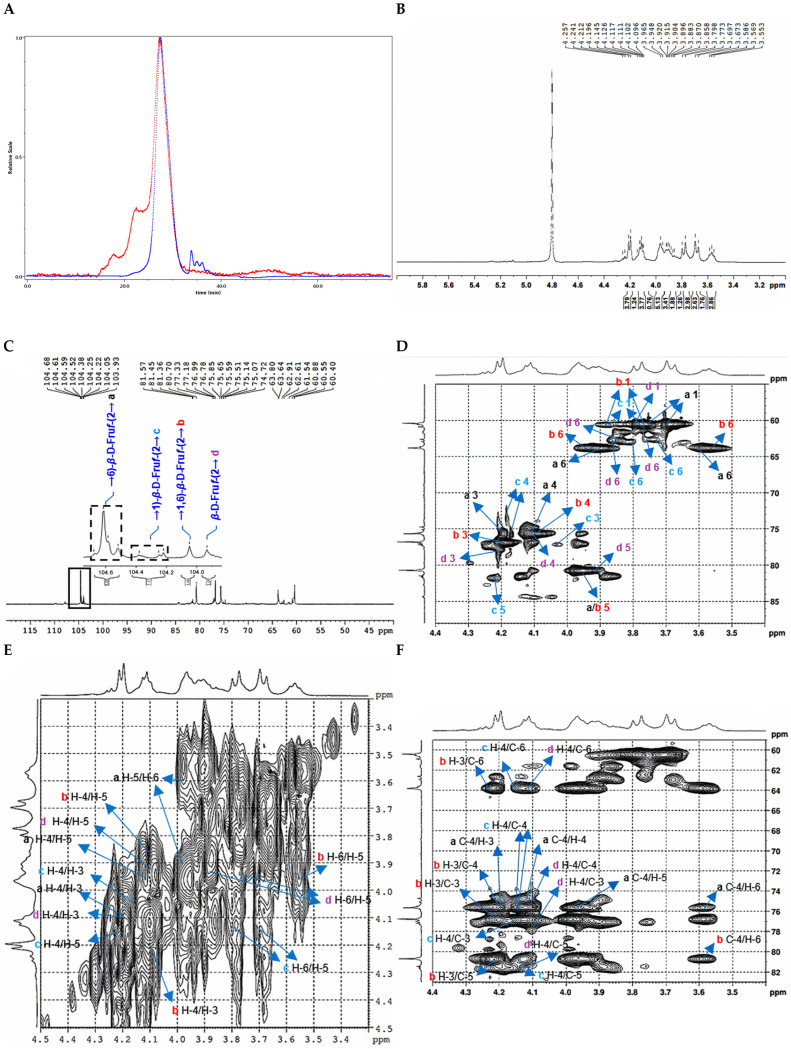

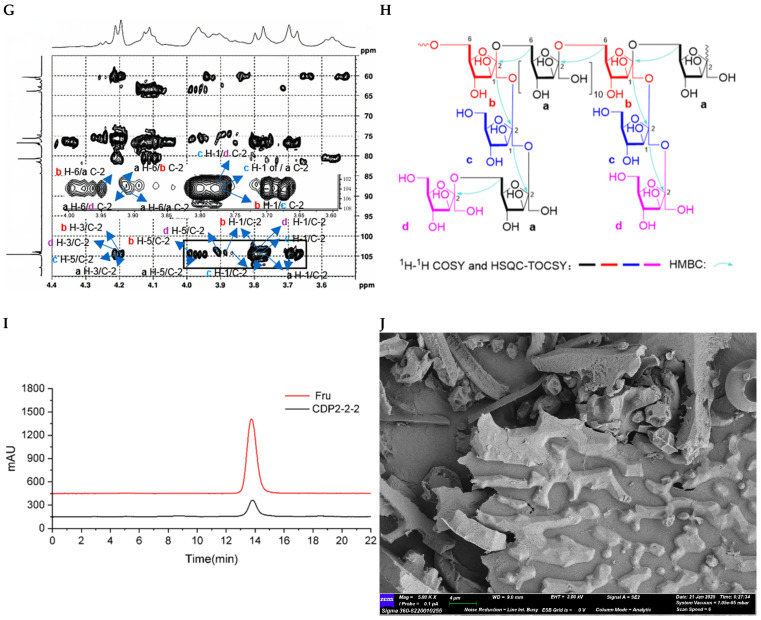
Structural characterization of **CDP2-2-2**. (**A**) HPGPC-MALLS-RID spectrum (Red line: signal detected by LS detector; Blue line: signal detected by RID detector); (**B**) ^1^H NMR spectrum; (**C**) ^13^C NMR spectrum; (**D**) HSQC spectrum; (**E**) ^1^H-^1^H COSY spectrum; (**F**) HSQC-TOCSY spectrum; (**G**) HMBC spectrum; (**H**) main 2D NMR correlations; (**I**) Monosaccharide composition analysis; (**J**) SEM images (500-times magnification) (40 μm).

**CDP2-2-2** exhibited a single symmetrical peak in the HPSEC-RID-MALLS chromatogram (Figure 4A), demonstrating high purity. Molecular weight analysis gave a Mw of 39.2 kDa and a Mn of 29.7 kDa (Table 1), corresponding to a low polydispersity index (Mw/Mn = 1.3) that reflects a narrow molecular weight distribution.

As shown in the FT-IR spectrum of **CDP2-2-2** (Figure S13), the major absorption bands corresponded to fundamental polysaccharide vibrations. These included hydroxyl stretches (3336 cm^−1^), aliphatic C-H stretches (2942 cm^−1^), water-associated O-H bends (1642 cm^−1^), and asymmetric C-O-C stretches (1131, 1019 cm^−1^) in the fingerprint region, indicative of the glycosidic linkages.

The molecular architecture of **CDP2-2-2** was further characterized using multi-dimensional NMR spectroscopy. There is no distinct anomeric proton signals of aldoses were detected in the ^1^H NMR spectrum (Figure S16). Analysis of the ^1^H, ^13^C NMR, and HSQC spectra (Figure 4B–G, Table 4), integrated with GC-MS results and published references [20,21,22], allowed unambiguous assignment of critical NMR signals. The signals at δ_C_ 104.5/104.6, 104.1, 104.2/104.4, and 103.9 were assigned to → 6)-β-D-Fru*f*-(2 → (**a**), → 1,6)-β-D-Fru*f*-(2 → (**b**), → 1)-β-D-Fru*f*-(2 → (**c**), and β-D-Fru*f*-(2 → (**d**), respectively. These assignments were corroborated by ^1^H-^1^H COSY (Figure 4E) and HSQC-TOCSY (Figure 4F) spectra, which provided supplementary through-bond connectivity evidence for the saccharide units (Table 4). Long-range heteronuclear correlations observed in the HMBC spectrum (Figure 4G) were critical for structural assignment. Key correlations included δ_H/C_ 3.70, 3.77/104.5/104.6 (**a** H_2_-1/**a** C-2), 4.20/104.5/104.6 (**a** H-3/**a** C-2), 3.97/104.5/104.6 (**a** H-5/**a** C-2), 3.77, 3.87/104.1 (**b** H_2_-1/**b** C-2), 4.21/104.1 (**b** H-3/**b** C-2), 3.97/104.1 (**b** H-5/**b** C-2), 3.76/104.2/104.4 (**c** H-1/**c C**-2), 4.21/104.2/104.4 (**c** H-5/**c C**-2), 3.77, 3.80/103.9 (**d** H_2_-1/**d** C-2), 4.21/103.9 (**d** H-3/**d** C-2), 3.92/103.9 (**d** H-5/**d** C-2), enabling the identification of structural units **a**–**d**. Most significantly, the through-bond couplings δ_H/C_ 3.90/104.5/104.6 (**a** H-6/**a** C-2), 3.90/104.1 (**a** H-6/**b** C-2), 3.90/103.9 (**a** H-6/**d** C-2), 3.77, 3.87/104.2/104.4 (**b** H_2_-1/**c C**-2), 3.95/104.5/104.6 (**b** H-6/**a** C-2), 3.76/104.5/104.6 (**c** H-1/**a** C-2), and 3.76/103.9 (**c** H-1/**d** C-2), provided definitive evidence that **CDP2-2-2** is a fructan with a (2 → 6)-linked β-D-Fru*f* backbone (Figure 4H).

Since ketoses exhibit low reducibility and do not efficiently derivatize with PMP under standard conditions [23], their presence could not be confirmed by the initial method. To overcome this limitation, mild acid hydrolysis with trifluoroacetic acid (TFA) followed by direct HPLC analysis on a Sugar D column was employed [24]. As shown in Figure 4I, comparison with monosaccharide standards confirmed that **CDP2-2-2** contains fructose (Fru).

Methylation-GC-MS analysis was employed to determine the glycosidic linkage profile of **CDP2-2-2** (Figure S22, Table 2). Four major structural motifs of fructofuranosyl residues were identified based on retention times and mass fragmentation patterns comparing to the literature [25,26]: D-Fru*f*-(2 → (28.8 min), → 6)-D-Fru*f*-(2 → (32.2 min), → 1)-D-Fru*f*-(2 → (32.3 min), and → 1,6)-D-Fru*f*-(2 → (34.5 min).

According to quantitative ^13^C NMR analysis of anomeric carbon signals (δ_C_ 104.5/104.6:104.1:104.2/104.4:103.9 = 6.3:0.9:1.1:1.0), the molar ratio of glycosyl residues **a**:**b**:**c**:**d** was established. This stoichiometric relationship led to the proposed repeating unit structure presented in Figure 3C. Considering the Mn of 29.7 kDa determined by SEC-MALLS, **CDP2-2-2** appears to consist of approximately 15 iterations of this fundamental structural motif.

The Congo red binding assay results for **CDP2-2-2** are presented in Figure S24. Upon complexation with Congo red, the λmax of the mixture exhibited a progressive decrease as the NaOH concentration was increased from 0.1 to 0.6 M, together with a minor bathochromic shift relative to the control. This spectral pattern indicates the formation of a polysaccharide-Congo red complex while confirming the absence of a triple-helical conformation in **CDP1-5-1**.

As illustrated in Figure 4J, the maximum absorption wavelength of the reaction product showed no significant initial increase followed by a sharp decrease, suggesting the absence of a triple-helix conformation in **CDP2-2-2**.

**Figure 5:**
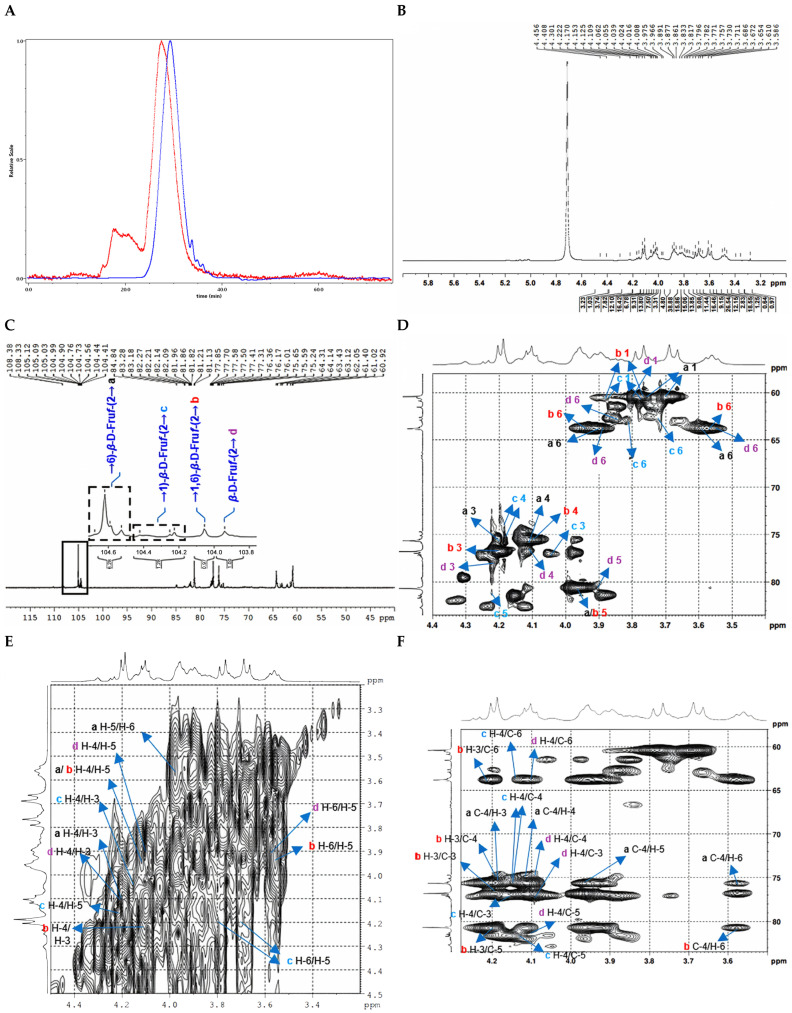

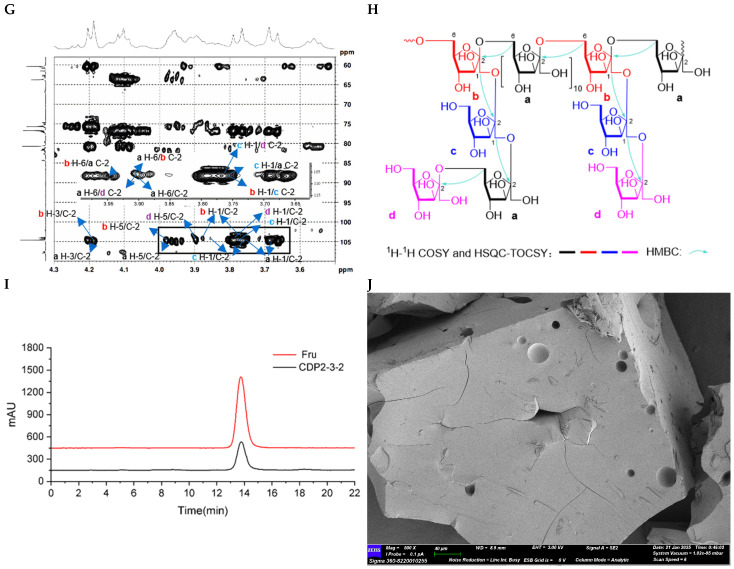
Structural characterization of **CDP2-3-2**. (**A**): HPGPC-MALLS-RID spectrum (Red line: signal detected by LS detector; Blue line: signal detected by RID detector); (**B**) ^1^H NMR spectrum; (**C**) ^13^C NMR spectrum; (**D**) HSQC spectrum; (**E**) ^1^H-^1^H COSY spectrum; (**F**) HSQC-TOCSY spectrum; (**G**) HMBC spectrum; (**H**) main 2D NMR correlations; (**I**) Monosaccharide composition analysis; (**J**) SEM images (500-times magnification).

**CDP2-2-2** and **CDP2-3-2** are structurally homologous fructans sharing an identical (2→6)-linked β-D-Fru*f* backbone and branching patterns. The principal distinction between them lies in their molecular weights, with **CDP2-2-2** (Mn 29.7 kDa) being approximately twice the size of **CDP2-3-2** (Mn 13.7 kDa), corresponding to a higher number of repeating units. Both polysaccharides adopt a compact, highly branched coil conformation and lack a triple-helix structure (Figure 3 and Figure 5, Table 5).

#### 2.2.3. Structure Characterization of **CTP1-5-1**

**Figure 6:**
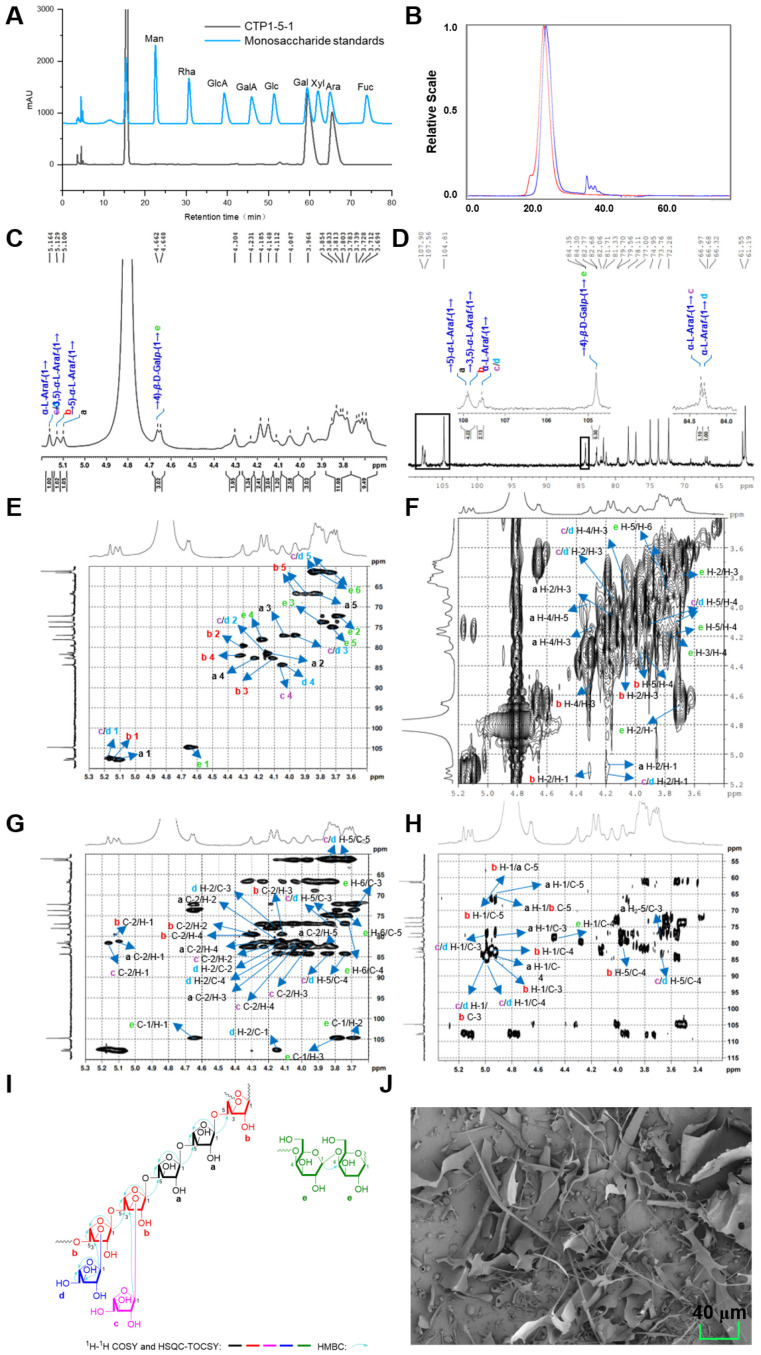
Structural characterization of **CTP1-5-1**. (**A**) Monosaccharide composition analysis; (**B**) HPGPC-MALLS-RID spectrum (Red line: signal detected by LS detector; Blue line: signal detected by RID detector); (**C**) ^1^H NMR spectrum; (**D**) ^13^C NMR spectrum; (**E**) HSQC spectrum; (**F**) ^1^H-^1^H COSY spectrum; (**G**) HSQC-TOCSY spectrum; (**H**) HMBC spectrum; (**I**) main 2D NMR correlations; (**J**) SEM images (500-times magnification) (40 μm).

Similar to **CDP1-5-1**, the FT-IR spectrum of **CTP1-5-1** (Figure S37) exhibited characteristic absorption peaks typical of polysaccharides. However, monosaccharide composition analysis (Figure 6A) revealed a fundamental difference between the two: while **CDP1-5-1** consists solely of Ara, **CTP1-5-1** is a heteropolysaccharide composed of galactose (Gal) and Ara in a ratio of 1.3:1.0.

Methylation and GC-MS analysis (Figure S40, Table 2) further confirmed its structural complexity. **CTP1-5-1** not only contained the same three arabinosyl residues as **CDP1-5-1**, namely, t-L-Ara*f*, → 5)-L-Ara*f*-(1 →, and → 3,5)-L-Ara*f*-(1 →, but also additionally identified a high proportion of → 4)-D-Gal*p*-(1 → residues (approximately 57%).

NMR analysis (Figure 6C–H, Table 6) not only verified the presence of these glycosyl residues but also, through key correlation signals in the HMBC spectrum, determined that **CTP1-5-1** comprises two main structural domains: one is a backbone of → 5)-α-L-Ara*f*-(1 → (**Domain I**), similar to the core of **CDP1-5-1**, and the other is a novel linear galactan region (**Domain II**) formed by → 4)-β-D-Gal*p*-(1 → linkages [18,27] (Figure 3D).

In terms of molecular size and conformation, both **CTP1-5-1** (Mw: 797.1 kDa, Mn: 640.7 kDa, polydispersity index (PDI): 1.2) and **CDP1-5-1** (Mw: 852.9 kDa, Mn: 766.2 kDa, PDI: 1.1) exhibited a homogeneous, tightly coiled conformation (with *v* values of 0.2 and 0.04, respectively). However, the higher PDI value and more complex types of glycosyl residues in **CTP1-5-1** suggest that its molecules possess a higher degree of branching and structural heterogeneity.

This structural difference is reflected in their morphologies. SEM images (Figure 6J) showed that **CTP1-5-1** also presented an irregular flaky structure, but the particles attached to its surface were denser and more diverse in shape. This contrasts with the morphology of **CDP1-5-1**, visually reflecting its more complex chemical composition. Congo red assay results (Figure S48) indicated that, consistent with **CDP1-5-1**, **CTP1-5-1** also lacks a triple-helix structure.

In summary, although **CTP1-5-1** shares a similar arabinan backbone and a comparable compact spherical conformation with **CDP1-5-1**, the unique feature of **CTP1-5-1** lies in the introduction of a dominant → 4)-β-D-Gal*p* domain, thereby forming a more complex, highly branched arabinogalactan composite structure.

#### 2.2.4. Structure Characterization of **CTP1-5-3**

**Figure 7:**
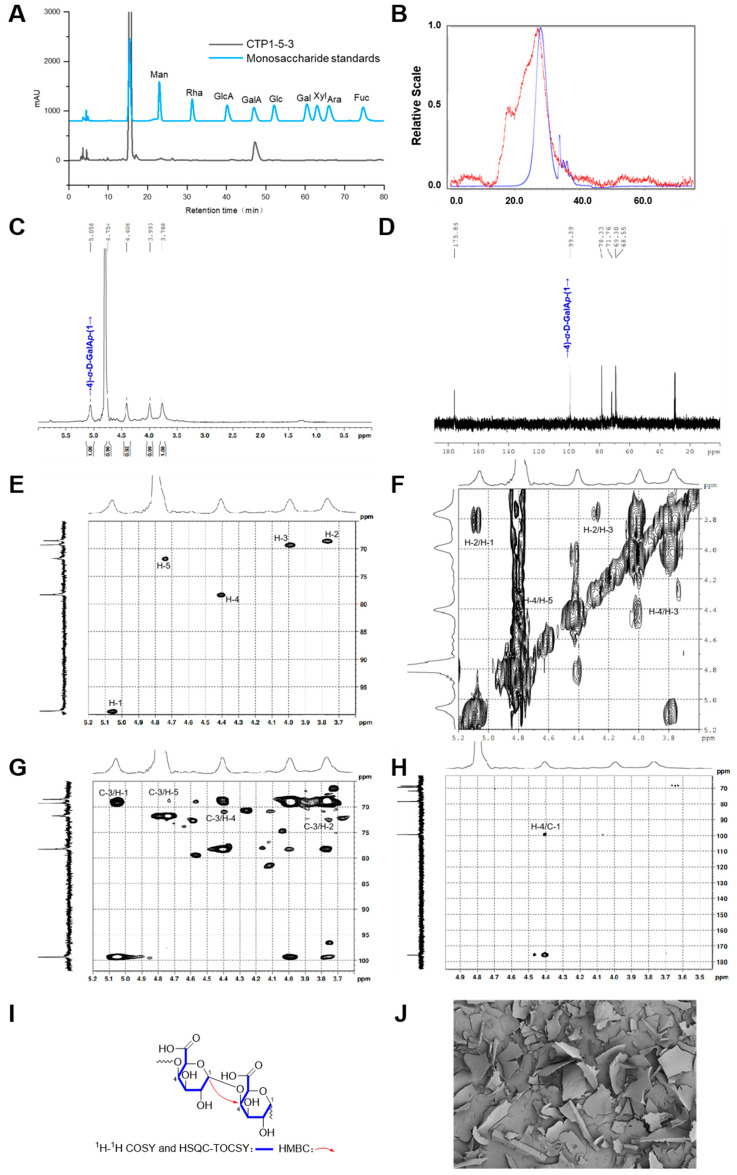
Structural characterization of **CTP1-5-3**. (**A**) Monosaccharide composition analysis; (**B**) HPGPC-MALLS-RID spectrum (Red line: signal detected by LS detector; Blue line: signal detected by RID detector); (**C**) ^1^H NMR spectrum; (**D**) ^13^C NMR spectrum; (**E**) HSQC spectrum; (**F**) ^1^H-^1^H COSY spectrum; (**G**) HSQC-TOCSY spectrum; (**H**) HMBC spectrum; (**I**) main 2D NMR correlations; (**J**) SEM images (500-times magnification) (40 μm).

The monosaccharide analysis results of **CTP1-5-3** indicated that it consisted solely of galacturonic acid (GalA) (Figure 7A).

The HPSEC-RID-MALLS chromatogram of **CTP1-5-3** (Figure 7B) exhibited a single symmetrical peak, indicating that it was a homogeneous polysaccharide. The molecular weight distribution and corresponding Mn of **CTP1-5-3** was 15.9 kDa (Table 1). The polydispersity index of **CTP1-5-3** was calculated to be 1.3 according to its Mw and Mn values, revealing its molecular weight distribution was narrow.

The glycosidic bond type in **CTP1-5-3** was primarily identified as → 4)-D-Gal*p*A-(1 → fragment (Figure 7C–H). Further analysis of **CTP1-5-3** based on ^13^C NMR spectrum revealed six carbon signals of consistent intensity, with δ_C_ 99.4 corresponding to the anomeric carbon signal. The signal at δ_C_ 78.3 was shifted nearly 8 ppm downfield from the C-4 of the free galacturonic acid group, indicating a → 4)-D-Gal*p*A-(1 → linkage type (Table 7). In the ^1^H NMR spectra of **CTP1-5-3** (Figure 7C), only one anomeric signal was observed at δ_H_ 5.06, suggesting a α-configuration. By comparing with literature [18], **CTP1-5-3** was finally identified as 1,4-α-D-galacturonan. Considering its Mn of 15.9 kDa, the degree of polymerization was determined to be 82. Thus, the structure of **CTP1-5-3** (Figure 3E) was identified.

The Congo red analysis result is shown in Figure S60. After mixing **CTP1-5-3** with Congo red solution, the λ_max_ decreased gradually upon addition of NaOH solutions ranging from 0.1 to 0.6 M, accompanied by a slight red shift compared to the blank control. These results indicated that the **CTP1-5-3** formed a complex with Congo red but lacked a triple-helix structure.

The SEM images presented in Figure 7J indicated that **CTP1-5-3** appeared as irregular flakes of varying sizes, with a rough surface featuring fine linear fissures and small pores.

### 2.3. Evaluation of Immunomodulatory Activity

**Figure 8:**
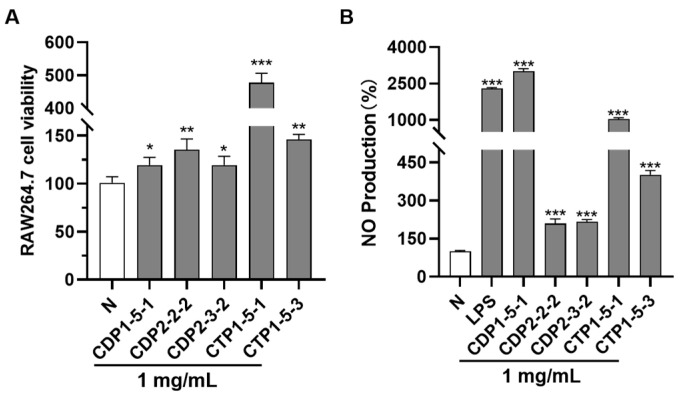
In vitro pharmacodynamic assay diagram. (**A**) Effects of **CDP1-5-1**, **CDP2-2-2**, **CDP2-3-2**, **CTP1-5-1** and **CTP1-5-3** on RAW264.7 cell viability at a concentration of 1 mg/mL; (**B**) Effect of **CDP1-5-1**, **CDP2-2-2**, **CDP2-3-2**, **CTP1-5-1** and **CTP1-5-3** on NO production in RAW264.7 cells. * *p* < 0.05; ** *p* < 0.01; *** *p* < 0.001 (Differences between compound-treated group and normal group).

To ensure the promoting effects of **CDP1-5-1**, **CDP2-2-2**, **CDP2-3-2**, **CTP1-5-1**, and **CTP1-5-3** on NO release from RAW264.7 cells were evaluated at non-cytotoxic concentrations, their potential cytotoxicity was first assessed using the CCK-8 assay. The results showed that none of the polysaccharides exhibited growth inhibition on RAW264.7 cells at a concentration of 1 mg/mL (Figure 8A, Table S1). Therefore, this concentration was selected as a safe dose for subsequent activity experiments.

The ability of these polysaccharides to enhance NO release was further investigated using the Griess assay. As shown in Figure 8B and Table S1, all tested compounds significantly promoted NO production at 1 mg/mL, with **CDP1-5-1** demonstrating markedly stronger activity compared to the others.

## 3. Discussion

This study systematically explored polysaccharides from the wine-making residues of CD and CT, successfully addressing the three core gaps proposed in the introduction: structural characterization of purified CT polysaccharides, clarification of SAR, and sustainable utilization of residues.

First, the structural characterization of five homogeneous polysaccharide fractions (**CDP1-5-1**, **CDP2-2-2**, **CDP2-3-2** derived from CD; **CTP1-5-1**, **CTP1-5-3** derived from CT) addresses key knowledge gaps in the following aspects: 1. **CDP1-5-1** (Mw: 852.9 kDa) is identified as an arabinan with a backbone of → 5)-α-L-Araf-(1 →, yet provides a clearly defined linear architecture, thereby laying the groundwork for subsequent activity studies; 2. **CDP2-2-2** (Mw: 39.2 kDa) and **CDP2-3-2** (Mw: 19.5 kDa) represent novel agavin-like fructans sharing an identical → 2)-β-D-Fruf-(6 → backbone and differing only in molecular weight. This constitutes the first report of such fructans from CD residues, significantly expanding the understanding of polysaccharide diversity in CD; 3. **CTP1-5-1** (Mw: 797.1 kDa) is characterized as a heteropolysaccharide (arabinan-galactan) with a → 5)-α-L-Araf-(1 → and → 4)-β-D-Galp-(1 → dual domain, while **CTP1-5-3** (Mw: 20.7 kDa) is identified as a → 4)-α-D-GalpA-(1 → galacturonan. These data provide the first detailed structural elucidation of purified CT polysaccharides, thereby offering scientific support for the recognition of CT as an authentic species in the Pharmacopoeia.

Second, immunomodulatory activity results (Figure 8B) further clarify the SAR of *Cistanche* polysaccharides: 1. All five fractions significantly enhanced NO release in RAW264.7 macrophages (1 mg/mL), with **CDP1-5-1** (arabinan) showing the strongest activity. This confirms arabinose as a key active monosaccharide unit, supported by **CTP1-5-1**’s reduced activity (due to galactan interference), which may block arabinose binding to macrophage receptors [28]; 2. **CDP2-2-2** and **CDP2-3-2** exhibited similar NO-stimulating activity despite a 2-fold molecular weight difference, indicating fructan activity is stable within the 10–40 kDa range. This provides a practical reference for industrial production (no strict molecular weight control required); 3. **CTP1-5-3** (galacturonan) had moderate activity, suggesting galacturonic acid contributes to immunomodulation but is less potent than arabinose.

When compared to polysaccharides from fresh *Cistanche* materials [4,29,30], the residue-derived polysaccharides in this study have distinct structures (e.g., characterized by the presence of arabinan and agavin-like fructans vs. pectin-like acidic polysaccharides in references). This difference may stem from alcohol soaking during wine-making, which enriches neutral polysaccharides (arabinans, fructans) by removing water-soluble impurities. It also implies residue polysaccharides have unique application potential distinct from fresh materials.

This study only evaluated immunomodulatory activity via NO production; future work should use Western blot and immunofluorescence to explore signaling pathways and macrophage polarization for polysaccharides like **CDP1-5-1**. In vivo studies using immunosuppressed mouse models are also needed to verify activity in living systems. Additionally, scale-up extraction experiments could support industrial application of the “residue-to-active-ingredient” strategy, further promoting *Cistanche*’s sustainable use.

## 4. Materials and Methods

### 4.1. Materials

The plant materials, *Cistanche deserticola* Y.C. Ma and *Cistanche tubulosa* (Schenk) R. Wight (authenticated by Professor Lin Ma, Tianjin University of Traditional Chinese Medicine, voucher specimen nos. 202210251 and 202210252) were supplied by Xinjiang Life Nuclear Power High-Tech Co., Ltd. (located in Xinjiang, China).

Chemical reagents and analytical standards were obtained from commercial suppliers. TFA (analytical grade) was supplied by Damao Co., Ltd. (Tianjin, China). Monosaccharide standards, including mannose (Man), rhamnose (Rha), and Gal, were procured from the National Institutes for Food and Drug Control. Additional standards, such as xylose (Xyl), Ara, glucose (Glc), GalA, Fru and glucuronic acid (GlcA), were acquired from Yuanye Co., Ltd. (Shanghai, China). A nitric oxide (NO) assay kit was sourced from Beyotime Biotechnology Co., Ltd. (Shanghai, China).

The murine macrophage cell line RAW264.7 was obtained from Procell Life Science & Technology Co., Ltd. (Wuhan, China).

### 4.2. Extraction and Purification of the Glycans from C. deserticola and C. tubulosa

Fresh *C. deserticola* and *C. tubulosa* materials (3.0 kg each) were cut into pieces and soaked in 52% edible alcohol at room temperature for 20 days, with stirring performed twice daily for 10 min each time. After soaking, the mixtures were filtered and air-dried to obtain CD (939.0 g) and CT (1.0 kg), respectively. CD (930.0 g) and CT (950.0 g) were then subjected to triple hot-water extraction (3 h, 2 h, and 2 h, respectively). The combined extracts were concentrated, filtered, and centrifuged. The supernatant was treated by adjusting the ethanol concentration to 80% and stored at 4 °C overnight, yielding the crude polysaccharides CDP (116.7 g) and CTP (125.7 g), respectively.

To purify the crude extracts, CDP and CTP were dissolved and subjected to ultrafiltration (800 kDa cutoff). The retained fractions (CDP1, CTP1) were lyophilized and then sequentially chromatographed on a DEAE-52 column (eluted with a 0–1.0 M NaCl gradient) and a Sephadex G-75 column (eluted with water). This yielded the final purified polysaccharides: **CDP1-5-1**, **CDP2-2-2**, **CDP2-3-2**, **CTP1-5-1**, and **CTP1-5-3**.

### 4.3. Structural Characterization of ***CDP1-5-1***, ***CDP2-2-2***, ***CDP2-3-2***, ***CTP1-5-1***, and ***CTP1-5-3***

#### 4.3.1. Fourier Transform Infra-Red Spectrometer Analysis

FT-IR spectra of the purified glycans (**CDP1-5-1**, **CDP2-2-2**, **CDP2-3-2**, **CTP1-5-1**, and **CTP1-5-3**) were acquired on a Varian 640-IR spectrometer (Tuopu, Ningbo, China). For analysis, each lyophilized sample (1–2 mg) was thoroughly mixed with 50 mg of KBr and compressed into a transparent disk for measurement, which was conducted over a wavenumber range of 4000 to 400 cm^−1^ at a resolution of 4 cm^−1^.

#### 4.3.2. Homogeneity and Molecular Weight Analysis

The molecular weight and homogeneity of the polysaccharides were determined using HPSEC-RID-MALLS (Waters, Yorba Linda, CA, USA). Separation was achieved on an Ohpak SB-805/803 HQ column (300 × 8 mm I.D.) maintained at 40 °C, using a 0.1 M NaNO_3_ aqueous solution (containing 0.02% NaN_3_, *w*/*w*) as the mobile phase at a flow rate of 0.6 mL/min. The HPSEC system was connected in series to a MALLS detector (DAWN HELEOS II, Waters, CA, USA) and a refractive index detector (Optilab T-rEX, Wyatt Technol.) for simultaneous analysis. For each injection, 100 μL of the polysaccharide solution (1 mg/mL) was loaded.

#### 4.3.3. Monosaccharide Composition Analysis

The monosaccharide compositions of **CDP1-5-1**, **CTP1-5-1**, and **CTP1-5-3** were analyzed by HPLC following pre-column derivatization with PMP. Briefly, each sample (1 mg) was hydrolyzed with 2 M TFA (1 mL) at 100 °C for 6 h. After drying under a stream of nitrogen, the hydrolysate was redissolved in 1 mL of deionized water. A 300 μL aliquot was then derivatized with 0.5 M PMP in methanol and 0.3 M NaOH at 70 °C for 100 min. The reaction was terminated by neutralization with 0.3 M HCl. The resulting mixture was extracted three times with chloroform to remove excess reagent. The aqueous layer was collected, centrifuged at 14,000 rpm for 20 min, and passed through a 0.45 μm membrane filter prior to HPLC analysis. Separation was performed on an Agilent 1290 system (Agilent, Santa Clara, CA, USA) equipped with a Cosmosil 5C18-MS-II column (4.6 × 250 mm, 5 μm), using an isocratic elution of 0.1 M phosphate-buffered saline (PBS, pH 6.8) and acetonitrile (83:17, *v*/*v*) at a flow rate of 0.7 mL/min.

The monosaccharide compositions of **CDP2-2-2** and **CDP2-3-2** were also determined using a method involving direct analysis after mild acid hydrolysis. In detail, 5 mg of **CDP2-2-2** and **CDP2-3-2** were hydrolyzed with 1 mL of 0.5 M TFA at 80 °C for 40 min. After the reaction, add anhydrous MeOH and evaporate to dryness under reduced pressure until the reaction product has no sour taste. Dissolve the above product in 1 mL of deionized water, centrifuge (14,000 rpm, 20 min), take the supernatant and pass it through a 0.45 μm microporous membrane, and analyze it by HPLC. The analysis conditions are as follows: the chromatographic column is a Cosmosil Sugar-D column (5 μm, 4.6 mm × 250 mm); the mobile phase is water/acetonitrile = 15:85 (*v*/*v*); the injection volume is 10 μL; the detector is an evaporative light scattering detector (ELSD); the flow rate is 0.7 mL/min; the column temperature is room temperature.

#### 4.3.4. Methylation Analysis

Glycosidic linkage analysis was performed according to the partially methylated alditol acetate (PMAA) method, with sample preparation involving sequential steps of methylation, acid hydrolysis, reduction, acetylation, and subsequent GC-MS detection.

Briefly, approximately 5 mg of each polysaccharide sample was dissolved in 10 mL of anhydrous dimethyl sulfoxide (DMSO) containing 4 Å molecular sieves. After adding 400 mg of sodium hydroxide, the mixture was sealed and sonicated until complete dissolution was achieved. Under a nitrogen atmosphere, 0.5 mL of iodomethane was added dropwise, and the methylation reaction was conducted under low-temperature ultrasonication for 30 min. This step was repeated three times, with the final reaction extended to 1 h.

Upon completion, the mixture was diluted with 5 mL of deionized water and extracted multiple times with dichloromethane (3 × 2 mL). The combined organic phase was evaporated to dryness under a gentle nitrogen stream. The methylated product was then hydrolyzed with 2 M (**CDP1-5-1**, **CTP1-5-1** and **CTP1-5-3**)/0.5 M (**CTP2-2-2** and **CTP2-3-2**) TFA (1 mL) at 100 °C for 6 h. The hydrolysate was reduced with 50 μL of 1 M sodium borodeuteride (NaBD_4_) at 40 °C for 2.5 h, after which the reaction was quenched by adding 20 μL of acetic acid. The resulting mixture was dried and acetylated with a 1:1 (*v*/*v*) mixture of acetic anhydride and pyridine (250 μL each) at 100 °C for 2.5 h. The acetylated derivatives were dried again, reconstituted in 1 mL of chloroform, and washed three times with an equal volume of distilled water to remove impurities.

The final PMAA derivatives were analyzed using an Agilent 7890B gas chromatograph coupled with an Agilent 5977B triple-axis mass spectrometer (Agilent, CA, USA). Separation was achieved on an HP-5 MS capillary column (2.1 mm × 100 mm, 1.7 μm film thickness) with the following temperature program: initial temperature 40 °C (held for 1 min), ramped to 280 °C at 5 °C/min, and held for 1 min. The injector was maintained at 250 °C and operated in split mode. High-purity helium was used as the carrier gas at a constant flow rate of 1.2 mL/min.

#### 4.3.5. NMR Spectroscopy Analysis

NMR spectroscopic analysis was conducted to characterize the structural features of **CDP1-5-1**, **CDP2-2-2**, **CDP2-3-2**, **CTP1-5-1**, and **CTP1-5-3**. Each sample (30 mg) was repeatedly exchanged with D_2_O (0.5 mL) and transferred into a 5 mm NMR tube. All spectra were acquired on a Bruker Avance III HD 500 MHz spectrometer (Bruker, Billerica, MA, USA). The experimental suite included one-dimensional spectra (^1^H and ^13^C NMR) as well as two-dimensional experiments (^1^H-^1^H COSY, HSQC, HSQC-TOCSY, and HMBC) to enable comprehensive signal assignment.

#### 4.3.6. Experimental Analysis of Congo Red

The potential triple-helical conformation of the polysaccharides was investigated using a Congo red binding assay. Briefly, 1 mL of each polysaccharide solution (1 mg/mL) was mixed with an equal volume of Congo red (80 μM). The alkalinity of the resulting mixtures was then adjusted by adding 1 M NaOH to obtain a series of final NaOH concentrations (0–0.6 M). A solution of Congo red mixed with distilled water served as the blank control. After equilibrating for 10 min at room temperature, the λ_max_ of each sample within the 400–600 nm range was recorded using a UV-Vis spectrophotometer (Agilent, CA, USA). A characteristic red shift in λ_max_ for the polysaccharide-Congo red complexes compared to the blank under alkaline conditions indicates the formation of a triple-helix structure.

#### 4.3.7. Scanning Electron Microscopy Analysis

The surface morphology of the polysaccharide powders was observed using SEM (Sigma300, Zeiss, Oberkochen, Germany). Prior to imaging, the samples were passed through a 100-mesh sieve, mounted on conductive carbon tape, and sputter-coated with a thin layer of gold to enhance conductivity. The observations were then conducted at an accelerating voltage of 10 kV, with magnifications ranging from 100 to 4000×.

### 4.4. Regulation of NO Production in Macrophage by ***CDP1-5-1***, ***CDP2-2-2***, ***CDP2-3-2***, ***CTP1-5-1***, and ***CTP1-5-3***

RAW264.7 murine macrophages were maintained in DMEM containing 10% fetal bovine serum and 1% penicillin/streptomycin at 37 °C under a 5% CO_2_ atmosphere. For the CCK8 assay, cells were seeded in 96-well plates at a density of 1 × 10^5^ cells/mL and allowed to adhere. After reaching 90% confluence, they were treated with each glycan sample (**CDP1-5-1**, **CDP2-2-2**, **CDP2-3-2**, **CTP1-5-1**, and **CTP1-5-3**) at 1 mg/mL for 18 h. Subsequently, 10 μL of CCK8 solution was added to each well and incubated for 1 h, and the absorbance at 450 nm was measured to assess cell viability.

In parallel, NO production was quantified using the Griess reagent system (Beyotime Biotechnology). Cells were plated in 96-well plates at 1 × 10^6^ cells/mL and cultured for 24 h. They were then stimulated with the concentration as 1 mg/mL of the glycans for 18 h, using LPS (0.5 μg/mL) as a positive control. The accumulation of nitrite in the culture supernatant was determined according to the manufacturer’s instructions.

## 5. Conclusions

This study successfully demonstrates the feasibility of valorizing wine-making residues of CD and CT by isolating, purifying, and characterizing their polysaccharide components. Five homogeneous polysaccharide fractions, **CDP1-5-1**, **CDP2-2-2**, **CDP2-3-2** from CD, and **CTP1-5-1**, **CTP1-5-3** from CT, were obtained and structurally elucidated. These included an arabinan, two agavin-like fructans of different molecular weights, a heteropolysaccharide (arabinogalactan), and a galacturonan, respectively. Immunomodulatory evaluations revealed that all five polysaccharides significantly enhanced NO production in RAW264.7 macrophages, with the arabinan **CDP1-5-1** exhibiting the most potent activity. A preliminary SAR was established, highlighting arabinose as a key monosaccharide contributing to immunostimulatory effects. Furthermore, a methodological refinement for analyzing fructan-rich polysaccharides was introduced. This research not only provides fundamental data on the structure and immune-enhancing potential of polysaccharides from *Cistanche* residues but also lays a scientific foundation for their sustainable application in functional foods and pharmaceuticals, supporting the circular utilization of this valuable medicinal resource.

## Figures and Tables

**Table 1:** Molecular weight parameters of **CDP1-5-1**, **CDP2-2-2**, **CDP2-2-2**, **CTP1-5-1**, and **CTP1-5-3**.

Polysaccharides	Molecular Characteristics	Parameter	Detection Results
**CDP1-5-1**	Polydispersity	Mn Mp Mw Mz	766.2 kDa 773.5 kDa 852.9 kDa 1020.4 kDa
	Molar mass moments (g/mol)	Mw/Mn Mz/Mn	1.1 1.3
	Rms radius moments (nm)	Rn Rw	28.8 29.3
**CDP2-2-2**	Polydispersity	Mn Mp Mw Mz	29.7 kDa 27.5 kDa 39.2 kDa 119.2 kDa
	Molar mass moments (g/mol)	Mw/Mn Mz/Mn	1.3 4.0
	Rms radius moments (nm)	Rn Rw	8.1 9.2
**CDP2-3-2**	Polydispersity	Mn Mp Mw Mz	13.7 kDa 13.1 kDa 19.5 kDa 36.2 kDa
	Molar mass moments (g/mol)	Mw/Mn Mz/Mn	1.4 2.6
	Rms radius moments (nm)	Rn Rw	11.9 11.3
**CTP1-5-1**	Polydispersity	Mn Mp Mw Mz	640.7 kDa 754.7 kDa 797.1 kDa 1044.7 kDa
	Molar mass moments (g/mol)	Mw/Mn Mz/Mn	1.2 1.6
	Rms radius moments (nm)	Rn Rw	24.9 26.1
**CTP1-5-3**	Polydispersity	Mn Mp Mw Mz	15.9 kDa 13.1 kDa 20.7 kDa 39.6 kDa
	Molar mass moments (g/mol)	Mw/Mn Mz/Mn	1.3 4.0
	Rms radius moments (nm)	Rn Rw	43.1 43.9

Mn: number-averaged molecular weight; Mp: peak molecular weight; Mw: weight-averaged molecular weight; Mz: z-averaged molecular weight; Mw/Mn and Mz/Mn: polydispersity or polydispersity index, which is an index of the breadth of the molecular weight distribution; R (Rn, Rw): root-mean-square (RMS) radius describing the mass distribution around the center of gravity.

**Table 2:** The methylation results of **CDP1-5-1**, **CDP2-2-2**, **CDP2-2-2**, **CTP1-5-1**, and **CTP1-5-3**.

Polysaccharides	Linkage Type	Methylated Sugars	Molar Ratio (%)	*t*_R_ (min)	Mass Fragments (*m*/*z*)
**CDP1-5-1**	t-L-Ara*f*	1,4-Di-*O*-acetyl-1-deuterio-2,3,5-tri-*O*-methyl-D-arabinitol	1.0	25.8	59, 71, 87, 102, 118, 129, 145, 161, 162
	→ 5)-L-Ara*f*-(1 →	1,4,5-Tri-*O*-acetyl-1-deuterio-2,3-di-*O*-methyl-D-arabinitol	1.2	29.3	59, 80, 102, 118, 129, 162, 189
	→ 3,5)-L-Ara*f*-(1 →	1,3,4,5-Tetra-*O*-acetyl-1-deuterio-2-*O*-methyl-D-arabinitol	1.3	31.1	59, 73, 99, 118, 127, 159, 201, 261
**CDP2-2-2**	D-Fru*f*-(2 →	2,5-Di-*O*-acetyl-2-deuterio-1,3,4,6-tetra-*O*-methyl-D-mannitol	–	28.8	71, 87,101, 129, 145, 161, 162, 186, 205
	→ 6)-D-Fru*f*-(2 →	2,5,6-Tri-*O*-acetyl-2-deuterio-1,3,4-tri-*O*-methyl-D-mannitol	–	32.2	57, 87, 129, 146, 162, 173, 189, 206
	→ 1)-D-Fru*f*-(2 →	1,2,5-Tri-*O*-acetyl-2-deuterio-3,4,6-tri-*O*-methyl-D-mannitol	–	32.3	57, 87, 118, 129, 146, 161, 189, 203
	→ 1,6)-D-Fru*f*-(2 →	1,2,5,6-Tetra-*O*-acetyl-2-deuterio-3,4-di-*O*-methyl-D-mannitol	–	34.5	60, 73, 87, 99, 115, 129, 143, 157, 171, 191, 199
**CDP2-3-2**	D-Fru*f*-(2 →	2,5-Di-*O*-acetyl-2-deuterio-1,3,4,6-tetra-*O*-methyl-D-mannitol	–	28.8	71, 87,101, 129, 145, 161, 162, 186, 205
	→ 6)-D-Fru*f*-(2 →	2,5,6-Tri-*O*-acetyl-2-deuterio-1,3,4-tri-*O*-methyl-D-mannitol	–	32.2	57, 87, 129, 146, 162, 173, 189, 206
	→ 1)-D-Fru*f*-(2 →	1,2,5-Tri-*O*-acetyl-2-deuterio-3,4,6-tri-*O*-methyl-D-mannitol	–	32.3	57, 87, 118, 129, 146, 161, 189, 203
	→ 1,6)-D-Fru*f*-(2 →	1,2,5,6-Tetra-*O*-acetyl-2-deuterio-3,4-di-*O*-methyl-D-mannitol	–	34.5	60, 73, 87, 99, 115, 129, 143, 157, 191, 199
**CTP1-5-1**	t-L-Ara*f*	1,4-Di-*O*-acetyl-1-deuterio-2,3,5-tri-*O*-methyl-D-arabinitol	2.0	25.8	59, 71, 87, 102, 118, 129, 145, 161, 162
	→ 5)-L-Ara*f*-(1 →	1,4,5-Tri-*O*-acetyl-1-deuterio-2,3-di-*O*-methyl-D-arabinitol	2.1	29.3	59, 80, 102, 118, 129, 162, 189
	→ 3,5)-L-Ara*f*-(1 →	1,3,4,5-Tetra-*O*-acetyl-1-deuterio-2-*O*-methyl-D-arabinitol	2.0	31.1	59, 73, 99, 118, 127, 159, 201, 261
	→ 4)-D-Gal*p*-(1 →	1,4,5-Tri-*O*-acetyl-1-deuterio-2,3,6-tri-*O*-methyl-D-galactitol	7.9	32.4	71, 87, 102, 118, 131, 142, 173, 203, 233
**CTP1-5-3**	→ 4)-D-Gal*p*-(1 →	1,4,5-Tri-*O*-acetyl-1-deuterio-2,3,6-tri-*O*-methyl-D-galactitol	–	32.8	71, 87, 102, 118, 131, 142, 173, 203, 233

**Table 3:** ^1^H and ^13^C NMR data of **CDP1-5-1** (500 MHz, D_2_O).

Sugar Residues	Chemical Shifts (δ ppm)				
	H-1	H-2	H-3	H-4	H-5
	C-1	C-2	C-3	C-4	C-5
→ 5)-α-L-Ara*f*-(1 → **a**	5.09	4.12	4.03	4.22	3.82
	107.9	81.3	77.0	82.8	66.7
→ 3,5)-α-L-Ara*f*-(1 → **b**	5.11	4.29	4.10	4.30	3.95/3.89
	107.9	79.6/79.7	82.7	82.1	66.3/67.0
α-L-Ara*f*-(1 → **c**	5.15	4.13	3.95	4.03	3.73, 3.84
	107.5	81.7	77.0	84.4	61.6
α-L-Ara*f*-(1 → **d**	5.15	4.13	3.95	4.03	3.73, 3.84
	107.6	81.7	77.0	84.3	61.6

**Table 4:** ^1^H and ^13^C NMR data of **CDP2-2-2** (500 MHz, D_2_O).

Sugar Residues	Chemical Shifts (δ ppm)					
	H-1	H-2	H-3	H-4	H-5	H-6
	C-1	C-2	C-3	C-4	C-5	C-6
→ 6)-β-D-Fru*f*-(2 → **a**	3.70, 3.77	—	4.20	4.11	3.97	3.59, 3.90
	60.4	104.5/104.6	76.8	75.7	80.7	63.8
→ 1,6)-β-D-Fru*f*-(2 → **b**	3.77, 3.87	—	4.21	4.10	3.97	3.57, 3.95
	60.9	104.1	77.0	75.4	80.7	63.6
→ 1)-β-D-Fru*f*-(2 → **c**	3.76, 3.86	—	4.06	4.15	4.21	3.72, 3.80
	61.5	104.2/104.4	77.2/77.3	74.7/75.1	81.4/81.5/81.6	62.9/63.0
β-D-Fru*f*-(2 → **d**	3.77, 3.80	—	4.21	4.10	3.92	3.77, 3.87
	60.9	103.9	77.8	75.6	81.5	62.6

**Table 5:** ^1^H and ^13^C NMR data of **CDP2-3-2** (500 MHz, D_2_O).

Sugar Residues	Chemical Shifts (δ ppm)					
	H-1	H-2	H-3	H-4	H-5	H-6
	C-1	C-2	C-3	C-4	C-5	C-6
→ 6)-β-D-Fru*f*-(2 → **a**	3.69, 3.77	—	4.19	4.10	3.96	3.58, 3.90
	60.4	104.5/104.6/104.7	76.8	75.8	80.7	63.8
→ 1,6)-β-D-Fru*f*-(2 → **b**	3.77, 3.87	—	4.20	4.10	3.96	3.56, 3.94
	60.5	104.1	77.0	75.5	80.7	63.7
→ 1)-β-D-Fru*f*-(2 → **c**	3.75, 3.85	—	4.05	4.12	4.19	3.71, 3.81
	61.5	104.2/104.3/104.4	77.0/77.2	74.7/75.1	81.3/81.5/81.6	62.9
β-D-Fru*f*-(2 → **d**	3.73, 3.84	—	4.19	4.10	3.90	3.54, 3.89
	60.9	103.9	77.8	75.7	81.6	62.6

**Table 6:** ^1^H and ^13^C NMR data of **CTP1-5-1** (500 MHz, D_2_O).

Sugar Residues	Chemical Shifts (δ ppm)					
	H-1	H-2	H-3	H-4	H-5	H-6
	C-1	C-2	C-3	C-4	C-5	C-6
→ 5)-α-L-Ara*f*-(1 → **a**	5.10	4.15	4.05	4.23	3.83	
	107.9	81.3	77.0	82.9	66.7	
→ 3,5)-α-L-Ara*f*-(1 → **b**	5.13	4.30	4.11	4.31	3.96/3.88	
	107.9	79.6/79.7	82.9	82.6	66.3/67.0	
α-L-Ara*f*-(1 → **c**	5.16	4.15	3.96	4.05	3.74, 3.85	
	107.6	81.7	77.0	84.3	61.6	
α-L-Ara*f*-(1 → **d**	5.16	4.15	3.96	4.05	3.74, 3.85	
	107.6	81.7	77.0	84.3	61.6	
→ 4)-β-D-Gal*p*-(1 → **e**	4.66	3.69	3.78	4.19	3.73	3.71, 3.83
	104.8	72.3	73.8	78.1	75.0	61.2

**Table 7:** ^1^H and ^13^C NMR data of **CTP1-5-3** (500 MHz, D_2_O).

Sugar Residues	Chemical Shifts (δ ppm)					
	H-1	H-2	H-3	H-4	H-5	H-6
	C-1	C-2	C-3	C-4	C-5	C-6
→ 4)-α-D-Gal*p*A-(1 →	5.06	3.77	3.99	4.41	4.75	—
	99.4	68.6	69.3	78.3	71.8	175.9

## Data Availability

Data will be available upon request.

## Supplementary Materials

The following supporting information can be downloaded at: https://www.mdpi.com/article/10.3390/molecules30244754/s1, Data on the FT-IR spectra, molecular weight distribution, molecular conformation, GC-MS analysis, SEM images, Congo red assay, and in vitro activity assay for **CDP1-5-1**, **CDP2-2-2**, **CDP2-3-2**, **CTP1-5-1**, and **CTP1-5-3** are provided in the Supporting Information.

## Abbreviations

The following abbreviations are used in this manuscript:

Ara	Arabinose
CD	Residue of *C. deserticola* following 52% alcohol pretreatment
CDP	Crude polysaccharides of *C. deserticola*
CT	Residue of *C. tubulosa* following 52% alcohol pretreatment
CTP	Crude polysaccharides of *C. Tubulosa*
DMSO	Dimethyl sulfoxide
Fru	Fructose
FT-IR	Fourier-transform infrared
Gal	Galactose
GalA	Galacturonic acid
GC-MS	Gas chromatography–mass spectrometry
Glc	Glucose
GlcA	Glucuronic acid
HPAEC-MALLS-RID	High-performance anion-exchange chromatography coupled with multi-angle laser light scattering and refractive index detection
HPLC	High-performance liquid chromatography
IF	Immunofluorescence
Man	Mannose
Mn	Number-average molecular weight
Mw	Weight-average molecular weight
NMR	nuclear magnetic resonance
NO	Nitric oxide
PDI	Polydispersity index
PMAA	Partially methylated alditol acetate
PMP	1-Phenyl-3-methyl-5-pyrazolone
Rha	Rhamnose
RMS	Root mean square
SAR	Structure–activity relationship
SEM	Scanning electron microscope
TCM	Traditional Chinese medicine
TFA	Trifluoroacetic acid
TNF-α	Tumor necrosis factor α
Xyl	Xylose
