# Correction: Ruan et al. Comparison of Extraction, Isolation, Purification, Structural Characterization and Immunomodulatory Activity of Polysaccharides from Two Species of *Cistanche*. *Molecules* 2025, *30*, 4754

**DOI:** 10.3390/molecules31122112

**Published:** 2026-06-16

**Authors:** Jingya Ruan, Juan Zhang, Lequan Yu, Ping Zhang, Anxin Chen, Dongmei Wang, Yi Zhang, Tao Wang

**Affiliations:** 1 Tianjin Key Laboratory of TCM Chemistry and Analysis, Tianjin University of Traditional Chinese Medicine, 10 Poyanghu Road, West Area, Tuanbo New Town, Jinghai District, Tianjin 301617, China; ruanjingya@tjutcm.edu.cn (J.R.); 18822575726@163.com (L.Y.); zp10259611@163.com (P.Z.); 2 Xinjiang Institute of Materia Medica, 18 Zhengyang Road, High-Tech Industrial Development Zone (Xinshi District), Urumqi 830017, China; ezhangjuane76@sina.com; 3 Xinjiang LifeCore High-Tech Co., Ltd., 55 Dongrong Road, Urumqi High-Tech Industrial Development Zone (Xinshi District), Urumqi 830017, China; cnguorongchen@sina.com (A.C.); smhl57624231@sina.com (D.W.)

## Error in Figures

In the original publication [1], there were mistakes in the α-L-Araf unit of Figures 2I, 3A,D and 6I as published. According to the Haworth projection rules for pentose aldofuranose, the D/L configuration is determined by the orientation of the C-4 substituent (up for D, down for L), and the α/β anomeric configuration is defined by the relative orientation of the anomeric C-1 hydroxyl group to the C-4 substituent (same orientation for β, opposite for α). The corrected Figure 2, Figure 3 and Figure 6 appear below.

The authors state that the scientific conclusions are unaffected. This correction was approved by the Academic Editor. The original publication has also been updated.

## Figures and Tables

**Figure 2:**
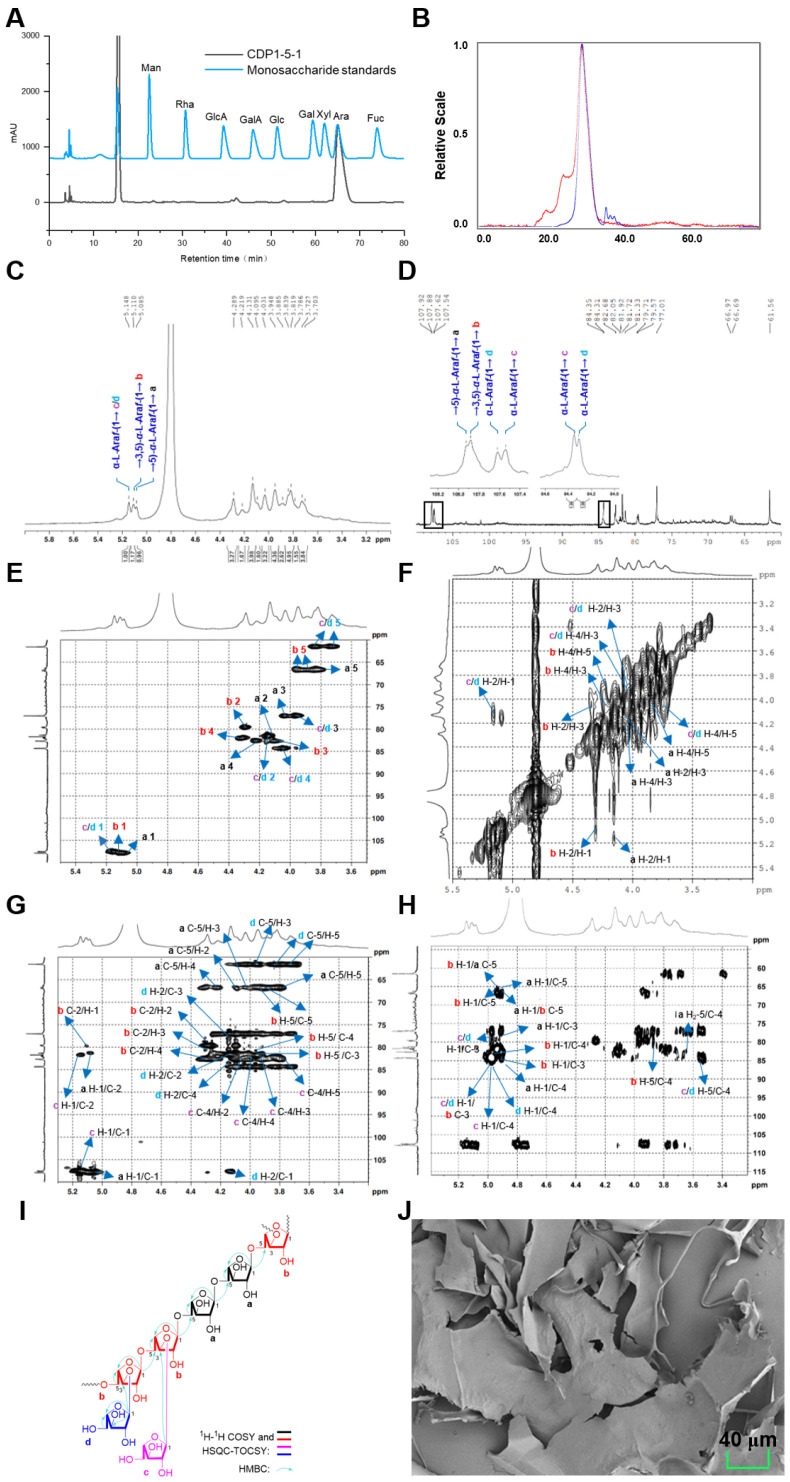
Structural characterization of **CDP1-5-1**. (**A**) Monosaccharide composition analysis; (**B**) HPGPC-MALLS-RID spectrum (Red line: signal detected by LS detector; Blue line: signal detected by RID detector); (**C**) ^1^H NMR spectrum; (**D**) ^13^C NMR spectrum; (**E**) HSQC spectrum; (**F**) ^1^H-^1^H COSY spectrum; (**G**) HSQC-TOCSY spectrum; (**H**) HMBC spectrum; (**I**) main 2D NMR correlations; (**J**) SEM images (500-times magnification).

**Figure 3:**
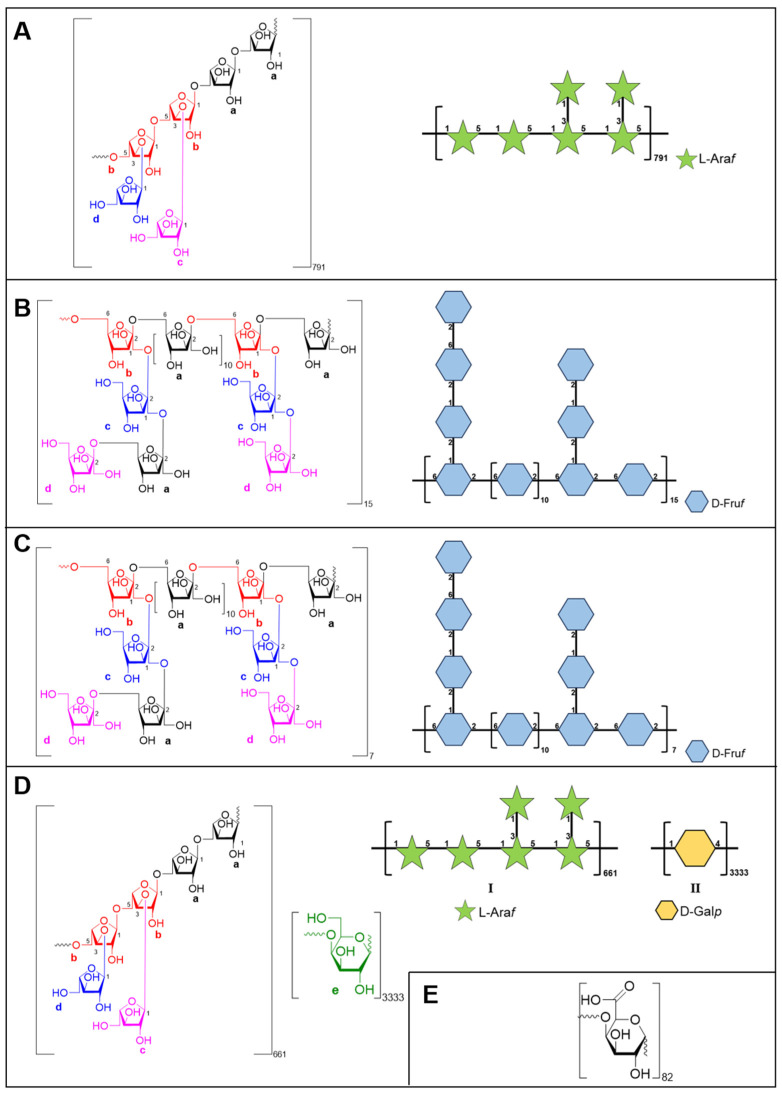
Structures of **CDP1-5-1** (**A**), **CDP2-2-2** (**B**), **CDP2-3-2** (**C**), **CTP1-5-1** (**D**), and **CTP1-5-3** (**E**).

**Figure 6:**
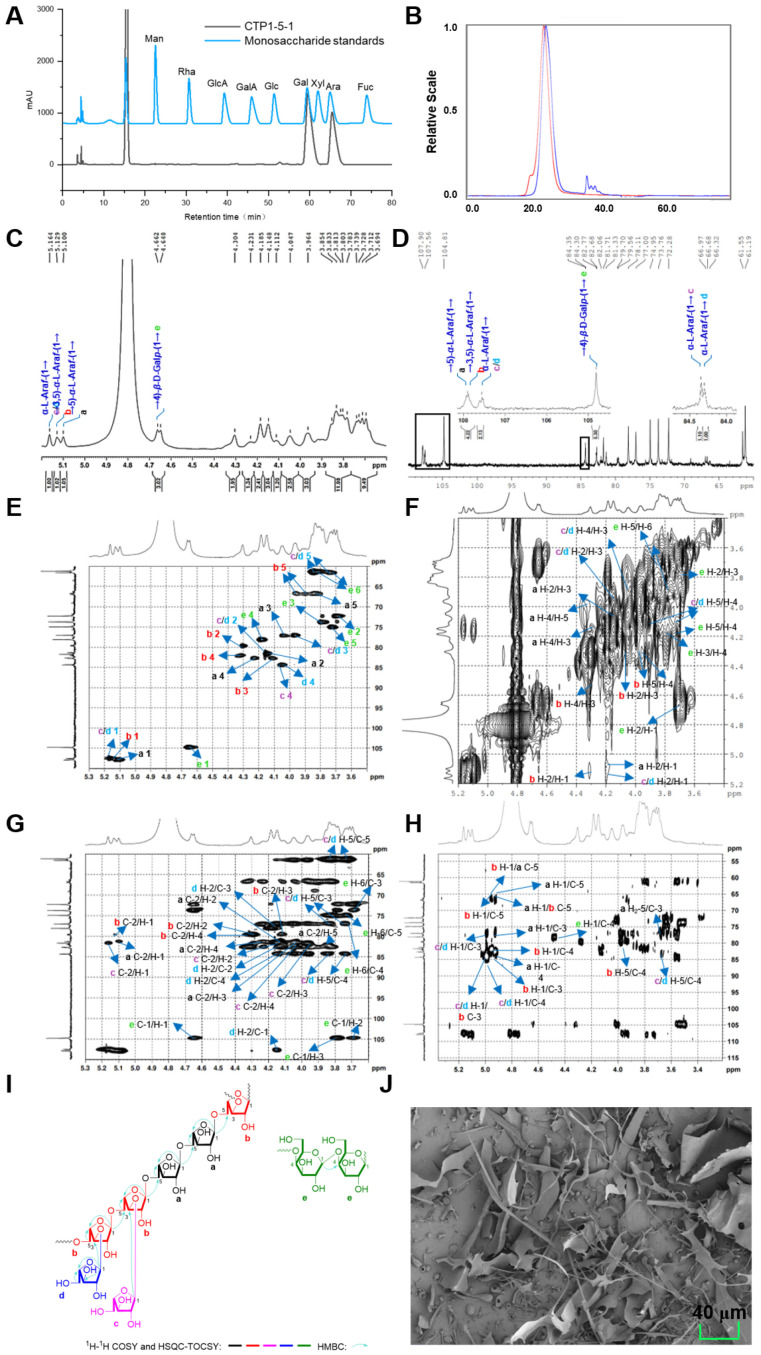
Structural characterization of **CTP1-5-1**. (**A**) Monosaccharide composition analysis; (**B**) HPGPC-MALLS-RID spectrum (Red line: signal detected by LS detector; Blue line: signal detected by RID detector); (**C**) ^1^H NMR spectrum; (**D**) ^13^C NMR spectrum; (**E**) HSQC spectrum; (**F**) ^1^H-^1^H COSY spectrum; (**G**) HSQC-TOCSY spectrum; (**H**) HMBC spectrum; (**I**) main 2D NMR correlations; (**J**) SEM images (500-times magnification) (40 μm).
