# Supplementary material for: Comparison of Extraction, Isolation, Purification, Structural Characterization and Immunomodulatory Activity of Polysaccharides from Two Species of Cistanche
Source: Molecules. 2025 Dec 12;30(24):4754. doi: 10.3390/molecules30244754 (PMC12736087; doi:10.3390/molecules30244754)
Supplement: Supplementary file 1 [file molecules-30-04754-s001.zip › molecules-3980964-supplementary.pdf]

Supporting information

Comparison of extraction, isolation, purification, structural characterization and immunomodulatory activity of polysaccharides from two species of *Cistanche*

Jingya Ruan[1,#], Juan Zhang[2,#], Lequan Yu[1], Ping Zhang[1], Anxin Chen[3], Dongmei Wang[3], Yi Zhang[1]* and Tao Wang[1]*

[1] Tianjin Key Laboratory of TCM Chemistry and Analysis, Tianjin University of Traditional Chinese Medicine, 10 Poyanghu Road, West Area, Tuanbo New Town, Jinghai District, 301617, Tianjin, China; ruanjingya@tjutcm.edu.cn (J.-Y.R.); 18822575726@163.com (L.-Q.Y.); zp10259611@163.com (P.Z.);

[2] Xinjiang Institute of Materia Medica, 18 Zhengyang Road, High-tech Industrial Development Zone (Xinshi District), Urumqi City, Xinjiang Uygur Autonomous Region, 830017, China; ezhangjuane76@sina.com (J.Z.);

[3] Xinjiang LifeCore High-Tech Co., Ltd., 55 Dongrong Road, Urumqi High-tech Industrial Development Zone (Xinshi District), Urumqi City, Xinjiang Uygur Autonomous Region, China; 2228423280@qq.com (A.-X.C.); 372627151@qq.com (D.-M.W.);

[#] These authors contributed equally;

* Correspondence: zhwwxzh@tjutcm.edu.cn (Y.Z.); wangtao@tjutcm.edu.cn (T.W.); Tel./Fax: +86-22-5959-6168 (T.W.)

Figure S1 FT-IR spectrum of **CDP1-5-1** 4
Figure S2 Molecular weight distribution plot of **CDP1-5-1** 5
Figure S3 Molecular conformation analysis of **CDP1-5-1** 6
Figure S4 The FT-IR spectra before and after methylation reaction and GC-MS analysis results of **CDP1-5-1** 7
Figure S5 ^{1}H NMR spectrum of **CDP1-5-1** (500 MHz, D_2O) 8
Figure S6 ^{13}C NMR spectrum of **CDP1-5-1** (125 MHz, D_2O) 8
Figure S7 HSQC (D_2O) spectrum of **CDP1-5-1** 9
Figure S8 ^{1}H ^{1}H COSY (D_2O) spectrum of **CDP1-5-1** 9
Figure S9 HSQC-TOCSY (D_2O) spectrum of **CDP1-5-1** 10
Figure S10 HMBC (D_2O) spectrum of **CDP1-5-1** 10
Figure S11 SEM images of **CDP1-5-1** (10000×) 11
Figure S12 Congo red assay of **CDP1-5-1** 12
Figure S13 FT-IR spectrum of **CDP2-2-2** 13
Figure S14 Molecular weight distribution plot of **CDP2-2-2** 14
Figure S15 Molecular conformation analysis of **CDP2-2-2** 15
Figure S16 ^{1}H NMR spectrum of **CDP2-2-2** (500 MHz, D_2O) 16
Figure S17 ^{13}C NMR spectrum of **CDP2-2-2** (125 MHz, D_2O) 16
Figure S18 HSQC (D_2O) spectrum of **CDP2-2-2** 17
Figure S19 ^{1}H ^{1}H COSY (D_2O) spectrum of **CDP2-2-2** 17
Figure S20 HSQC-TOCSY (D_2O) spectrum of **CDP2-2-2** 18
Figure S21 HMBC (D_2O) spectrum of **CDP2-2-2** 18
Figure S22 The FT-IR spectra before and after methylation reaction and GC-MS analysis results of **CDP2-2-2** 19
Figure S23 SEM images of **CDP2-2-2** (10000×) 20
Figure S24 Congo red assay of **CDP2-2-2** 21
Figure S25 FT-IR spectrum of **CDP2-3-2** 22
Figure S26 Molecular weight distribution plot of **CDP2-3-2** 23
Figure S27 Molecular conformation analysis of **CDP2-3-2** 24
Figure S28 ^{1}H NMR spectrum of **CDP2-3-2** (500 MHz, D_2O) 25
Figure S29 ^{13}C NMR spectrum of **CDP2-3-2** (125 MHz, D_2O) 25
Figure S30 HSQC (D_2O) spectrum of **CDP2-3-2** 26
Figure S31 ^{1}H ^{1}H COSY (D_2O) spectrum of **CDP2-3-2** 26
Figure S32 HSQC-TOCSY (D_2O) spectrum of **CDP2-3-2** 27
Figure S33 HMBC (D_2O) spectrum of **CDP2-3-2** 27
Figure S34 The FT-IR spectra before and after methylation reaction and GC-MS analysis results of **CDP2-3-2** 28
Figure S35 SEM images of **CDP2-3-2** (10000×) 29
Figure S36 Congo red assay of **CDP2-3-2** 30
Figure S37 FT-IR spectrum of **CTP1-5-1** 31
Figure S38 Molecular weight distribution plot of **CTP1-5-1** 32
Figure S39 Molecular conformation analysis of **CTP1-5-1** 33
Figure S40 The FT-IR spectra before and after methylation reaction and GC-MS analysis results of **CTP1-5-1** 34

Figure S41 ^{1}H NMR spectrum of **CTP1-5-1** (500 MHz, D_2O) 35
Figure S42 ^{13}C NMR spectrum of **CTP1-5-1** (125 MHz, D_2O) 35
Figure S43 HSQC (D_2O) spectrum of **CTP1-5-1** 36
Figure S44 ^{1}H ^{1}H COSY (D_2O) spectrum of **CTP1-5-1** 36
Figure S45 HSQC-TOCSY (D_2O) spectrum of **CTP1-5-1** 37
Figure S46 HMBC (D_2O) spectrum of **CTP1-5-1** 37
Figure S47 SEM images of **CTP1-5-1** (10000×) 38
Figure S48 Congo red assay of **CTP1-5-1** 39
Figure S49 FT-IR spectrum of **CTP1-5-3** 40
Figure S50 Molecular weight distribution plot of **CTP1-5-3** 41
Figure S51 Molecular conformation analysis of **CTP1-5-3** 42
Figure S52 The FT-IR spectra before and after methylation reaction and GC-MS analysis results of **CTP1-5-3** 43
Figure S53 ^{1}H NMR spectrum of **CTP1-5-3** (500 MHz, D_2O) 44
Figure S54 ^{13}C NMR spectrum of **CTP1-5-3** (125 MHz, D_2O) 44
Figure S55 HSQC (D_2O) spectrum of **CTP1-5-3** 45
Figure S56 ^{1}H ^{1}H COSY (D_2O) spectrum of **CTP1-5-3** 45
Figure S57 HSQC-TOCSY (D_2O) spectrum of **CTP1-5-3** 46
Figure S58 HMBC (D_2O) spectrum of **CTP1-5-3** 46
Figure S59 SEM images of **CTP1-5-3** (10000×) 47
Figure S60 Congo red assay of **CTP1-5-3** 48
Table S1 Effects of **CDP1-5-1**, **CDP2-2-2**, **CDP2-3-2**, **CTP1-5-1**, and **CTP1-5-3** on cell viability and NO production of RAW264.7 cell 49

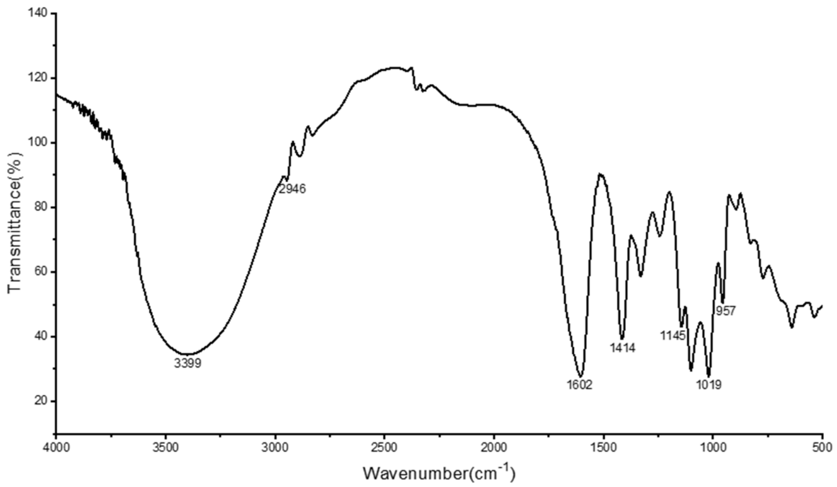

Figure S1 FT-IR spectrum of **CDP1-5-1**

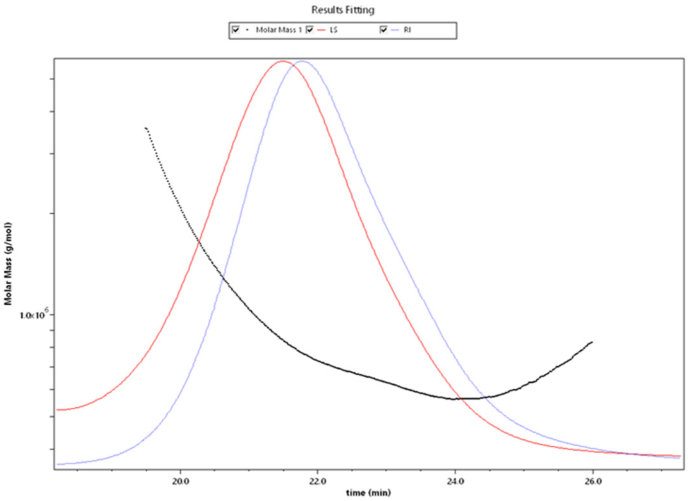

Figure S2 Molecular weight distribution plot of **CDP1-5-1**

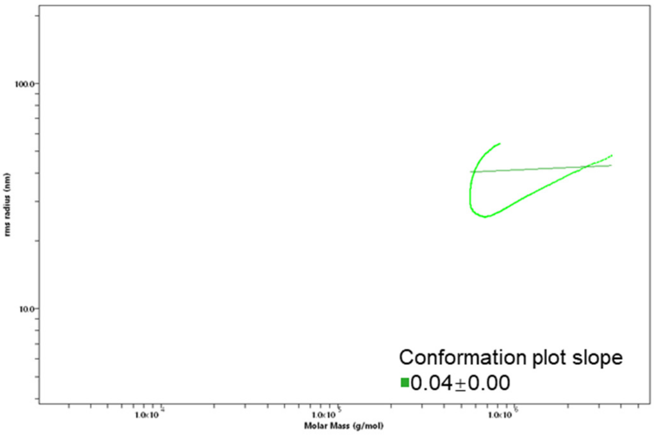

Figure S3 Molecular conformation analysis of **CDP1-5-1**

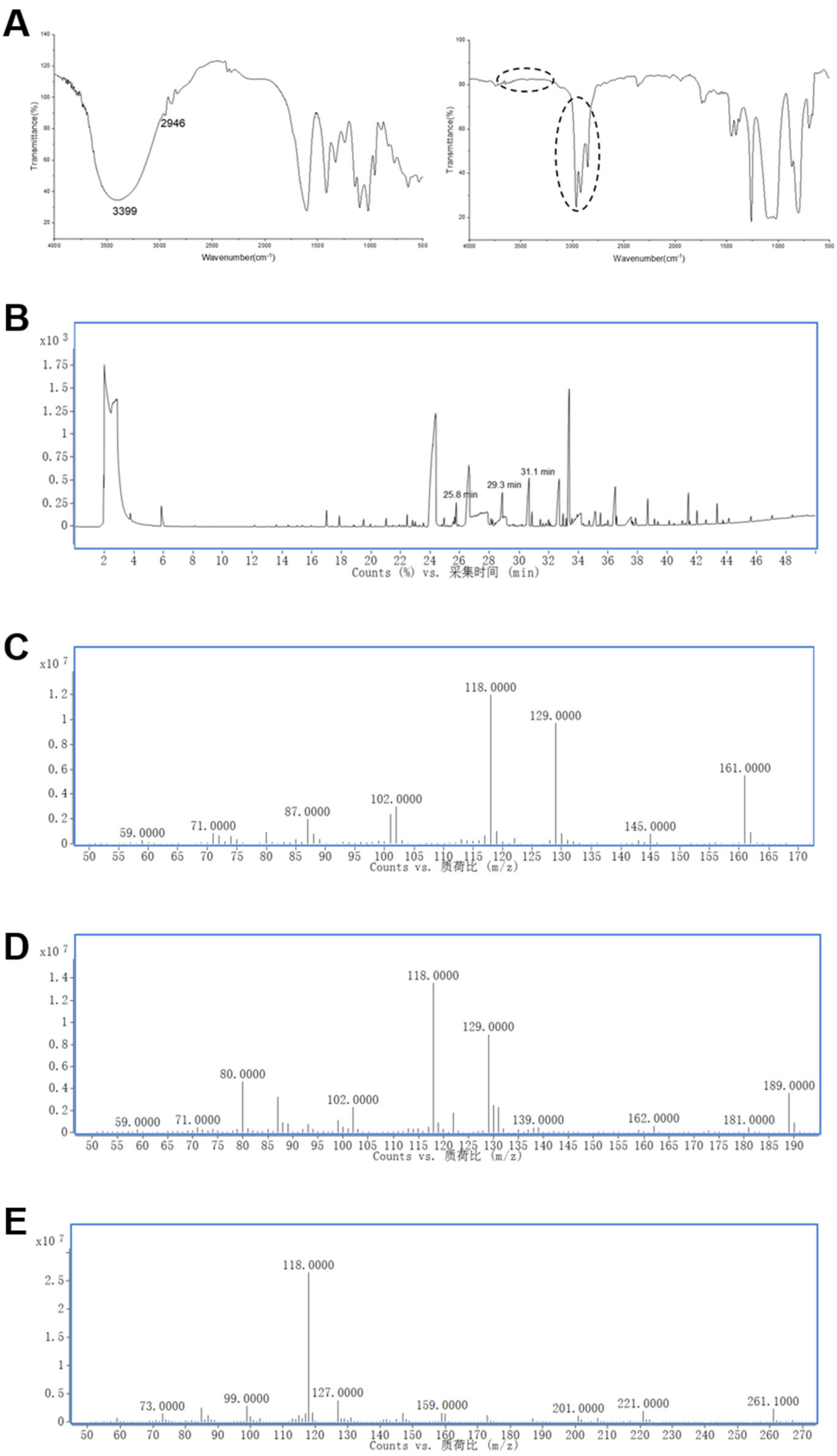

Figure S4 The FT-IR spectra before and after methylation reaction and GC-MS analysis results of **CDP1-5-1**

A: FT-IR spectra of **CDP1-5-1** before and after methylation; B: TIC of **CDP1-5-1**; C: spectrum of t-L-Ara*f* (25.8 min); D: spectrum of →5)-L-Ara*f*-(1→ (29.3 min); E: spectrum of →3,5)-L-Ara*f*-(1→ (31.1 min)

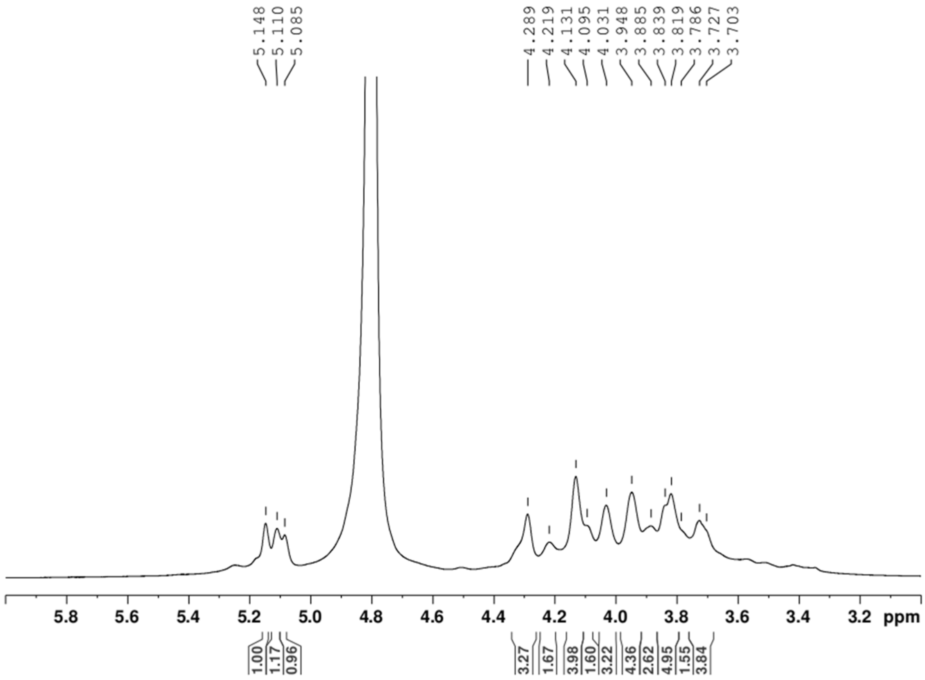

Figure S5 ¹H NMR spectrum of **CDP1-5-1** (500 MHz, D_2O)

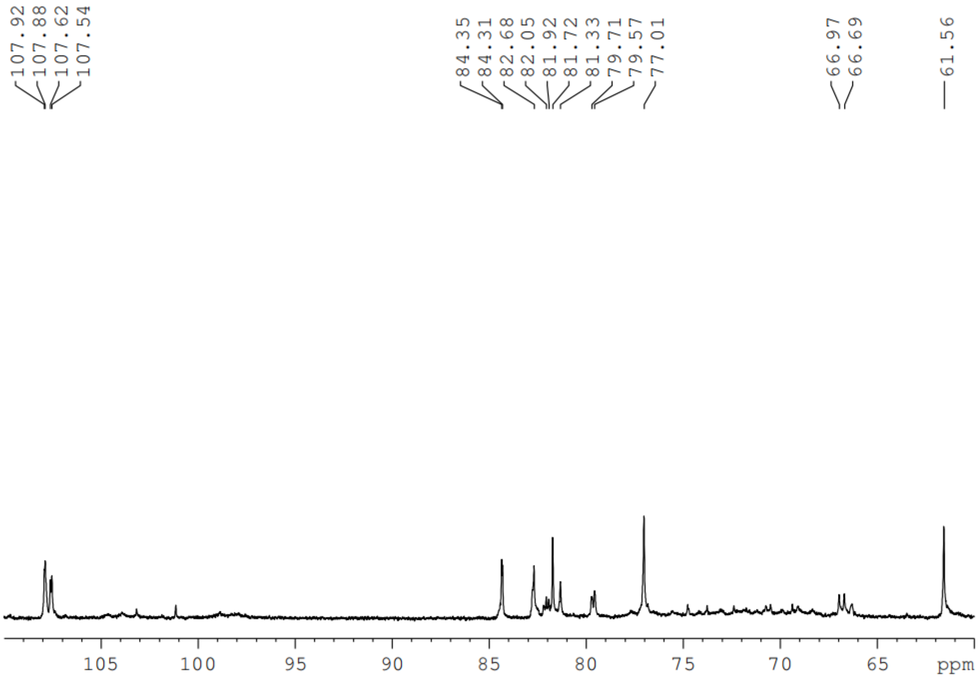

Figure S6 ¹³C NMR spectrum of **CDP1-5-1** (125 MHz, D_2O)

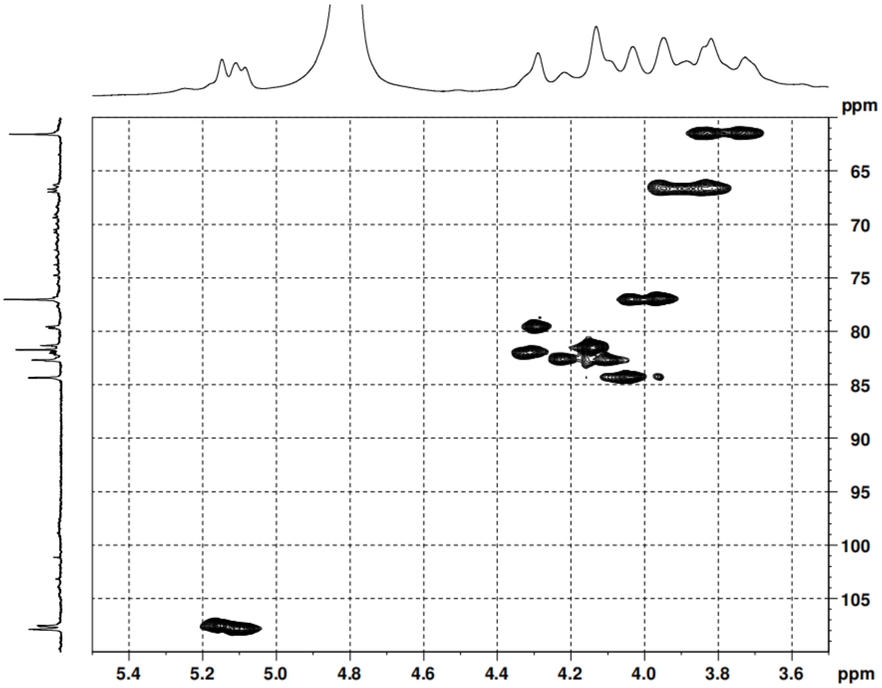

Figure S7 HSQC (D_2O) spectrum of **CDP1-5-1**

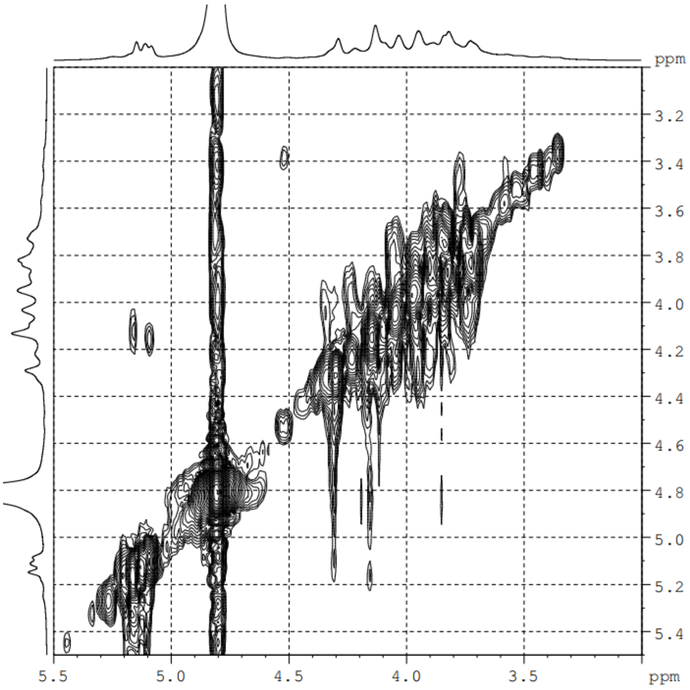

Figure S8 ^1H ^1H COSY (D_2O) spectrum of **CDP1-5-1**

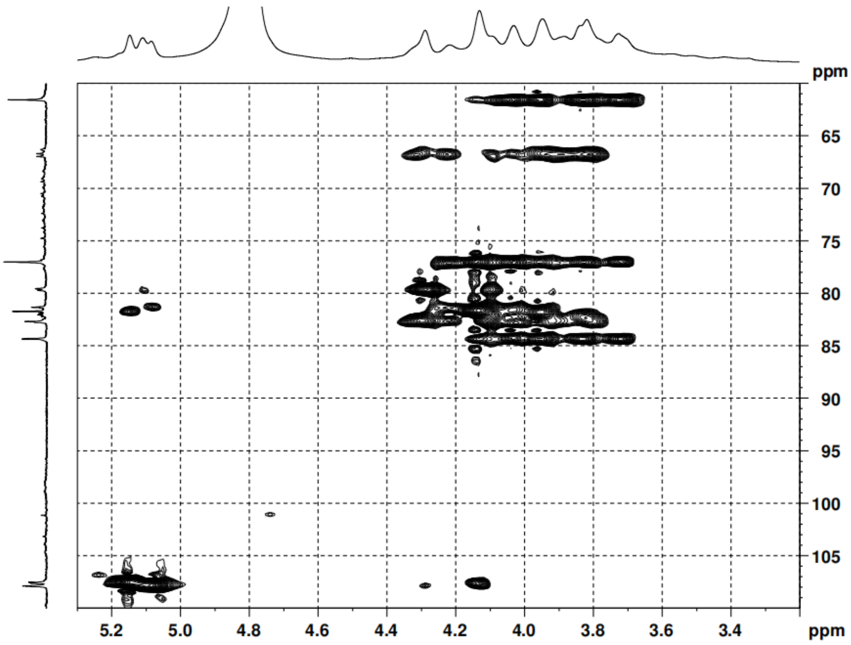

Figure S9 HSQC-TOCSY (D_2O) spectrum of **CDP1-5-1**

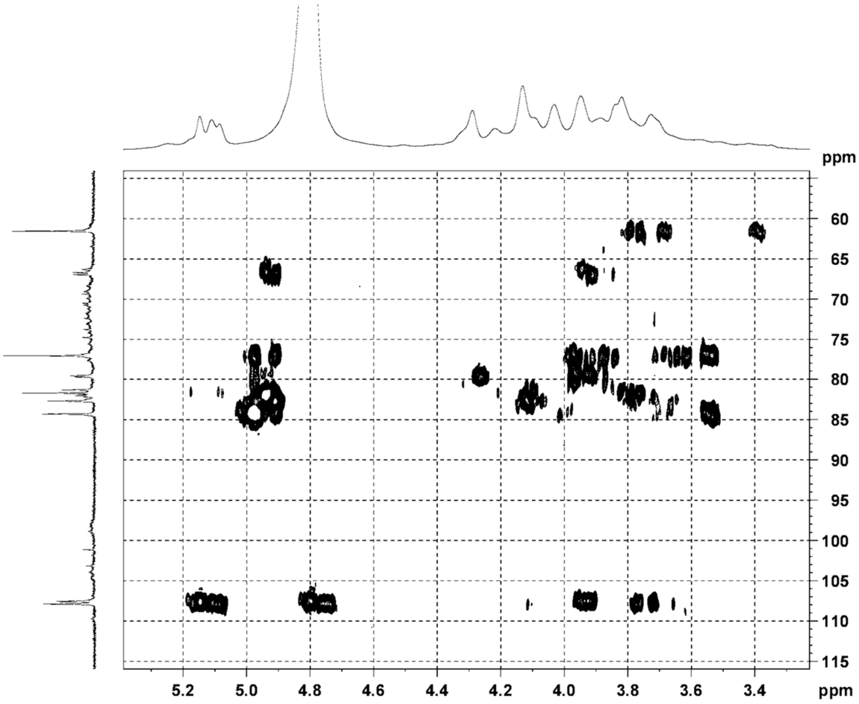

Figure S10 HMBC (D_2O) spectrum of **CDP1-5-1**

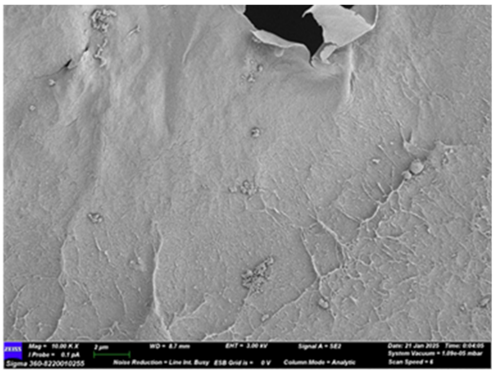

Figure S11 SEM images of **CDP1-5-1** (10000×)

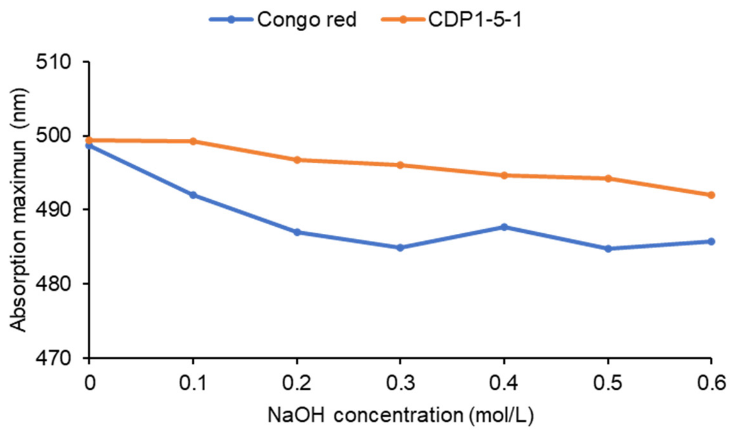

Figure S12 Congo red assay of **CDP1-5-1**

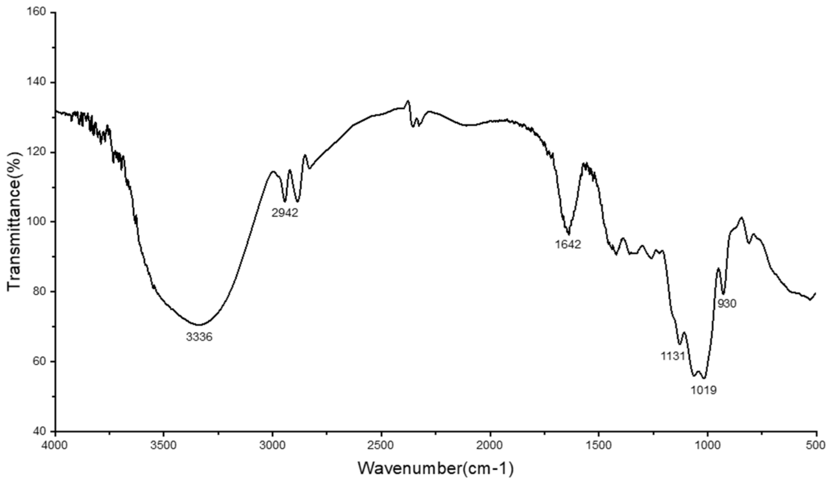

Figure S13 FT-IR spectrum of **CDP2-2-2**

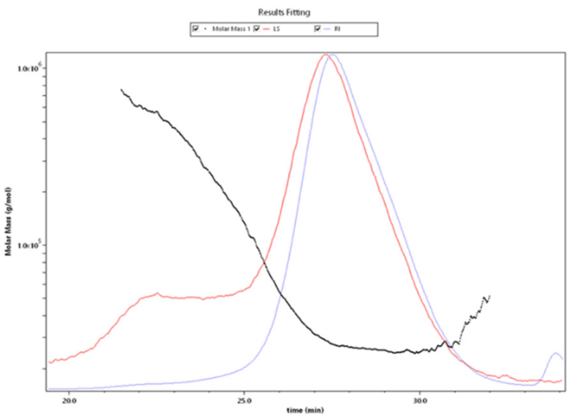

Figure S14 Molecular weight distribution plot of **CDP2-2-2**

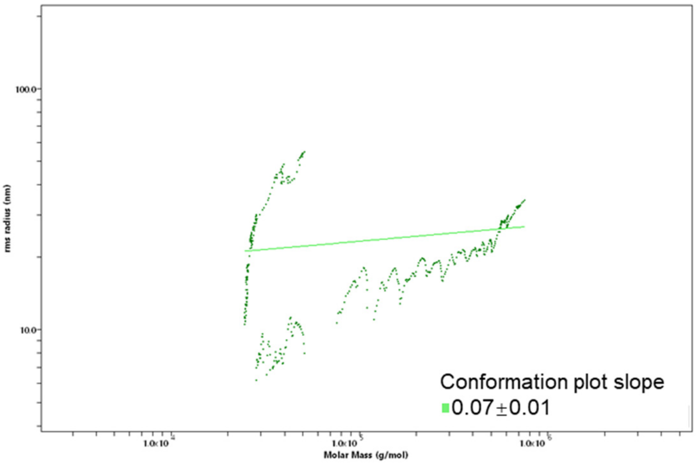

Figure S15 Molecular conformation analysis of **CDP2-2-2**

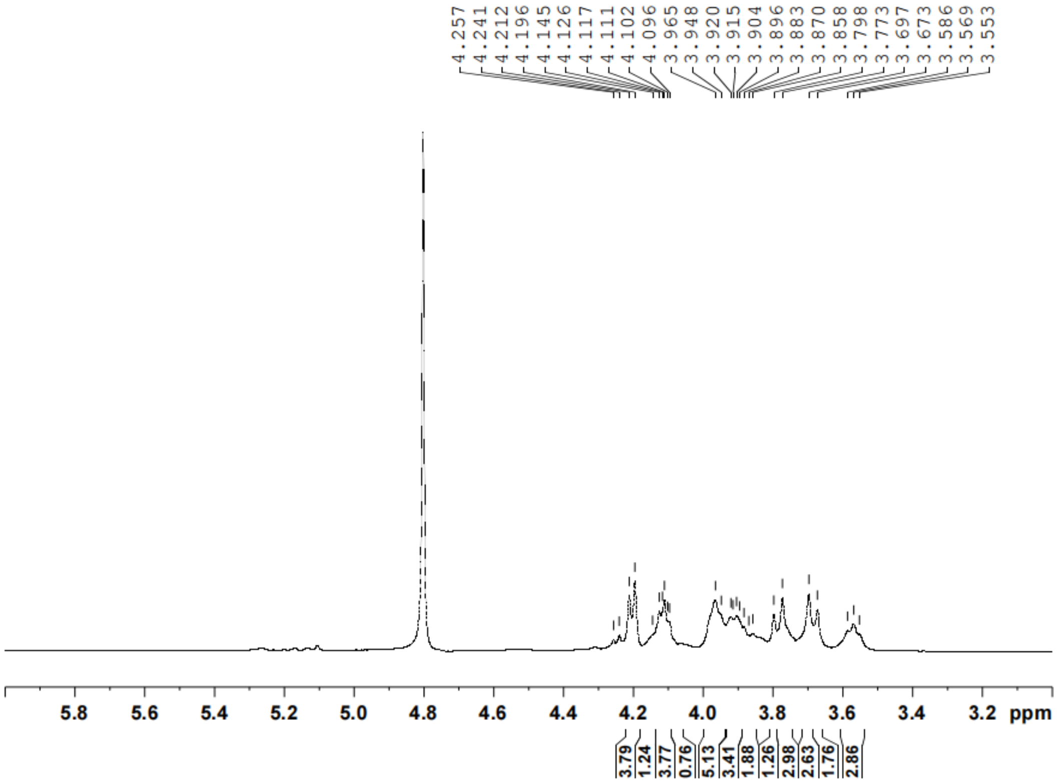

Figure S16 ^{1}H NMR spectrum of **CDP2-2-2** (500 MHz, D_2O)

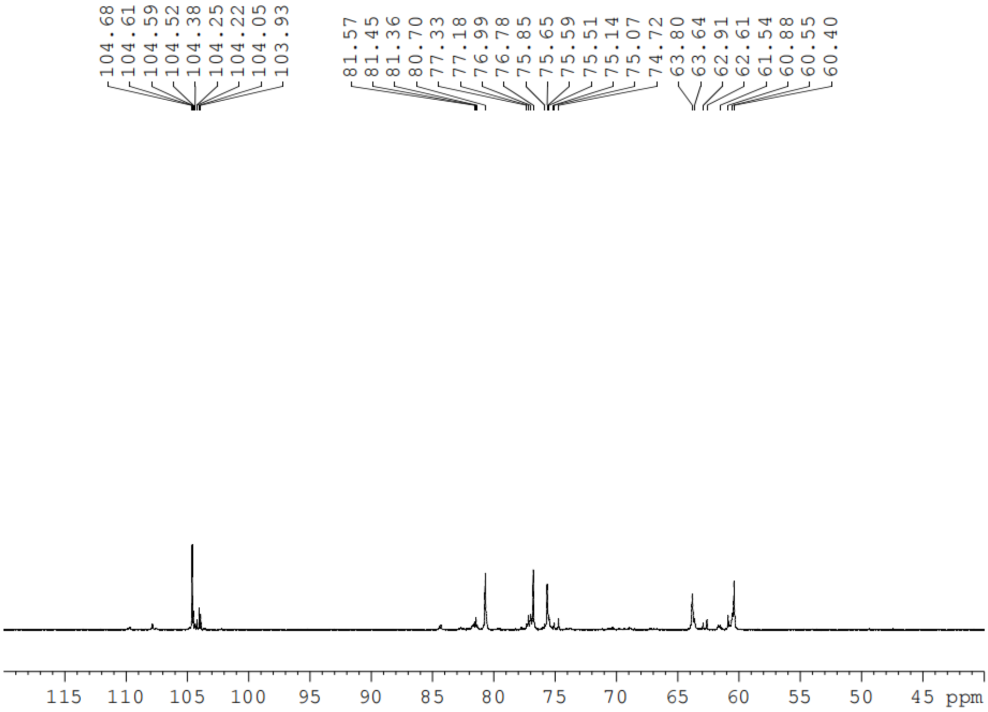

Figure S17 ^{13}C NMR spectrum of **CDP2-2-2** (125 MHz, D_2O)

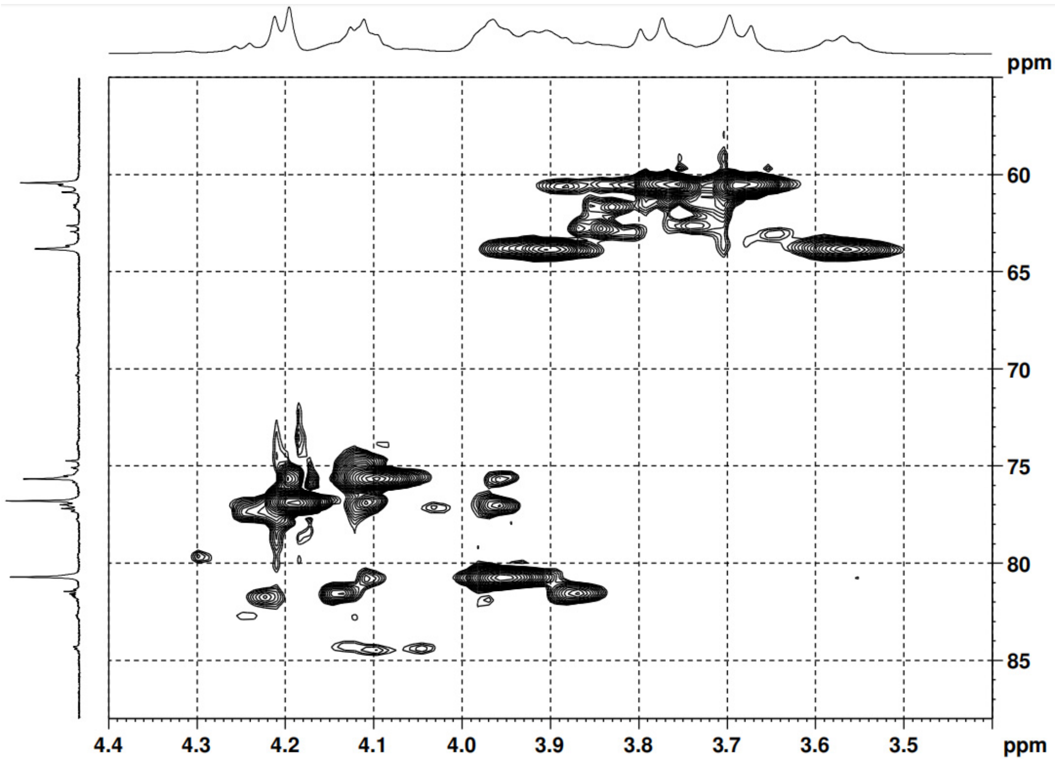

Figure S18 HSQC (D_2O) spectrum of **CDP2-2-2**

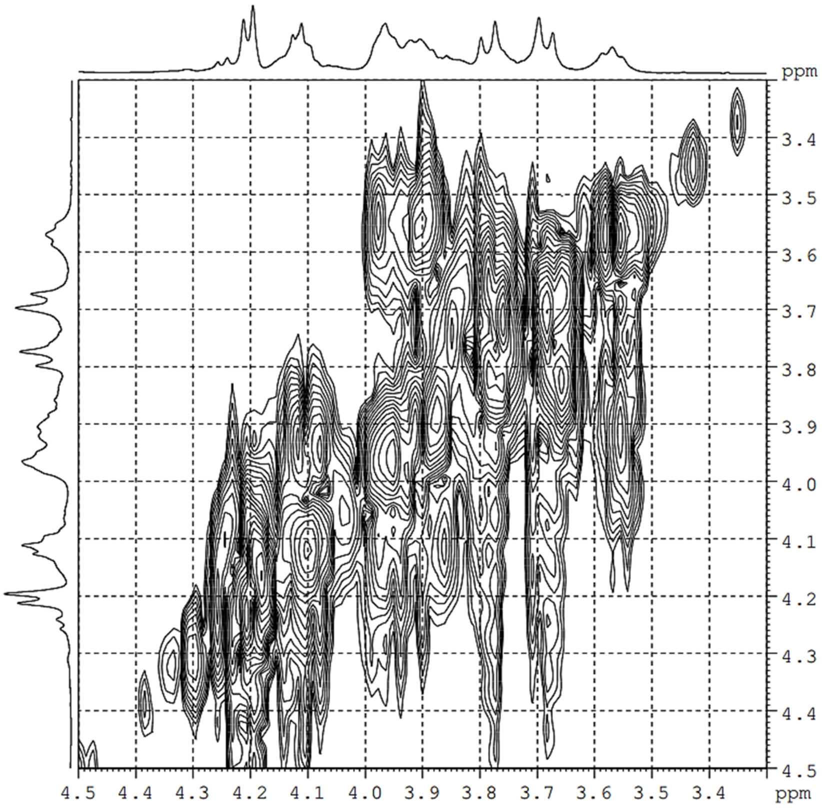

Figure S19 ^1H ^1H COSY (D_2O) spectrum of **CDP2-2-2**

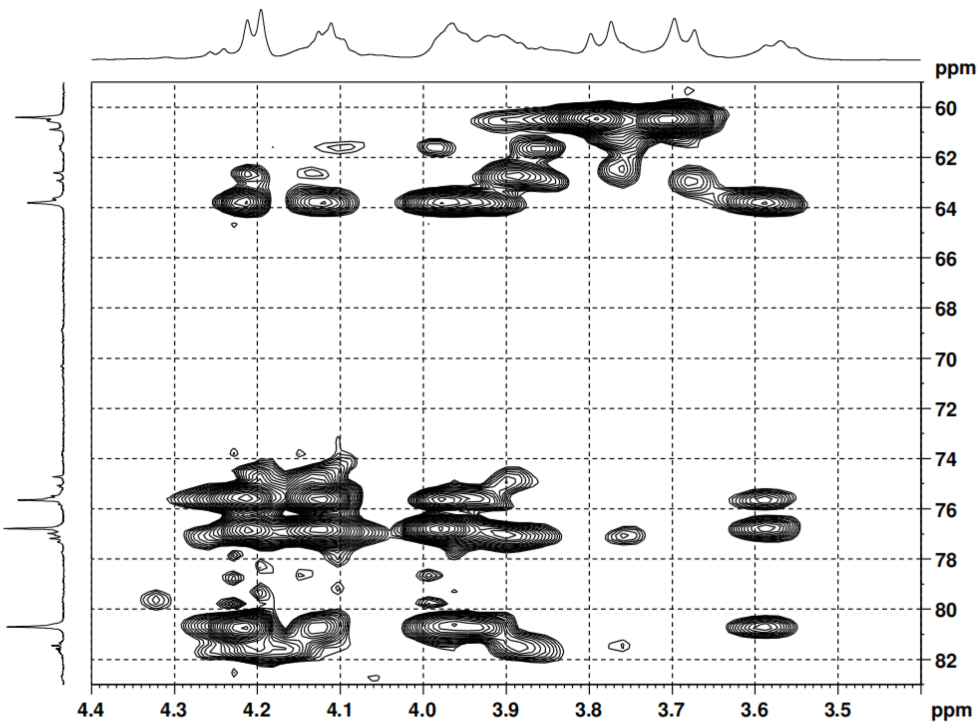

Figure S20 HSQC-TOCSY (D_2O) spectrum of **CDP2-2-2**

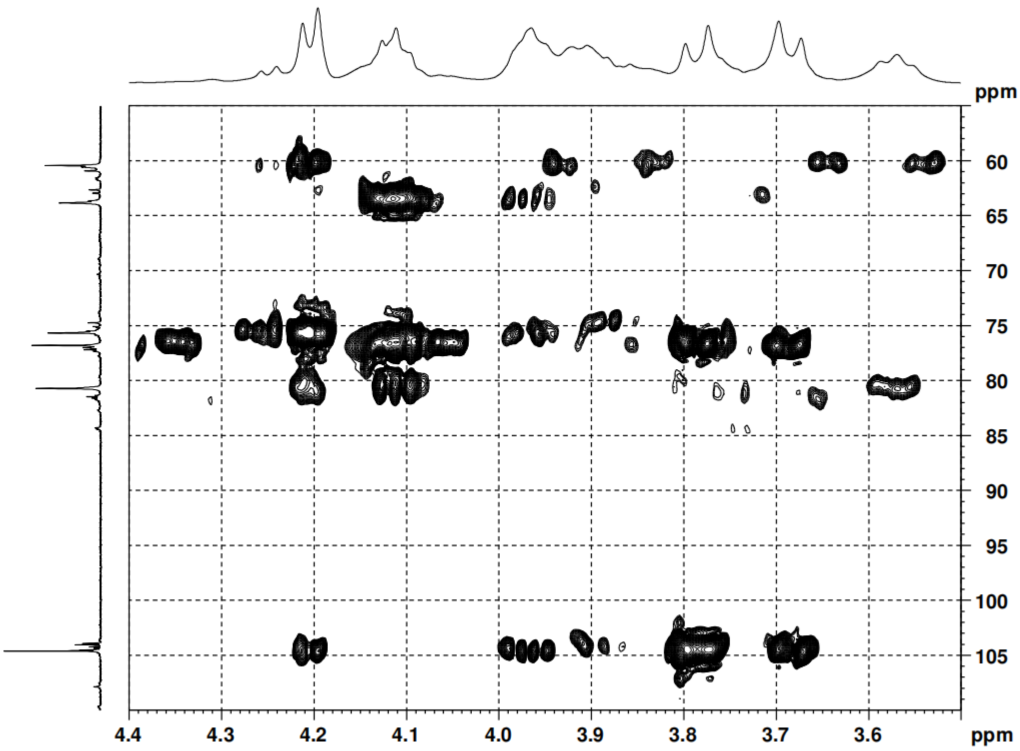

Figure S21 HMBC (D_2O) spectrum of **CDP2-2-2**

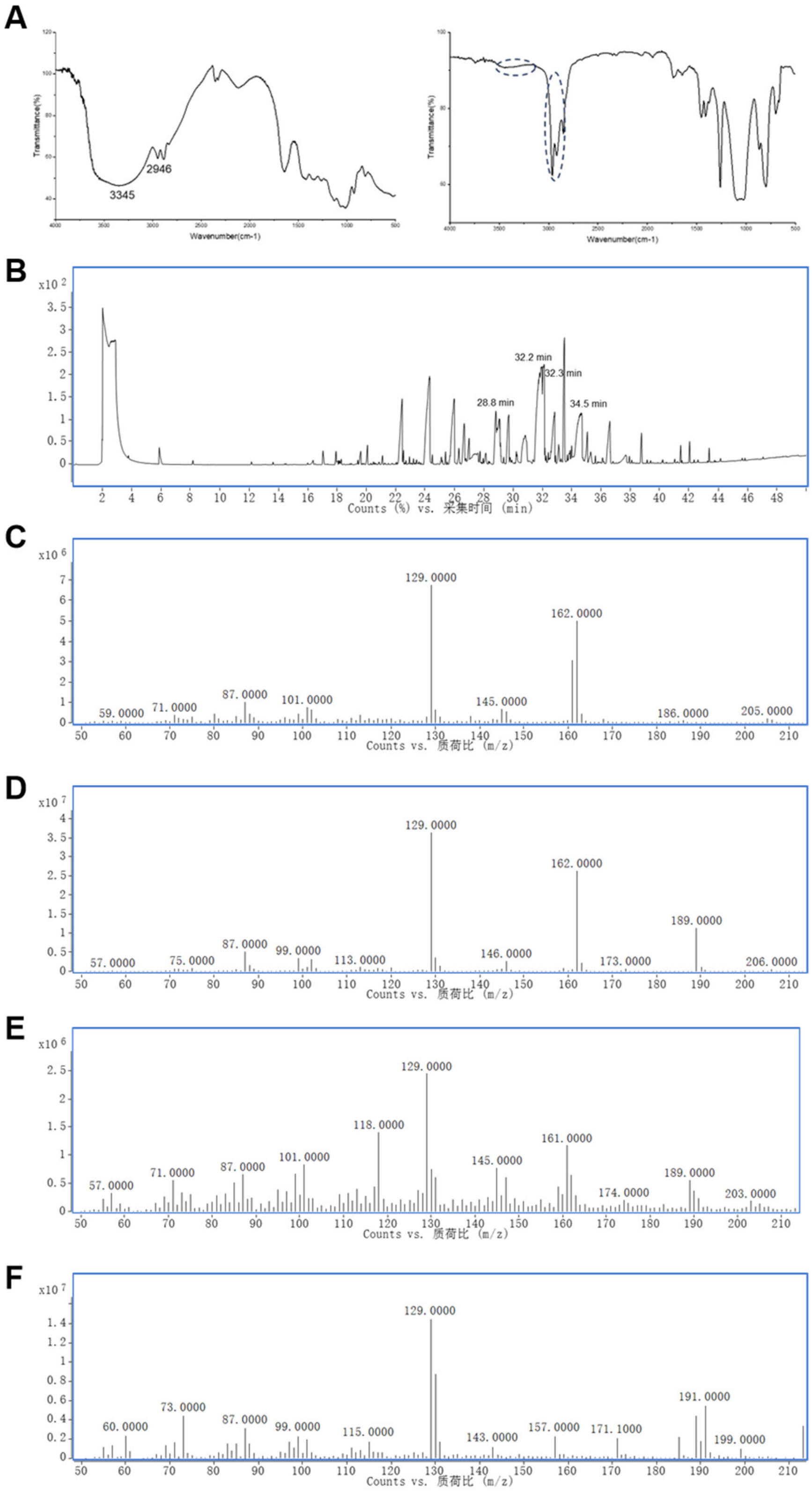

Figure S22 The FT-IR spectra before and after methylation reaction and GC-MS analysis results of **CDP2-2-2**

A: FT-IR spectra of **CDP2-2-2** before and after methylation; B: TIC of **CDP2-2-2**; C: spectrum of D-Fru*f*-(2→ (28.8 min); D: spectrum of →6)-D-Fru*f*-(2→ (32.2 min); E: spectrum of →1)-D-Fru*f*-(2→ (32.3 min); F: spectrum of →1,6)-D-Fru*f*-(2→ (34.5 min)

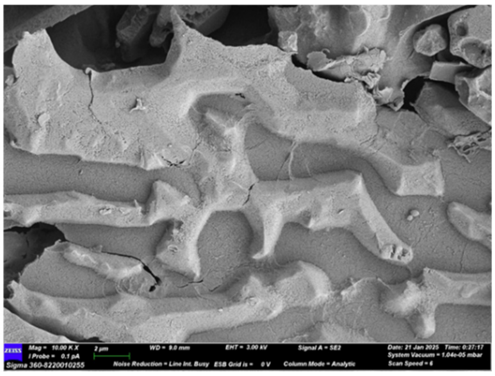

Figure S23 SEM images of **CDP2-2-2** (10000×)

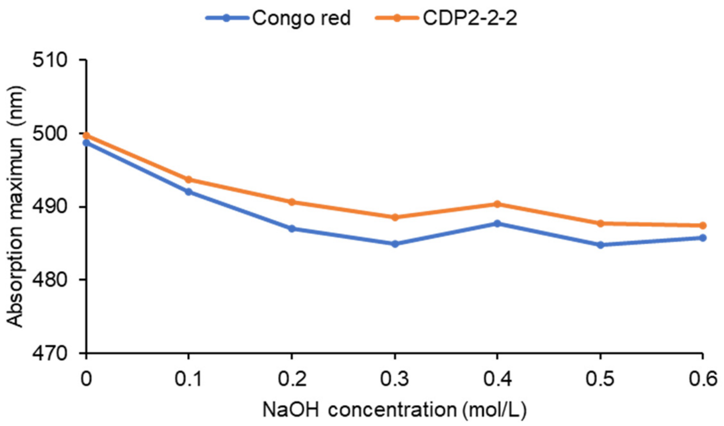

Figure S24 Congo red assay of **CDP2-2-2**

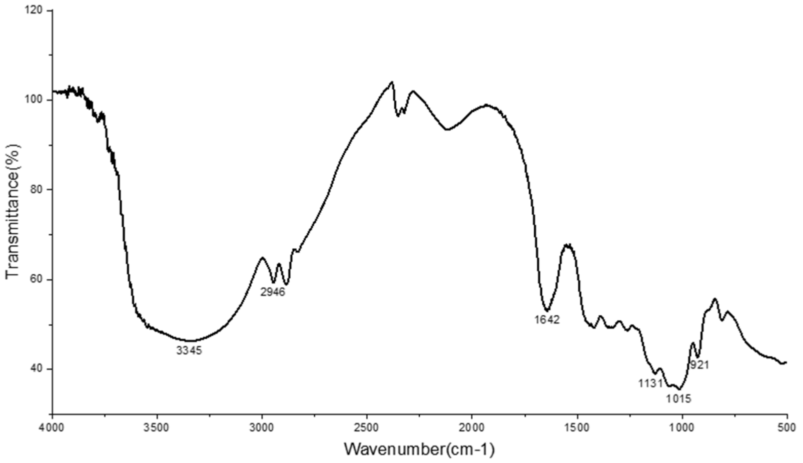

Figure S25 FT-IR spectrum of **CDP2-3-2**

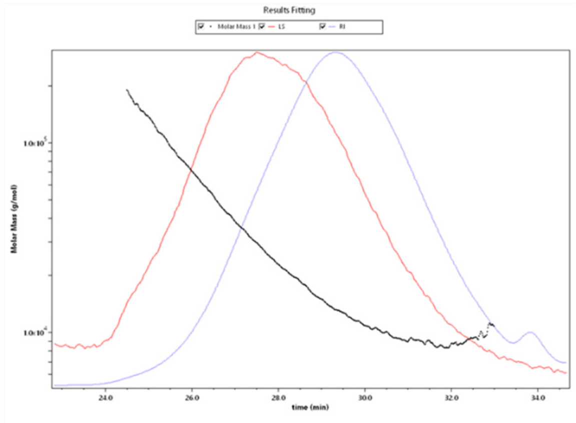

Figure S26 Molecular weight distribution plot of **CDP2-3-2**

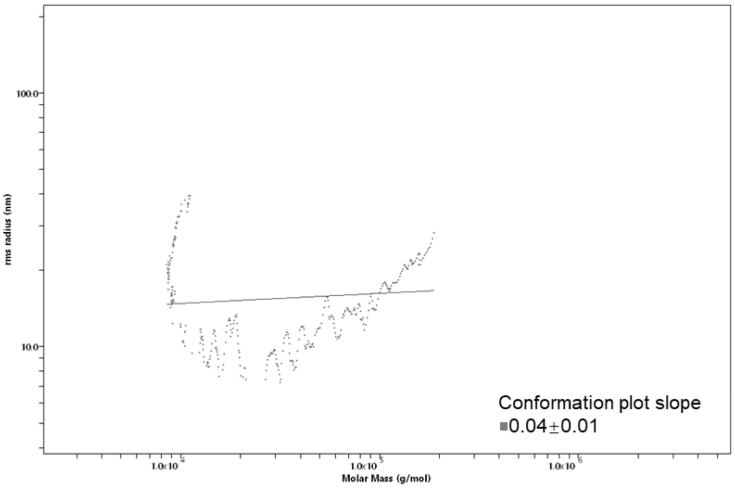

Figure S27 Molecular conformation analysis of **CDP2-3-2**

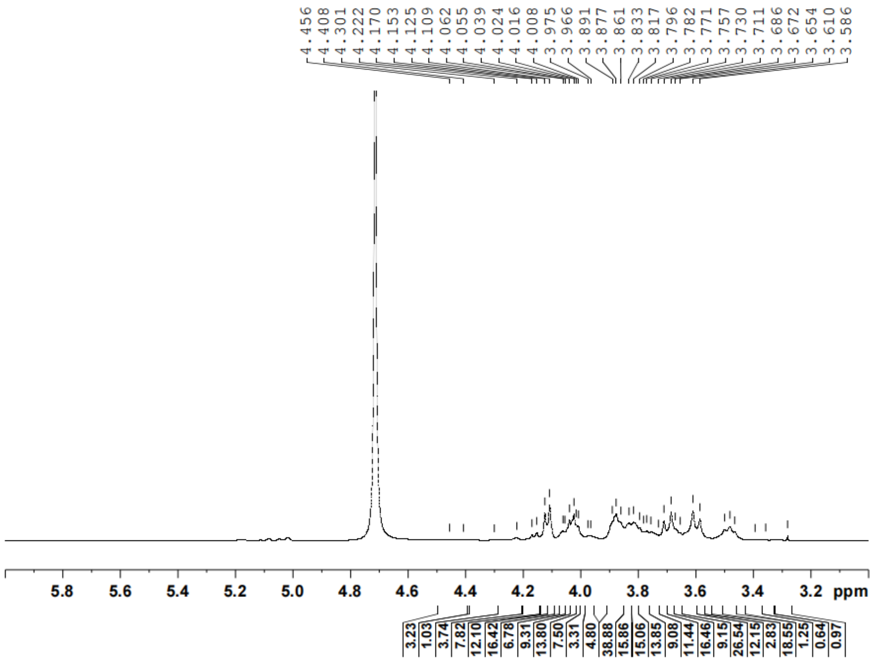

Figure S28 ^{1}H NMR spectrum of **CDP2-3-2** (500 MHz, D_2O)

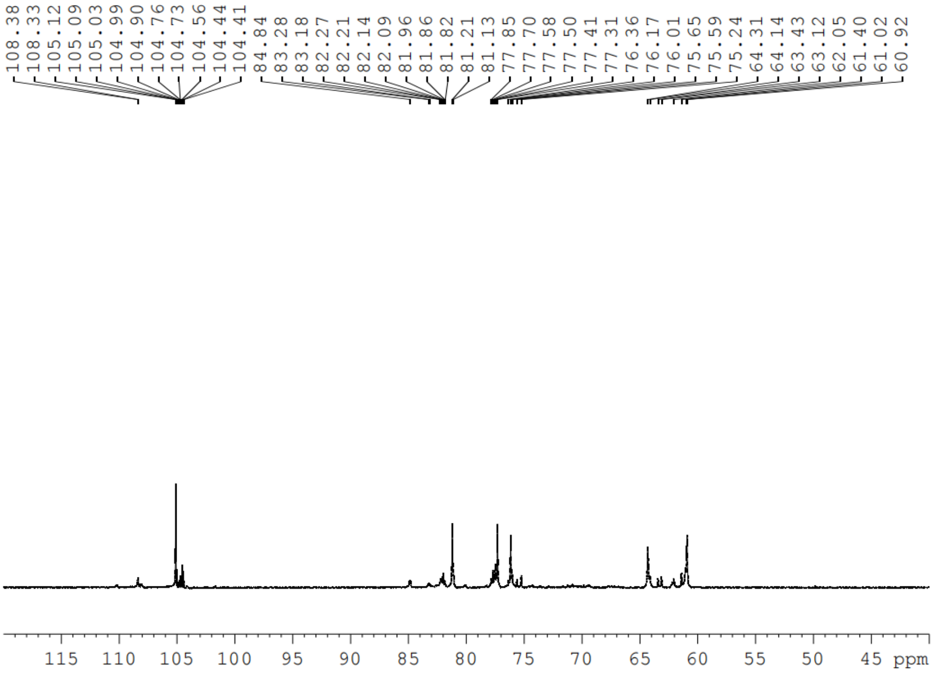

Figure S29 ^{13}C NMR spectrum of **CDP2-3-2** (125 MHz, D_2O)

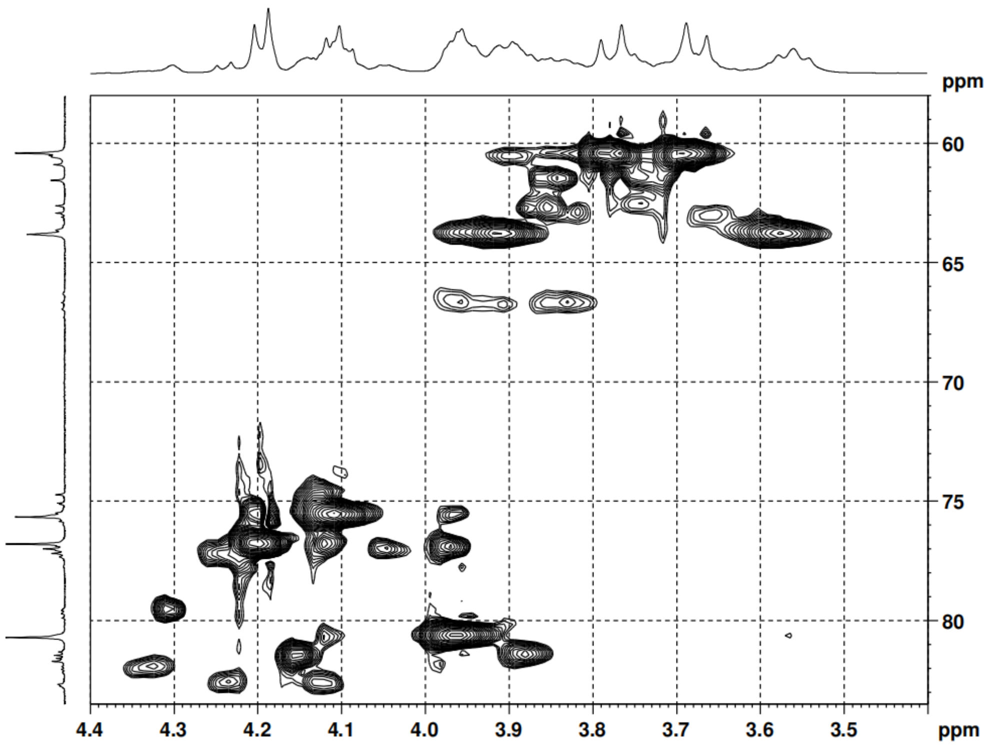

Figure S30 HSQC (D_2O) spectrum of **CDP2-3-2**

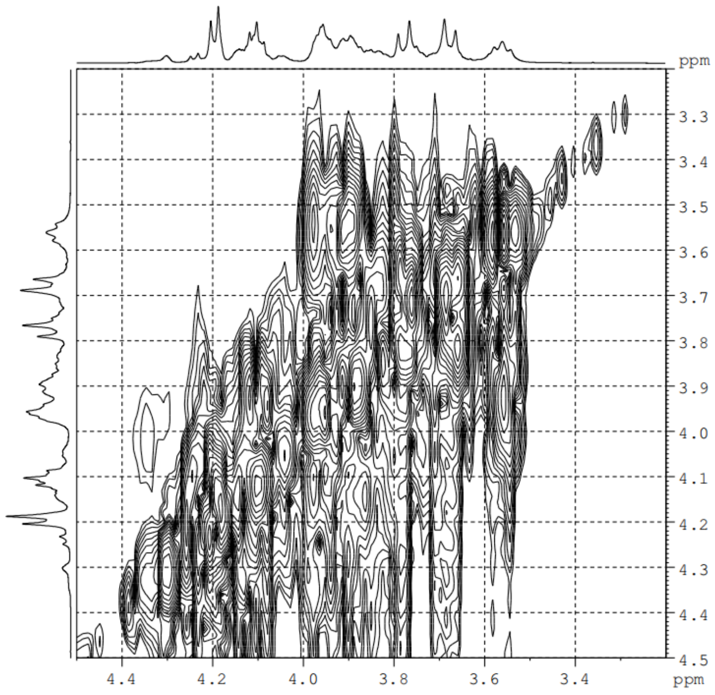

Figure S31 ^1H ^1H COSY (D_2O) spectrum of **CDP2-3-2**

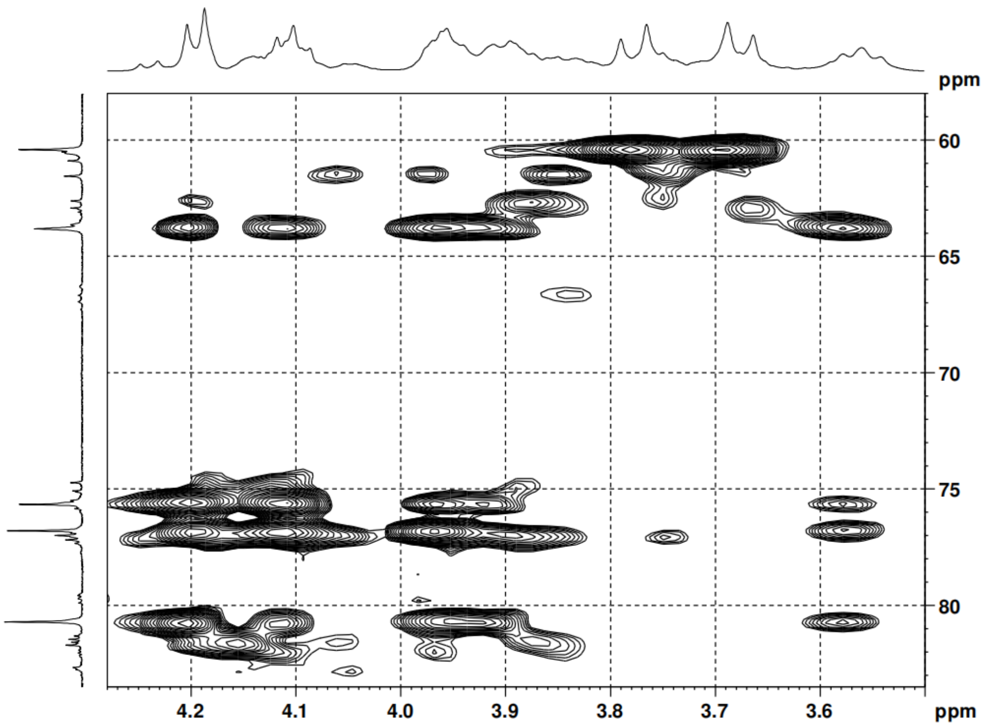

Figure S32 HSQC-TOCSY (D_2O) spectrum of **CDP2-3-2**

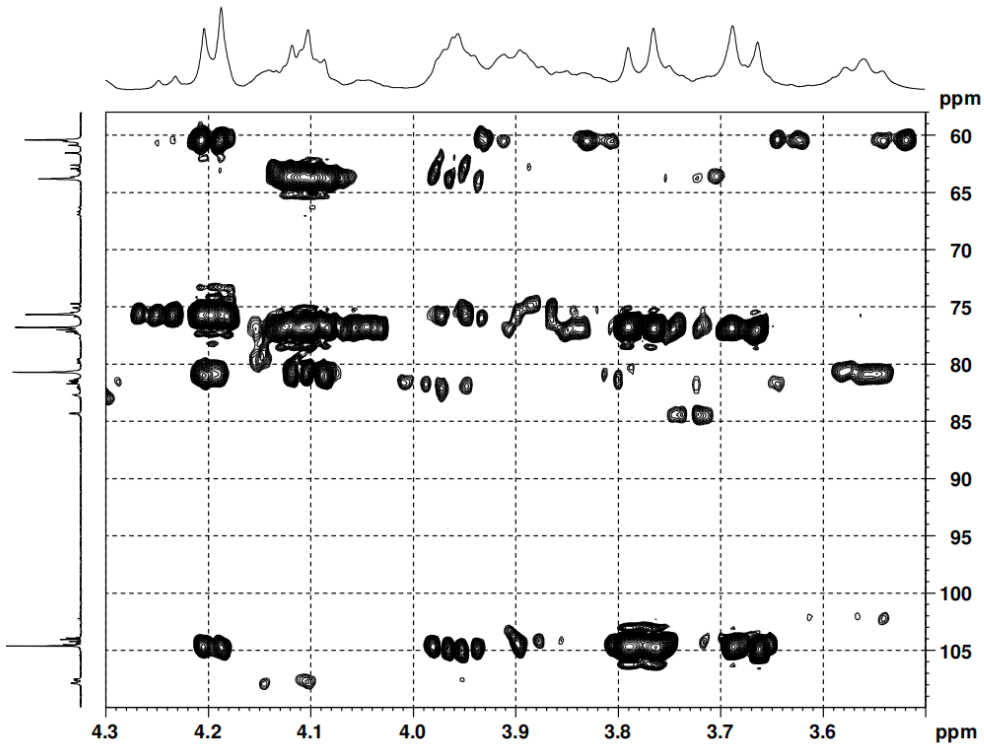

Figure S33 HMBC (D_2O) spectrum of **CDP2-3-2**

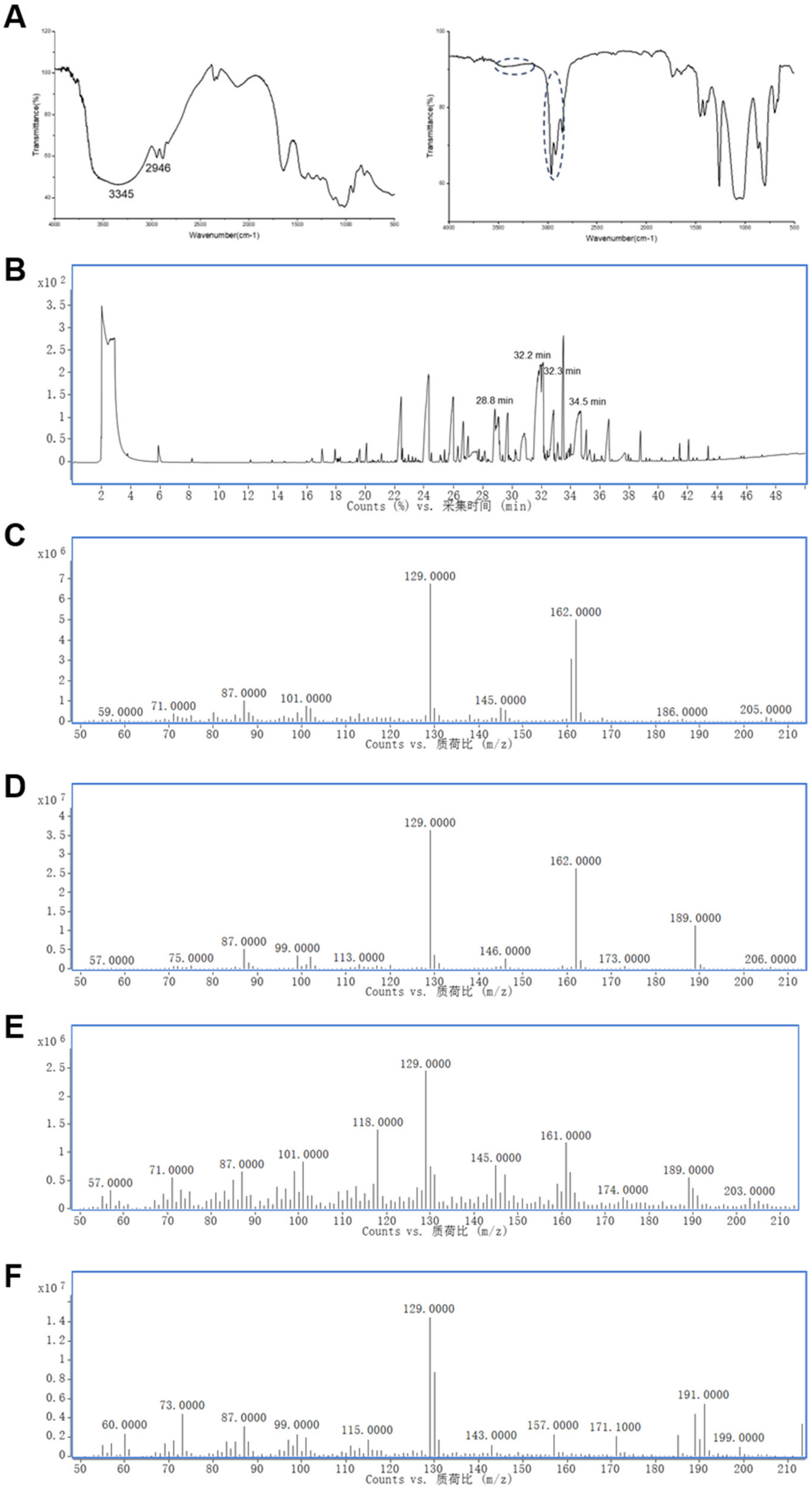

Figure S34 The FT-IR spectra before and after methylation reaction and GC-MS analysis results of **CDP2-3-2**

A: FT-IR spectra of **CDP2-3-2** before and after methylation; B: TIC of **CDP2-3-2**; C: spectrum of D-Fru*f*-(2→ (28.8 min); D: spectrum of →6)-D-Fru*f*-(2→ (32.2 min); E: spectrum of →1)-D-Fru*f*-(2→ (32.3 min); F: spectrum of →1,6)-D-Fru*f*-(2→ (34.5 min)

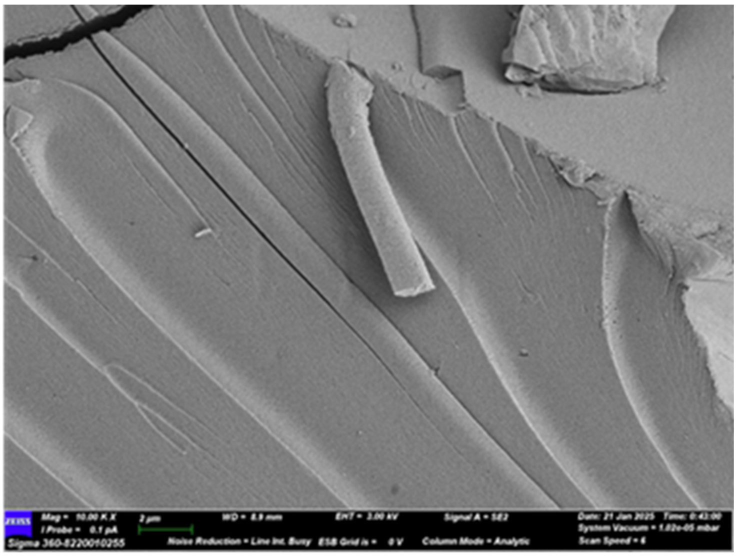

Figure S35 SEM images of **CDP2-3-2** (10000×)

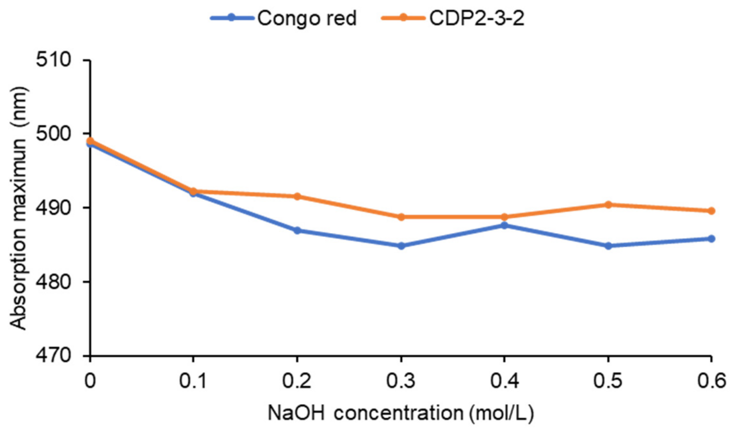

Figure S36 Congo red assay of **CDP2-3-2**

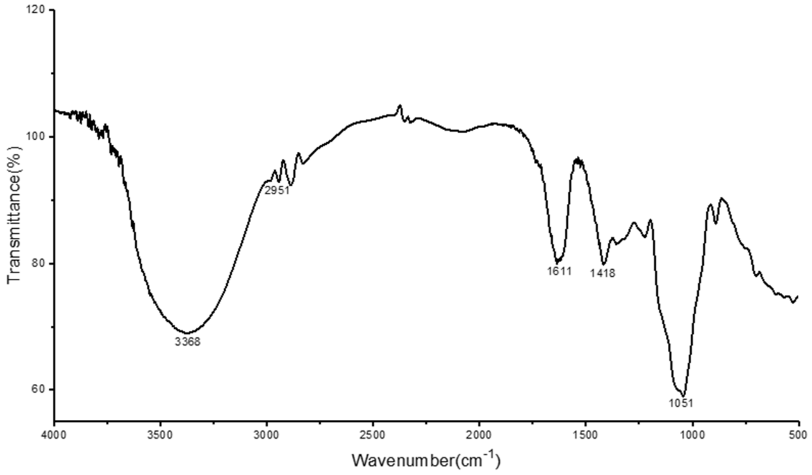

Figure S37 FT-IR spectrum of **CTP1-5-1**

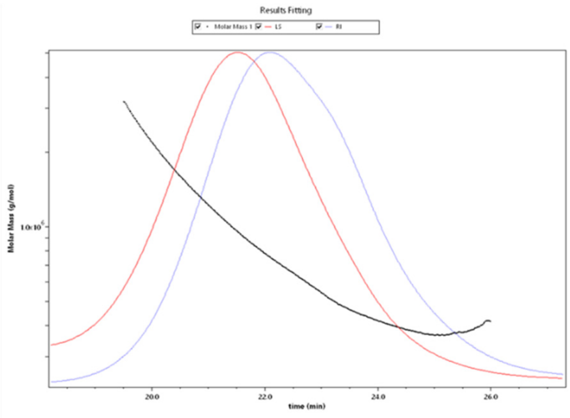

Figure S38 Molecular weight distribution plot of **CTP1-5-1**

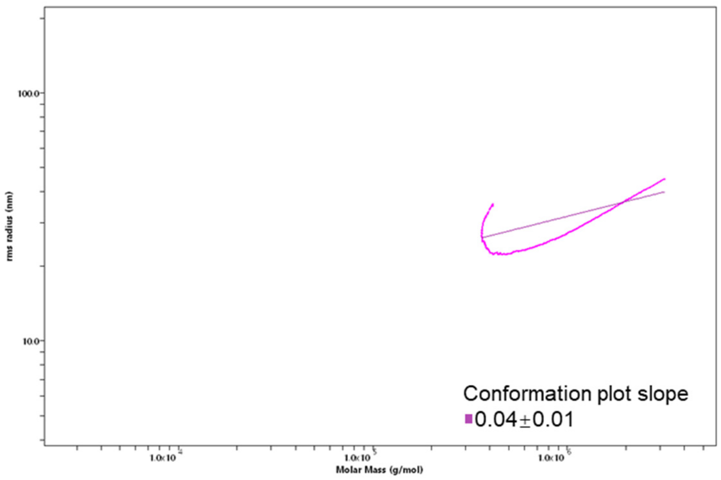

Figure S39 Molecular conformation analysis of **CTP1-5-1**

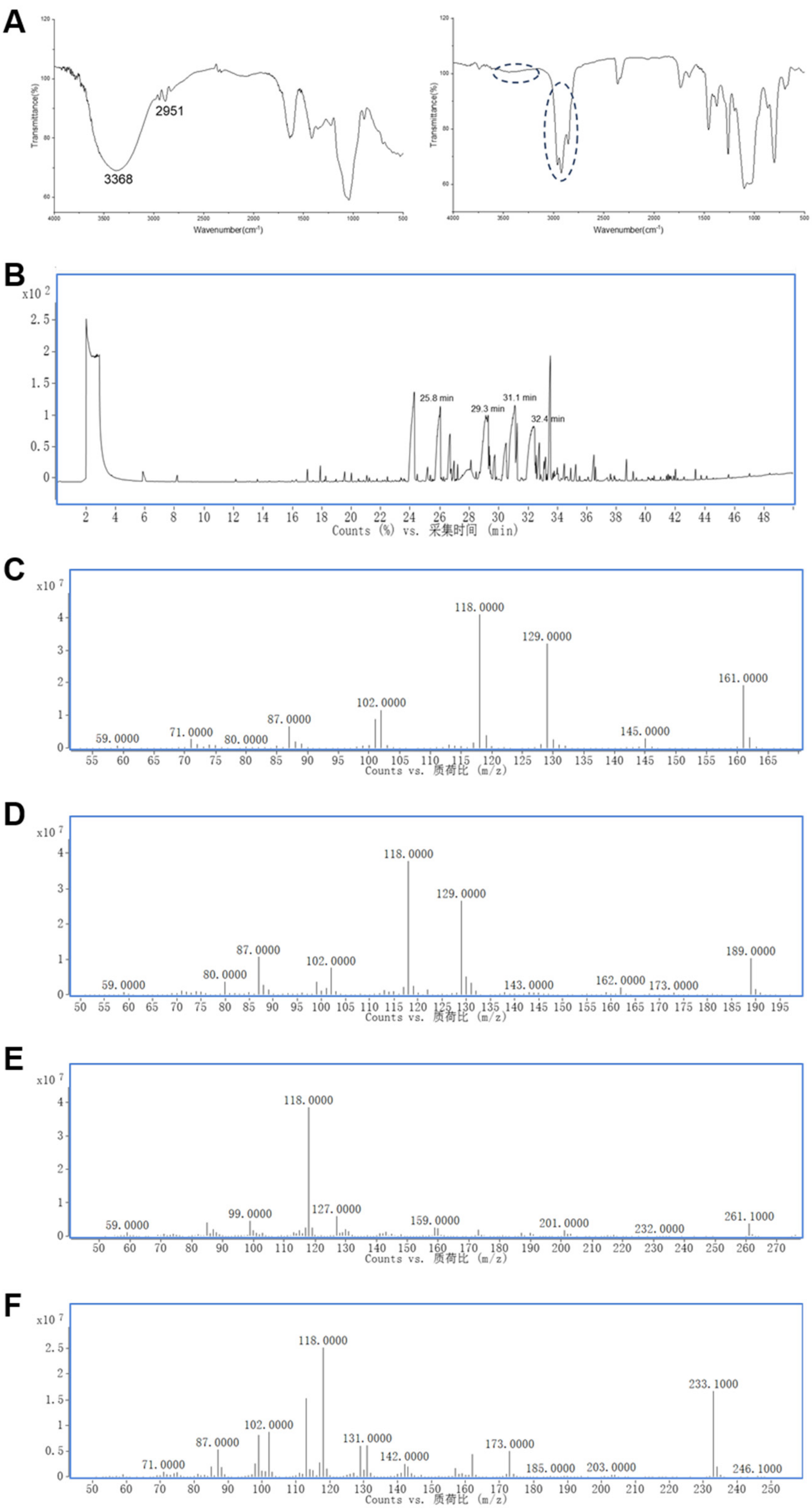

Figure S40 The FT-IR spectra before and after methylation reaction
and GC-MS analysis results of **CTP1-5-1**

A: FT-IR spectra of **CTP1-5-1** before and after methylation; B: TIC of **CTP1-5-1**; C: spectrum of t-L-Ara*f* (25.8 min); D: spectrum of →5)-L-Ara*f*-(1→ (29.3 min); E: spectrum of →3,5)-L-Ara*f*-(1→ (31.1 min); F: spectrum of →4)-D-Gal*p*-(1→ (32.4 min)

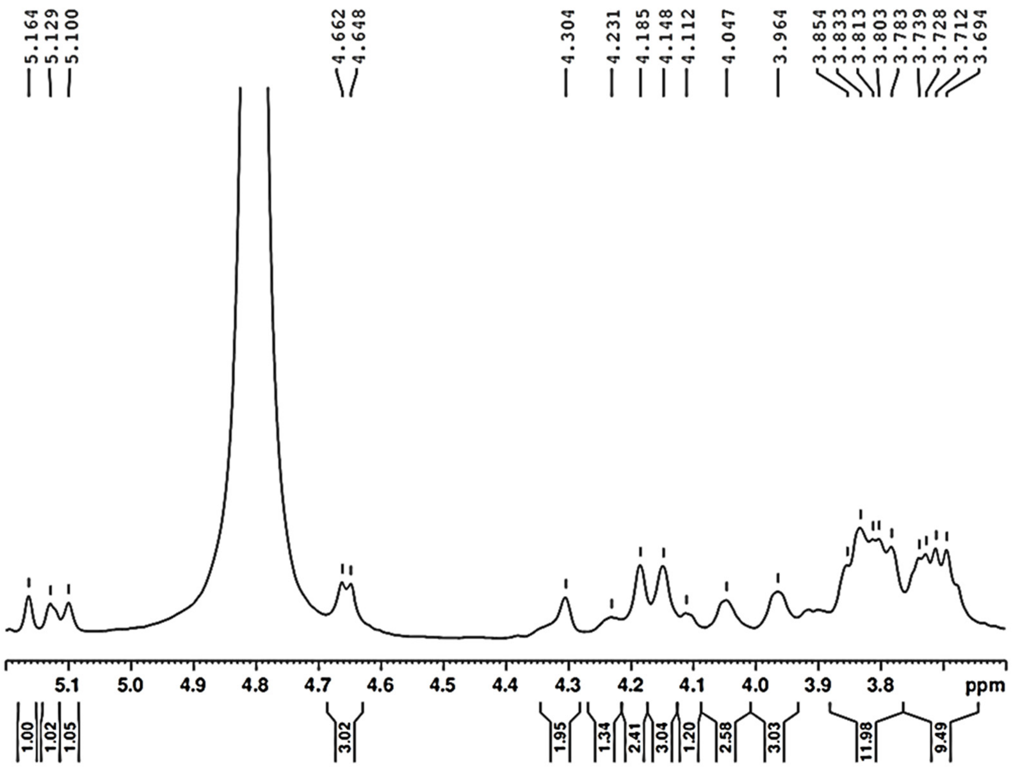

Figure S41 ^{1}H NMR spectrum of **CTP1-5-1** (500 MHz, D_2O)

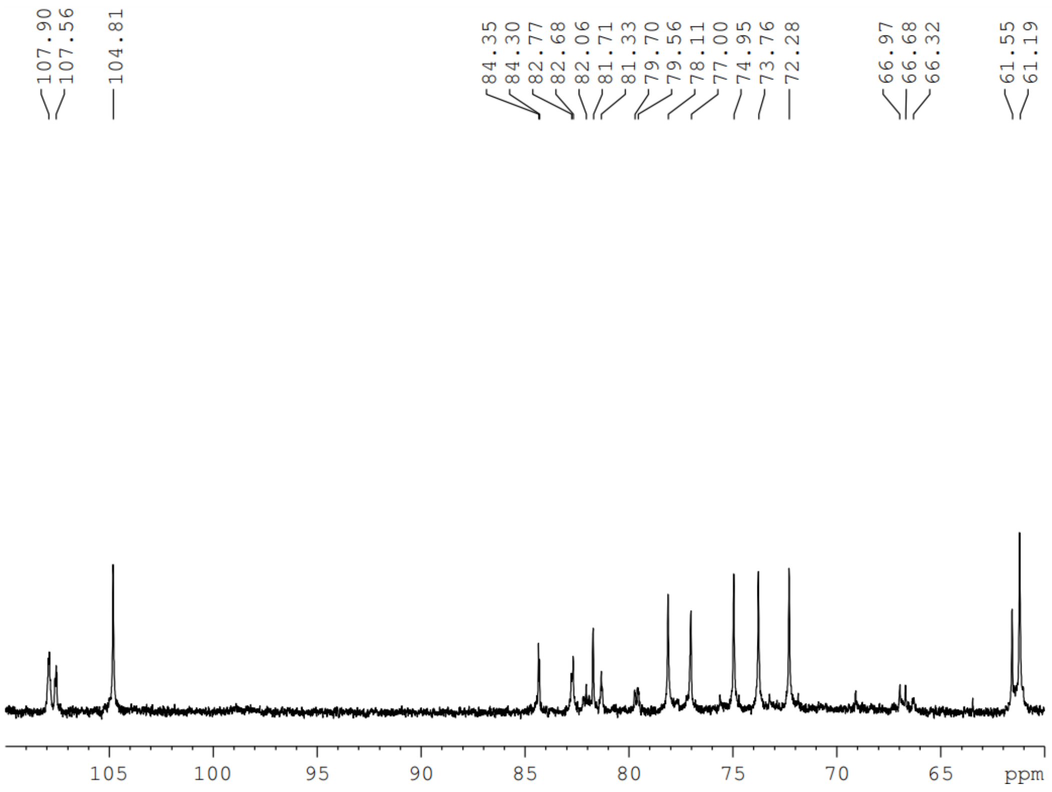

Figure S42 ^{13}C NMR spectrum of **CTP1-5-1** (125 MHz, D_2O)

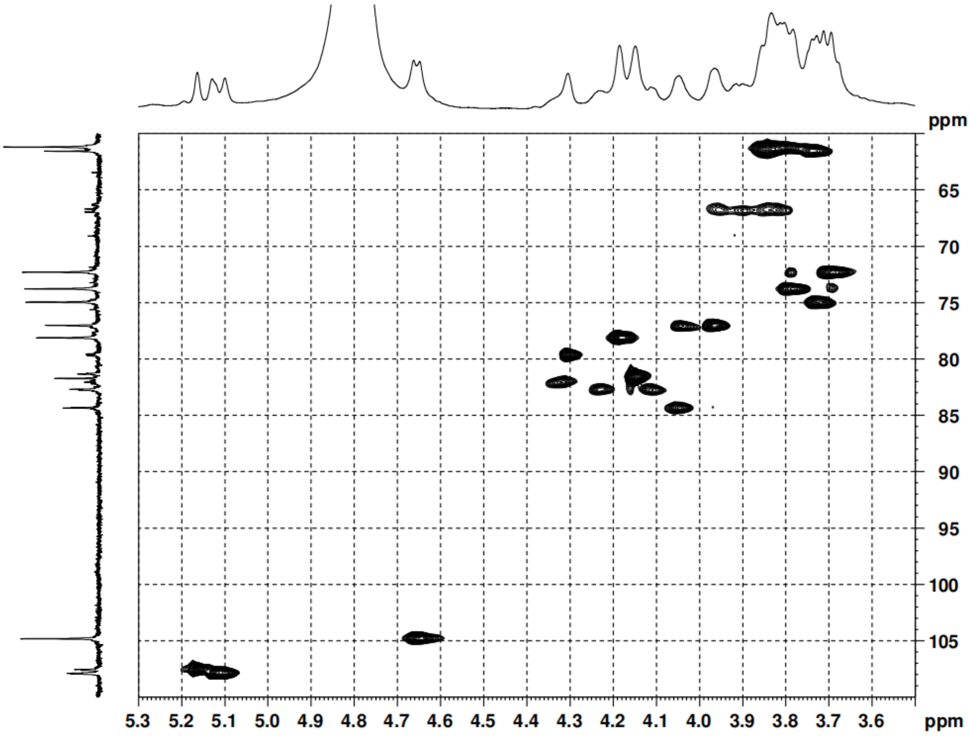

Figure S43 HSQC (D_2O) spectrum of **CTP1-5-1**

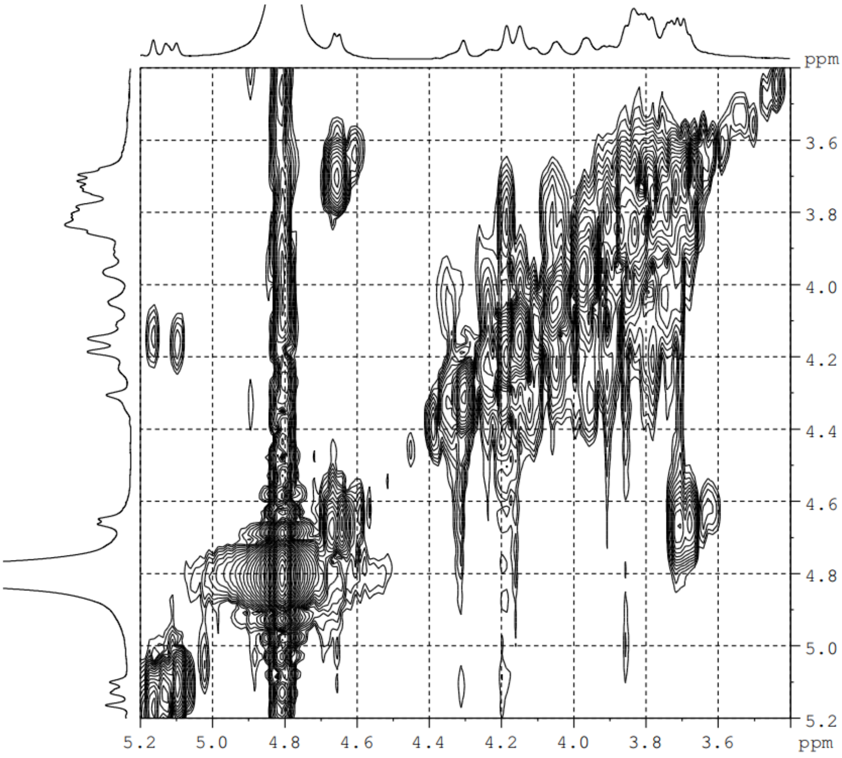

Figure S44 ^{1}H ^{1}H COSY (D_2O) spectrum of **CTP1-5-1**

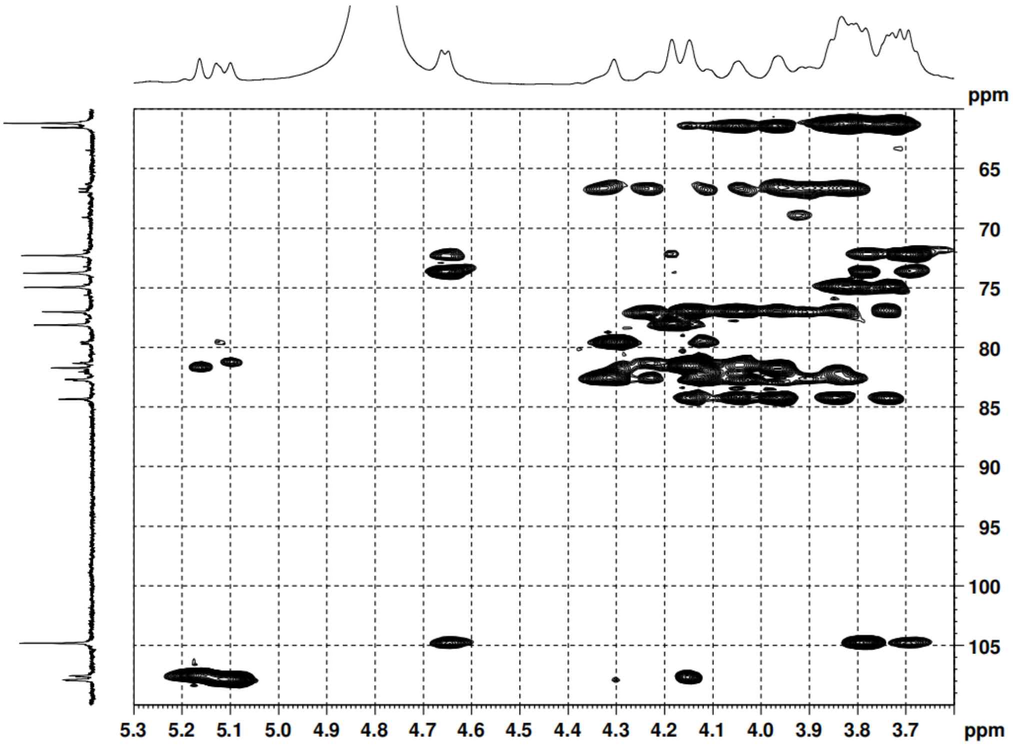

Figure S45 HSQC-TOCSY (D_2O) spectrum of **CTP1-5-1**

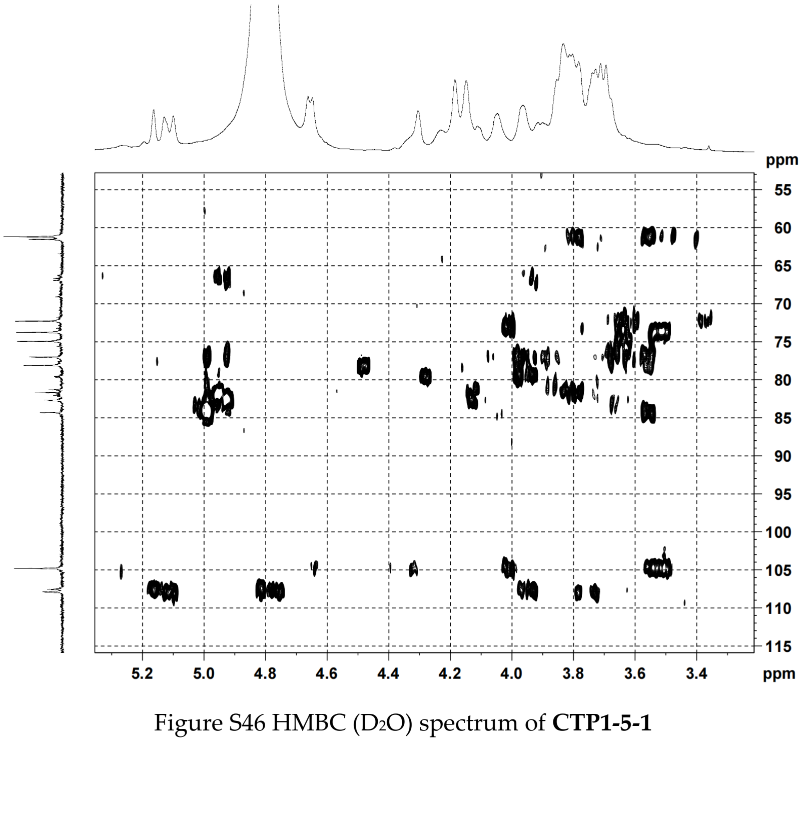

Figure S46 HMBC (D_2O) spectrum of **CTP1-5-1**

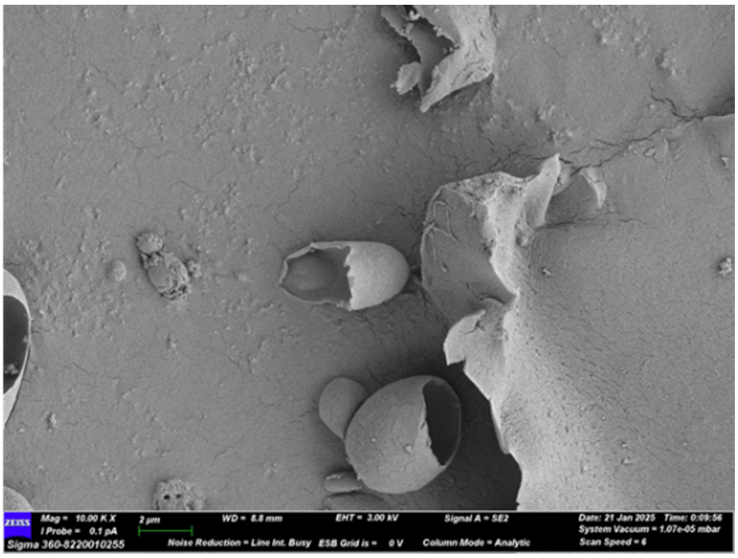

Figure S47 SEM images of **CTP1-5-1** (10000×)

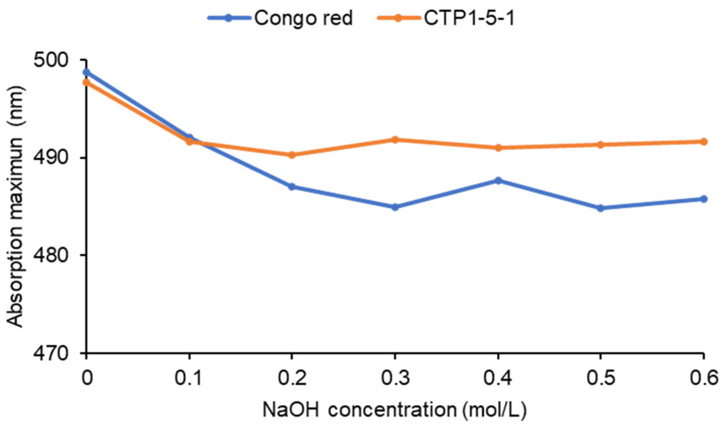

Figure S48 Congo red assay of **CTP1-5-1**

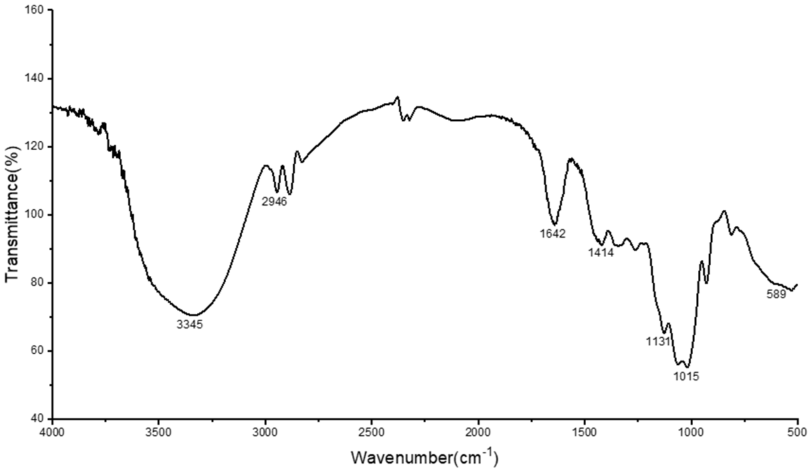

Figure S49 FT-IR spectrum of **CTP1-5-3**

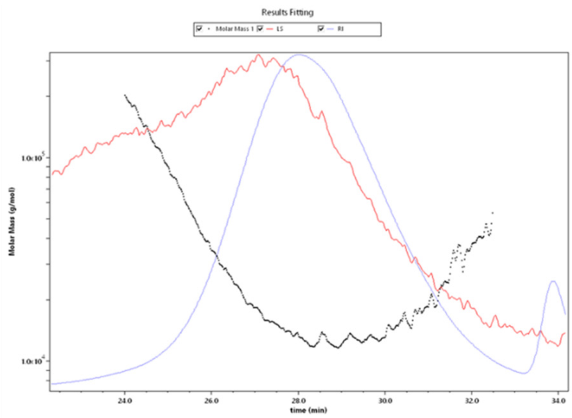

Figure S50 Molecular weight distribution plot of **CTP1-5-3**

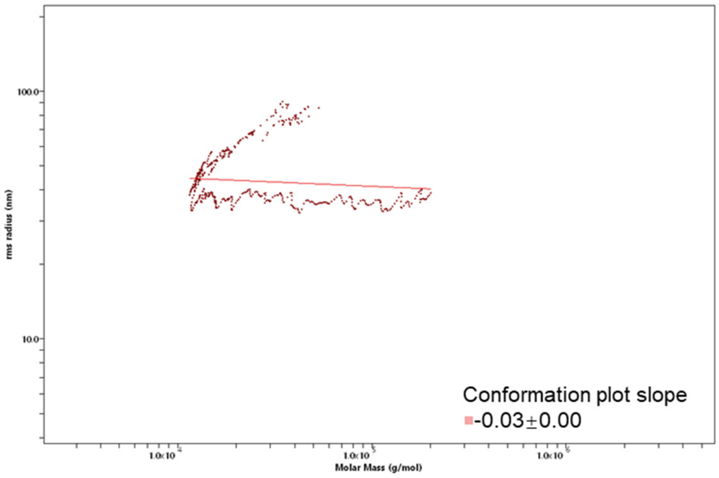

Figure S51 Molecular conformation analysis of **CTP1-5-3**

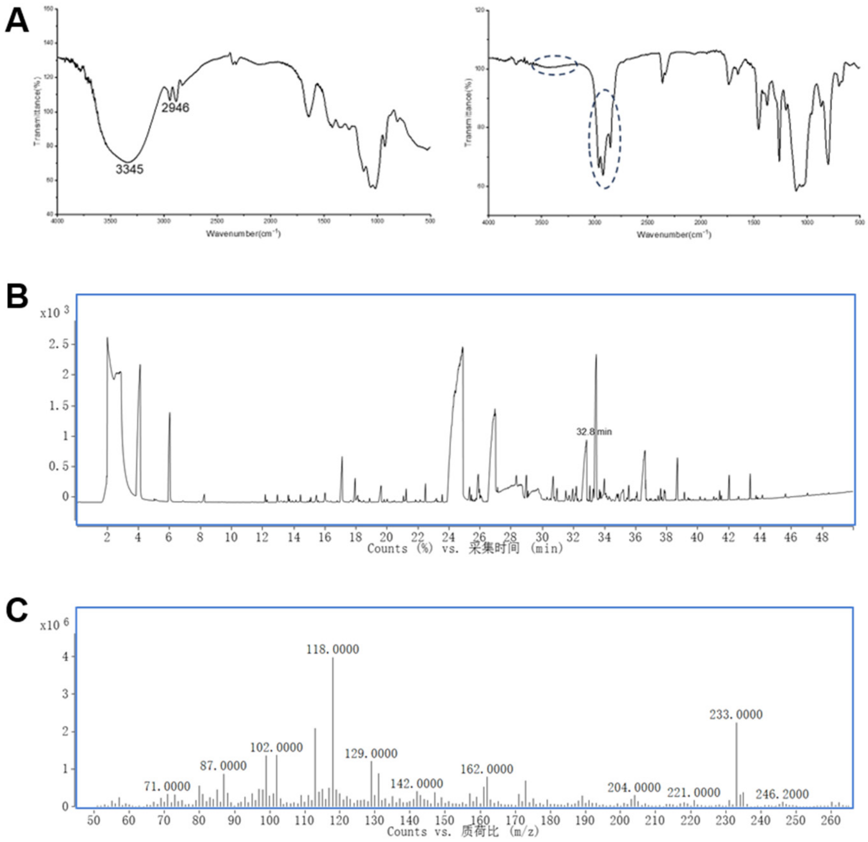

Figure S52 The FT-IR spectra before and after methylation reaction
and GC-MS analysis results of **CTP1-5-3**

A: FT-IR spectra of **CTP1-5-3** before and after methylation; B: TIC of **CTP1-5-3**; C: spectrum of →4)-D-Gal*p*-(1→ (32.8 min)

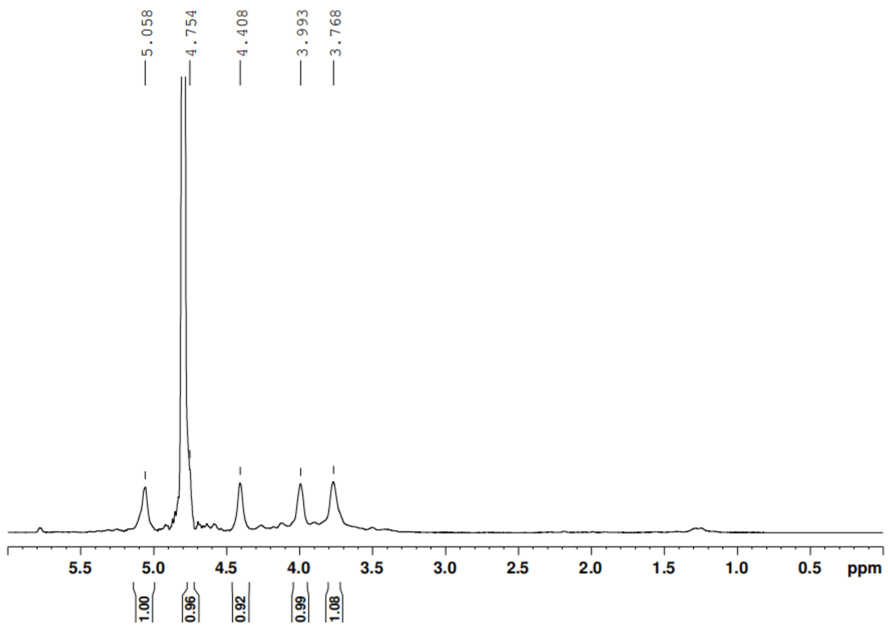

Figure S53 ^{1}H NMR spectrum of **CTP1-5-3** (500 MHz, D_2O)

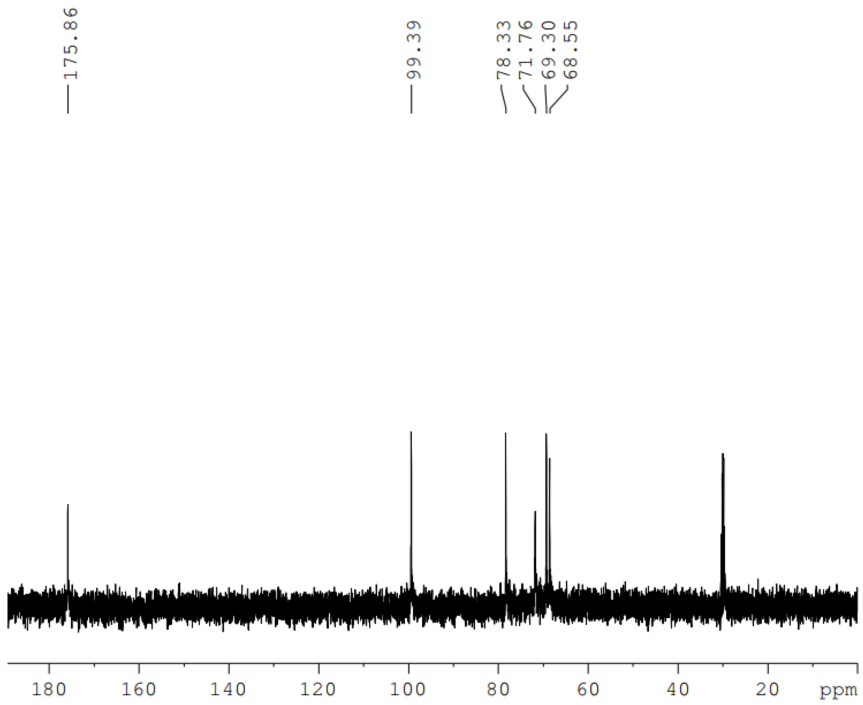

Figure S54 ^{13}C NMR spectrum of **CTP1-5-3** (125 MHz, D_2O)

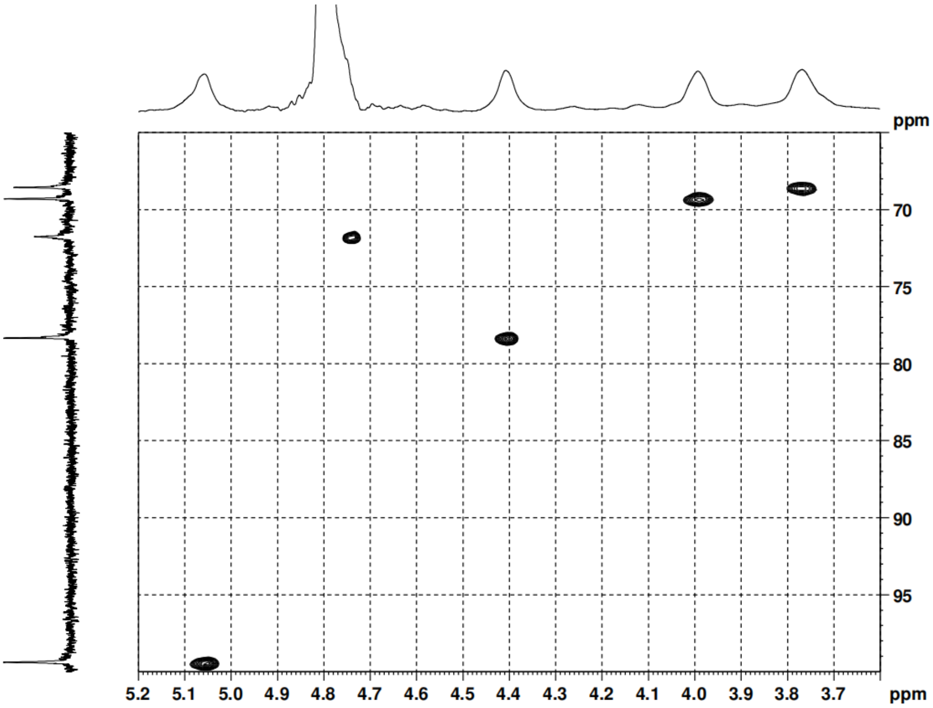

Figure S55 HSQC (D_2O) spectrum of **CTP1-5-3**

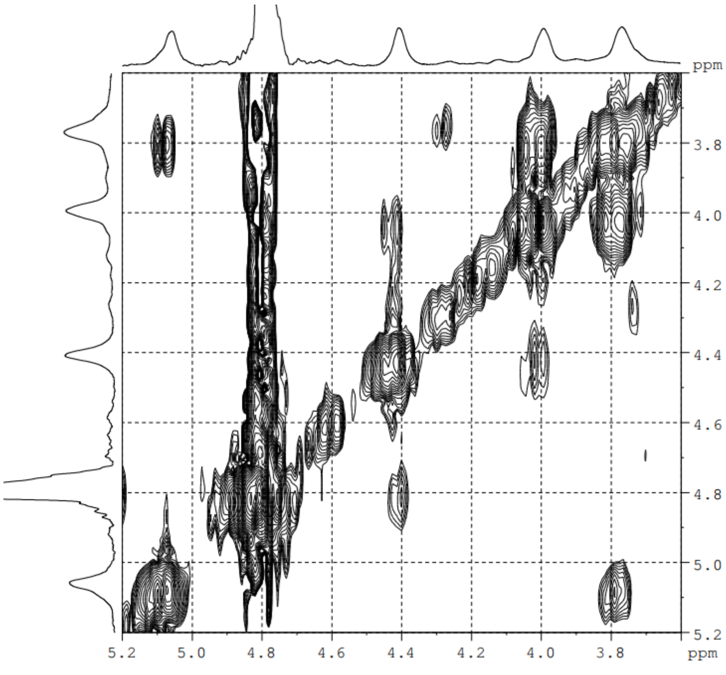

Figure S56 ^{1}H ^{1}H COSY (D_2O) spectrum of **CTP1-5-3**

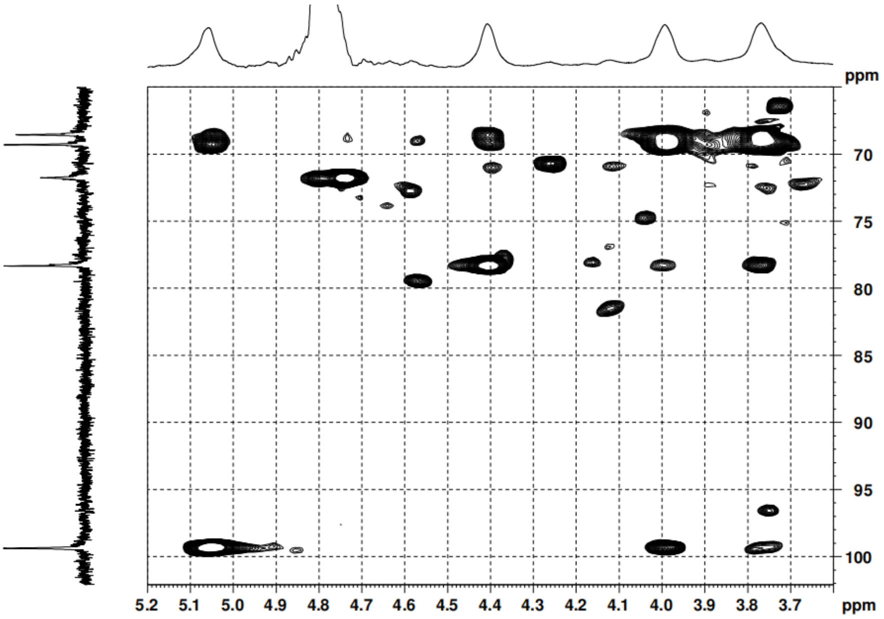

Figure S57 HSQC-TOCSY (D_2O) spectrum of **CTP1-5-3**

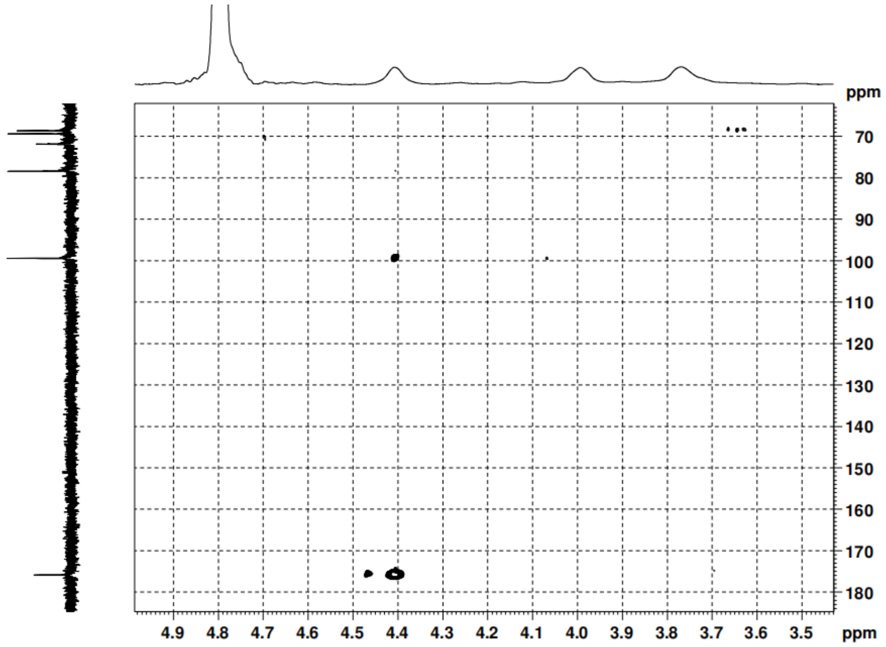

Figure S58 HMBC (D_2O) spectrum of **CTP1-5-3**

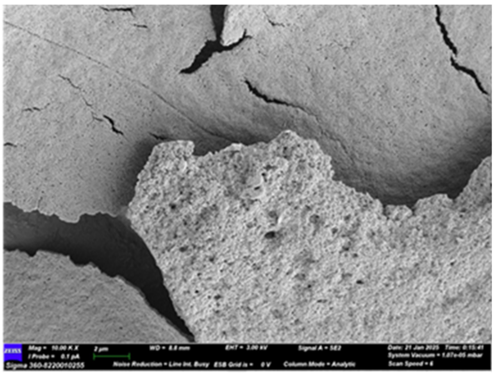

Figure S59 SEM images of **CTP1-5-3** (10000×)

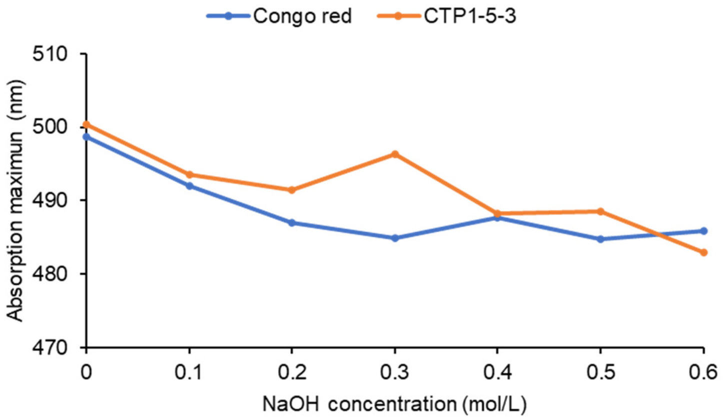

Figure S60 Congo red assay of **CTP1-5-3**

Table S1. Effects of **CDP1-5-1**, **CDP2-2-2**, **CDP2-3-2**, **CTP1-5-1**, and **CTP1-5-3** on cell viability and NO production of RAW264.7 cell

No.	Cell viability (%)	NO production (%)
**N**	100.0±4.7	100.0±3.9
**CDP1-5-1**	119.3±8.0*	3011.0±14.8***
**CDP2-2-2**	135.2±5.3**	208.8±9.0***
**CDP2-3-2**	119.3±9.1*	216.5±8.8***
**CTP1-5-1**	477.2±7.6***	1032.8±8.9***
**CTP1-5-3**	145.8±5.3**	400.7±19.8***
**LPS**	—	4108.4±18.8***

Note: the data was described as mean±SD. *$P < 0.05$; **$P < 0.01$; ***$P < 0.001$ (Differences between compound-treated group and normal group).
